# GATA6 Cooperates with EOMES/SMAD2/3 to Deploy the Gene Regulatory Network Governing Human Definitive Endoderm and Pancreas Formation

**DOI:** 10.1016/j.stemcr.2018.12.003

**Published:** 2019-01-08

**Authors:** Crystal Y. Chia, Pedro Madrigal, Simon L.I.J. Denil, Iker Martinez, Jose Garcia-Bernardo, Ranna El-Khairi, Mariya Chhatriwala, Maggie H. Shepherd, Andrew T. Hattersley, N. Ray Dunn, Ludovic Vallier

**Affiliations:** 1 Wellcome Trust Sanger Institute, Hinxton, Cambridge, UK; 2 Institute of Medical Biology, A^∗^STAR (Agency for Science, Technology and Research), 8A Biomedical Grove, #06-06 Immunos, 138648, Singapore; 3 Institute of Biomedical and Clinical Science, University of Exeter Medical School, Level 3 RILD Building, Barrack Road, Exeter EX25DW, UK; 4 Lee Kong Chian School of Medicine, Nanyang Technological University, 50 Nanyang Avenue, 639798, Singapore; 5 School of Biological Sciences, Nanyang Technological University, 60 Nanyang Drive, 637551, Singapore; 6 Wellcome Trust-Medical Research Council Cambridge Stem Cell Institute, Anne McLaren Laboratory for Regenerative Medicine, University of Cambridge, Cambridge, UK, and Department of Surgery, University of Cambridge, Cambridge, UK

**Keywords:** GATA6, definitive endoderm, pancreatic agenesis, human pluripotent stem cells, disease modeling

## Abstract

Heterozygous *de novo* mutations in *GATA6* are the most frequent cause of pancreatic agenesis in humans. In mice, however, a similar phenotype requires the biallelic loss of *Gata6* and its paralog *Gata4*. To elaborate the human-specific requirements for GATA6, we chose to model *GATA6* loss *in vitro* by combining both gene-edited and patient-derived pluripotent stem cells (hPSCs) and directed differentiation toward β-like cells. We find that *GATA6* heterozygous hPSCs show a modest reduction in definitive endoderm (DE) formation, while *GATA6*-null hPSCs fail to enter the DE lineage. Consistent with these results, genome-wide studies show that GATA6 binds and cooperates with EOMES/SMAD2/3 to regulate the expression of cardinal endoderm genes. The early deficit in DE is accompanied by a significant reduction in PDX1^+^ pancreatic progenitors and C-PEPTIDE^+^ β-like cells. Taken together, our data position GATA6 as a gatekeeper to early human, but not murine, pancreatic ontogeny.

## Highlights

• Heterozygous loss of human *GATA6* results in pancreatic agenesis
• *GATA6*^+/−^ hPSCs form fewer pancreatic progenitors and β cells *in vitro*
• *GATA6*^−/−^ hPSCs entirely fail to form the definitive endoderm *in vitro*
• *GATA6* binds to and cooperates with EOMES/SMAD2/3 to regulate cardinal endodermal genes

## Teaser

In this article, Chia et al. model human pancreatic agenesis *in vitro* that results from heterozygous loss of the *GATA6* gene. Using both gene-edited and patient-derived hPSCs, they demonstrate that *GATA6* serves as a gatekeeper to early human, but not murine, pancreatic ontogeny. Genome-wide studies further show that GATA6 binds to and cooperates with EOMES/SMAD2/3 to regulate the expression of cardinal endoderm genes.

## Introduction

Pancreatic agenesis is an extremely rare human condition resulting from the impaired formation of the pancreas during embryonic development. Clinically, patients can entirely lack the pancreas or present with only a partially formed organ (hypoplasia). The majority of patients have complete absence of a functioning pancreas, resulting in intrauterine growth retardation, neonatal diabetes, and exocrine pancreatic failure, and thus require insulin and exocrine enzyme replacement therapy. Less commonly, less severely affected patients can display a reduction in total islet number or insulin-secreting β cells and present diabetic symptoms during adolescence or adulthood.

The vast majority of human pancreatic agenesis cases owe their genetic origins to mutations in a small handful of pancreatic regulatory genes. The first described is *Pancreatic and Duodenal Homeobox 1* (*Pdx1*) (Schwitzgebel, 2014, Stoffers et al., 1997). In mice, *Pdx1* transcripts label the incipient pancreatic primordium—two epithelial buds that are situated dorsally and ventrally on opposite sides of the posterior foregut around embryonic day 9.5 (Jørgensen et al., 2007, Pan and Wright, 2011). In *Pdx1*-null mutant mice, these buds initially form but quickly regress, resulting in complete pancreatic agenesis, severe hyperglycemia, and death within a few days of birth (Ahlgren et al., 1996, Jonsson et al., 1994, Offield et al., 1996). PDX1 similarly labels the human embryonic dorsal and ventral foregut around Carnegie stage 12 (29–31 days post conception) (Jennings et al., 2013). Significantly, the pathology of human patients with homozygous or compound heterozygous mutations in *PDX1* mirrors the agenesis phenotype observed in *Pdx1*-deficient mice (Schwitzgebel et al., 2003, Stoffers et al., 1997).

The most common cause of pancreatic agenesis in humans is heterozygous mutations in the *GATA6* gene (De Franco et al., 2013, Lango Allen et al., 2011). *GATA6* encodes a highly conserved zinc-finger transcription factor that recognizes and binds the (A/T)GATA(A/G) regulatory motif, two of which are located in the mouse *Pdx1* and human *PDX1* promoters (Carrasco et al., 2012, Lentjes et al., 2016, Patient and McGhee, 2002, Viger et al., 2008, Xuan et al., 2012). GATA6, along with its five other family members (GATA1–5), functions in diverse cellular contexts, from coordinating morphogenesis during embryonic development to the maintenance of lineage-specific gene expression in adult hematopoietic stem cells (Lentjes et al., 2016, Viger et al., 2008). *Gata6* is expressed in the definitive endoderm (DE) that emerges during gastrulation, as well as its derivative the gut tube epithelium and the early pancreas primordium (Freyer et al., 2015, Morrisey et al., 1996). *Gata6* expression persists as the pancreas undergoes branching morphogenesis, becoming restricted in later development to the ductal epithelial compartment and a subset of endocrine cells (Decker et al., 2006, Ketola et al., 2004).

In contrast to *PDX1*, *GATA6* mutations that result in pancreatic agenesis are heterozygous and predominantly *de novo* (Chao et al., 2015, De Franco et al., 2013, Lango Allen et al., 2011, Stanescu et al., 2015, Suzuki et al., 2014). The majority of cases have full pancreatic agenesis, but there are some associated with incomplete penetrance, resulting in a broad spectrum of clinical manifestations (De Franco et al., 2013). At the extreme, family members with the same inherited *GATA6* allele can present with markedly different phenotypes (Bonnefond et al., 2012, Yau et al., 2017, Yorifuji et al., 2012). In addition, *GATA6* patients usually display a number of extrapancreatic abnormalities, including congenital heart defects, as well as several whose origins are endodermal—hepatobiliary malformations, gall bladder agenesis, and gut herniation (Chao et al., 2015, De Franco et al., 2013, Lango Allen et al., 2011).

Given the observations that haploinsufficiency results in severe pancreatic and non-pancreatic anomalies in humans, it is surprising that *Gata6* heterozygous null mice are viable and fertile, with no reported abnormalities (Koutsourakis et al., 1999, Morrisey et al., 1998). In a recent study, Schrode et al. (2014) showed that the specification of the extraembryonic primitive endoderm entirely fails in *Gata6* homozygous embryos at the blastocyst stage, while in a series of older reports *Gata6*-null mutant embryos were recovered at post-implantation stages with defects in the cardiac mesoderm and visceral endoderm (Koutsourakis et al., 1999, Morrisey et al., 1998). Interestingly, tetraploid complementation experiments between wild-type embryos and *Gata6*-deficient embryonic stem cells, a technique that overcomes the early lethality resulting from the absence of Gata6 in the extraembryonic lineages, reveal that *Gata6*-deficient cells can indeed contribute descendants to the DE in chimeric embryos (Zhao et al., 2005). Moreover, conditional loss of *Gata6* specifically in Pdx1^+^ pancreatic progenitors has no impact on pancreatic morphogenesis. Only when a closely related gene, *Gata4*, is simultaneously deleted is an agenesis phenotype recovered that resembles *GATA6* heterozygous human patients (Carrasco et al., 2012, Xuan et al., 2012).

The striking discrepancy between the mouse and the human phenotypes and the complex genetic landscape of *GATA6* agenesis patients led us to model *GATA6* deficiency *in vitro* using human pluripotent stem cells (hPSCs). We generated a large panel of heterozygous, homozygous, and compound heterozygous *GATA6* mutations by performing genome editing in human embryonic stem cells (hESCs) and human induced pluripotent stem cells (hiPSCs). We additionally derived hiPSCs from two *GATA6* heterozygous pancreatic agenesis patients. Subjecting these *GATA6* heterozygous hPSCs to directed differentiation into the pancreatic lineage unexpectedly revealed a modest requirement for wild-type *GATA6* gene dosage for robust formation of the DE. In contrast to the mouse, complete loss of *GATA6* abrogates DE production. Consistent with these results, genome-wide studies show that GATA6 binds and cooperates with EOMES/SMAD2/3 to regulate the expression of cardinal endoderm genes. In addition, *GATA6* haploinsufficiency diminishes the ability of those DE cells that form to become PDX1^+^ pancreatic progenitors and to further mature into C-PEPTIDE-containing β-like cells. These findings show that in humans, the formation of DE and acquisition of pancreatic fate are exquisitely sensitive to *GATA6* gene dosage.

## Results

### *GATA6* Expression during Directed Differentiation of hPSCs into the Endocrine Lineage

Consistent with *Gata6* expression in the mouse embryo, we previously showed that *GATA6* is activated during the early differentiation of hESCs into the DE lineage (Teo et al., 2015, Vallier et al., 2009). We next determined the precise expression kinetics of *GATA6* during extended differentiation into the pancreatic lineage using the well-characterized hESC line H9 and a slightly revised version of an 18-day chemically defined protocol previously published by our group (Figure S1A and see Experimental Procedures for complete details) (Cho et al., 2012). *GATA6* transcripts are not detected in undifferentiated hESCs, but are abundant by day 3, a time point characterized by the expression of canonical DE markers (*SOX17*, *GATA4*, *FOXA2*, and *HNF4A*) (Figure S1B). Roughly, ∼75% and ∼98% of cells on day 3 are SOX17^+^ and GATA6^+^, respectively (Figure S1D). *GATA6* expression persists from day 6 onward, coinciding with the activation of the signature pancreatic lineage marker *PDX1* (Figure S1B). By day 12, *GATA6* is co-expressed with genes associated with endocrine commitment (*NGN3* and *NKX6-1*), with approximately 76% and 88% of the differentiated cells PDX1^+^ or GATA6^+^, respectively (Figures S1B and S1E). The expression of islet hormone genes (*INSULIN*, *GLUCAGON*, and *SOMATOSTATIN*) increases from day 12 (Figure S1B). Importantly, immunofluorescence (IF) staining reveals co-localization of SOX17 and GATA6 in day 3 DE as well as PDX1 and GATA6 in day 12 pancreatic endoderm (PE) (Figure S1C). These data were confirmed in a healthy hiPSC line, FSPS13.B, hereafter designated 13.B (Figures S2A–S2C). Taken together, these findings establish developmental windows where *GATA6* insufficiency can result in the pancreatic hypoplasia observed in human *GATA6* heterozygous patients.

### Generation of *GATA6* Mutant Alleles Using TALENs and Derivation of hiPSCs from Two Independent *GATA6* Heterozygous Patients

**Figure 1:**
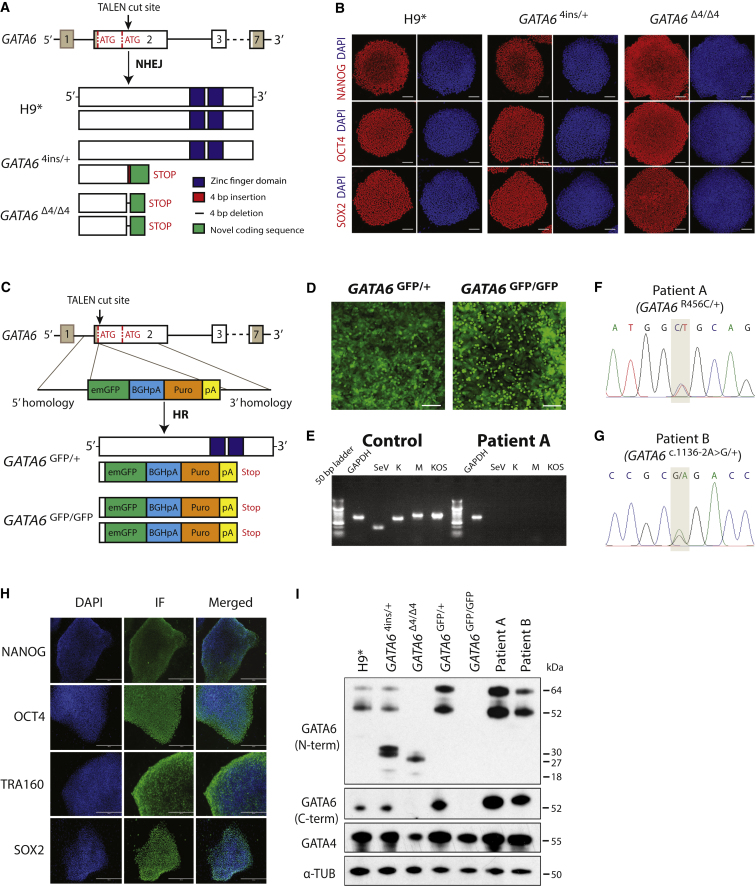
Derivation and Characterization of *GATA6* Mutant Lines (A) Schematic of the *GATA6* locus. Gray shading highlights the 5′ and 3′ untranslated regions. The TALEN cut site lies downstream of the second start ATG in exon 2. Successful gene editing in H9 cells yielded a *GATA6* heterozygous line containing a 4-bp insertion (*GATA6*^4ins/+^) and a homozygous line with an identical 4-bp deletion on each chromosome (*GATA6*^Δ4/Δ4^). Each mutation results in the addition of novel coding sequence (green) and a premature stop. H9^∗^ cells were subjected to gene editing and selection, but have no mutation in *GATA6*. (B) OCT4, SOX2, and NANOG immunofluorescence in H9^∗^, *GATA6*^4ins/+^, and *GATA6*^Δ4/Δ4^ lines confirms pluripotency in gene-edited clones. Scale bars, 100 μm. (C) A second TALEN cut site downstream of the first ATG in exon 2 of *GATA6* is depicted. Cartoon schematic of the “knockin” vector that introduces an emerald GFP (emGFP) reporter in-frame and a puromycin-resistance cassette. Successful homologous recombination resulted in both heterozygous (*GATA6*^GFP/+^) and homozygous (*GATA6*^GFP/GFP^) mutant cells (D) Immunofluorescence showing emGFP-expressing heterozygous *GATA6*^GFP/+^ and homozygous *GATA6*^GFP/GFP^ mutant cells on day 3 of differentiation. Scale bars, 100 μm. (E) PCR showing loss of transgenes in a patient A mutant hiPSC line, clone 1, compared with positive controls. Data are representative of three independent clones derived from either patient A or patient B. (F and G) Genotype confirmation by Sanger sequencing of two *GATA6* patient-derived hiPSC lines: (F) patient A, *GATA6*^R465C/+^, and (G) patient B, *GATA6*^c.1136–2A>G/+^. (H) Immunofluorescence confirming the successful reprogramming and pluripotency of one patient A-derived (*GATA6*^R465C/+^) mutant line. Scale bars, 200 μm. Images are representative of three independent clones derived from either patient A or patient B (*GATA6*^c.1136–2A>G/+^). (I) Western blot analysis of GATA6 and GATA4 protein levels in undifferentiated H9^∗^, *GATA6*^4ins/+^, *GATA6*^Δ4/Δ4^, *GATA6*^GFP/+^, and *GATA6*^GFP/GFP^ mutant cells, as well as the two patient-derived mutant lines: patient A, *GATA6*^R465C/+^, and patient B, *GATA6*^c.1136–2A>G/+^. α-tubulin was used as a loading control. Long and short isoforms of wild-type GATA6 are 60 and 45 kDa, respectively; the partial protein products for *GATA6*^4ins/+^ are 30 and 18 kDa for the long and short isoforms, respectively; the partial protein products for *GATA6*^Δ4/Δ4^ are 27 and 15 kDa for the long and short isoforms, respectively. No GATA6 protein was present for the *GATA6*^GFP/GFP^ mutant.

To pinpoint the precise role of *GATA6* in the human pancreatic lineage, we performed genome-editing in hPSCs as well as isolated patient-derived hiPSCs to generate a panel of *GATA6* mutant alleles to model pancreatic agenesis *in vitro*. The human *GATA6* gene is transcribed from two distinct promoter regions, contains two initiation codons in exon 2 (a second at Met147), and consequently encodes two GATA6 protein isoforms, with masses of 60 and 45 kDa, respectively (Figure 1A; Brewer et al., 1999). We initially targeted both H9 and 13.B at a TALEN cut site immediately 3′ of the first ATG in *GATA6*. Despite the introduction of frameshift mutations that result in premature stop codons, translation still initiated at the second ATG, producing the shorter GATA6 isoform at wild-type levels (data not shown). Thus, in subsequent experiments, we targeted *GATA6* at a second TALEN cut site 3′ of the second ATG (Figure 1A) and successfully recovered *GATA6* heterozygous (*GATA6*^c.618_619insTGCA/+^, hereafter *GATA6*^4ins/+^) and homozygous (*GATA6*^c.611_614delACCT/c.611_614delACCT^, hereafter *GATA6*^Δ4/Δ4^) mutations in H9 cells. We generated similar insertion or deletion alleles, both heterozygous (*GATA6*^c.del614_627TGCAGGGGTCGGGC/+^, hereafter *GATA6*^Δ14/+^) and compound heterozygous (*GATA6*^c.del614_627TGCAGGGGTCGGGC/c.del613_623CTGCAGGGGTC^, hereafter *GATA6*^Δ14/Δ11^), in 13.B. In parallel, we inserted an emerald GFP (emGFP) reporter in-frame with the first *GATA6* ATG via homologous recombination (*GATA6*^GFP/+^) in H9 cells (Figures 1C and 1D) and 13.B cells. An H9 *GATA6*^GFP^ homozygous clone was also recovered (*GATA6*^GFP/GFP^) (Figure 1C). Unfortunately, no 13.B *GATA6*^GFP^ homozygous clone was recovered despite numerous attempts. Control TALEN-targeted lines that harbor no mutations in *GATA6* (designated H9^∗^ or 13.B^∗^) served as wild-type, isogenic positive controls for differentiation experiments involving genome-edited hPSCs. Last, we obtained fibroblasts from two *GATA6* heterozygous patients, whose mutations were previously described (De Franco et al., 2013, Yu et al., 2014). Patient A contains a missense mutation (c.1366C>T) at a highly conserved amino acid within the second zinc-finger DNA-binding domain (Arg456Cys) (Figure 1F), while patient B contains a splice acceptor mutation in exon 3 (*GATA6*^c.1136–2A>G/+^) (Figure 1G). Three independent hiPSC clones were isolated for each patient line. All hESC and hiPSC lines were found to have a normal karyotype by multiplex fluorescence *in situ* hybridization (see Supplemental Experimental Procedures) and assayed by immunohistochemistry to confirm their pluripotency (Figures 1B and 1H and data not shown). hiPSC lines were also monitored for absence of the Sendai virus (Figure 1E).

### Differentiation of *GATA6* Mutant hPSC Lines into the Definitive Endoderm Lineage

Mutant lines were next differentiated to the DE stage and GATA6 protein levels determined by western blot using anti-N- and anti-C-terminal GATA6 antibodies (Figure 1I). In H9^∗^ DE cells, both GATA6 isoforms are detected by the N-terminal antibody, whereas the C-terminal antibody predominantly recognizes the short isoform (Figure 1I) (Brewer et al., 1999). *GATA6*^4ins/+^ and *GATA6*^Δ4/Δ4^ contain frameshift mutations that result in truncated partial protein products predicted to contain 205 and 203 N-terminal amino acids, respectively, of the longer GATA6 isoform as well as additional novel C-terminal sequences (Figures 1A and 1I) that terminate before the two C-terminal zinc-finger DNA-binding domains. The insertion of the GFP reporter and puromycin-resistance cassettes in *GATA6* exon 2 generates a loss-of-function allele, since neither wild-type GATA6 isoform nor novel partial protein products were observed in *GATA6*^GFP/GFP^ knockin H9 cells (Figures 1C and 1I).

**Figure 2:**
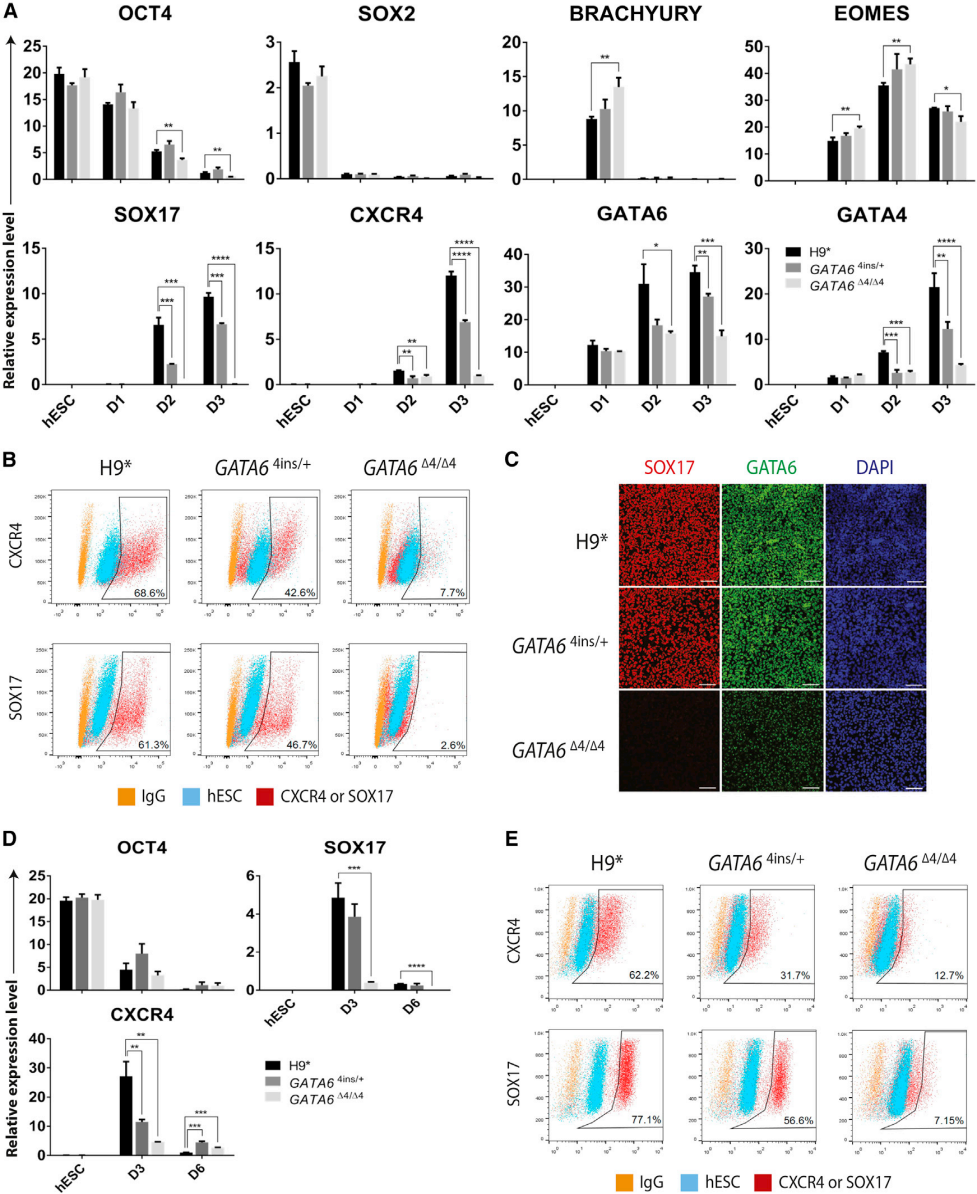
*GATA6*^4ins/+^ and *GATA6*^Δ4/Δ4^ Mutant hESC Lines Display Impaired DE Formation (A) Expression of pluripotency (*OCT4*, *SOX2*), primitive streak (*BRACHYURY*), mesendoderm (*EOMES*), and definitive endoderm (*CXCR4*, *SOX17*, *GATA4*) markers, as well as *GATA6* itself, in H9^∗^ and H9-derived *GATA6*^4ins/+^ and *GATA6*^Δ4/Δ4^ mutant cells over 3 days of differentiation (Figure S1A). (B) Differentiation efficiency measured by FACS analysis of CXCR4 and SOX17 at day 3 DE in H9^∗^ and H9-derived *GATA6*^4ins/+^ and *GATA6*^Δ4/Δ4^ mutant cells. (C) Immunofluorescence analyses for the key DE markers GATA6 with SOX17 in H9^∗^ and H9-derived *GATA6*^4ins/+^ and *GATA6*^Δ4/Δ4^ mutant cells. DAPI, 4′,6-diamidino-2-phenylindole. Scale bars, 100 μm. (D) Expression of pluripotency (*OCT4*) and definitive endoderm (*SOX17*, *CXCR4*) markers in H9^∗^ and H9-derived *GATA6*^4ins/+^ and *GATA6*^Δ4/Δ4^ mutant cells on days 3 and 6 of differentiation with the STEMdiff pancreatic progenitor kit. (E) Differentiation efficiency measured by FACS analysis of CXCR4 and SOX17 at day 3 DE in H9^∗^ and H9-derived *GATA6*^4ins/+^ and *GATA6*^Δ4/Δ4^ mutant cells differentiated using the STEMdiff pancreatic progenitor kit. (A and D) Error bars represent the SE of three independent experiments. ^∗^p < 0.05, ^∗∗^p < 0.01, ^∗∗∗^p < 0.001, ^∗∗∗∗^p < 0.0001. (B and E) Undifferentiated hESCs stained with the respective primary and secondary antibodies and secondary antibody only (IgG) were both used as controls. Gates were set according to an hESC control.

Using the H9-derived *GATA6*^4ins/+^ and *GATA6*^Δ4/Δ4^ lines, we next asked whether reduced levels of GATA6 have an impact on early mesendoderm (corresponding to days 1 and 2) to DE (day 3) differentiation. qRT-PCR analyses show that in H9^∗^, *GATA6*^4ins/+^, and *GATA6*^Δ4/Δ4^ cells, the levels of the pluripotency markers *OCT4* and *SOX2* were comparable in undifferentiated cells and expectedly declined during differentiation (Figure 2A). The expression of primitive streak (*BRACHYURY*) and mesendoderm (*EOMESODERMIN* [*EOMES*]) markers was also relatively unchanged across the control H9^∗^ and *GATA6* mutant lines, suggesting that early mesendoderm formation was not affected by either single or biallelic loss of *GATA6* (Figure 2A). Key DE markers *SOX17* and *CXCR4* were, however, modestly downregulated beginning on day 2 in *GATA6*^4ins/+^ cells (Figure 2A), and on day 3, *GATA6*^4ins/+^ differentiations yielded roughly 25% fewer SOX17^+^ cells by fluorescence-activated cell sorting (FACS) and IF compared with wild-type H9^∗^ (Figures 2B and 2C). This heterozygous effect on *SOX17* transcription was also observed to varying degrees in H9-*GATA6*^GFP/+^ and 13.B-derived *GATA6*^Δ14/+^ as well as in patients A (*GATA6*^R456C/+^) and B (*GATA6*^c.1136–2A>G/+^) (Figures S2D–S2F). Interestingly, this heterozygous effect was not observed in 13.B-derived *GATA6*^GFP/+^ (Figure S2E). Further depleting GATA6 with homozygous (*GATA6*^Δ4/Δ4^ or *GATA6*^GFP/GFP^) or compound heterozygous (13.B-*GATA6*^Δ14/Δ11^) allelic combinations dramatically affects DE formation, yielding ∼3% SOX17^+^ cells on day 3 (Figure 2B and data not shown). We further validated these results using the commercially available STEMdiff pancreatic progenitor kit from STEMCELL Technologies. Using this differentiation platform, H9^∗^, *GATA6*^4ins/+^, and *GATA6*^Δ4/Δ4^ formed DE at efficiencies indistinguishable from the results obtained with the protocol outlined in Figure 1A (cf. Figures 2A and 2B with 2D and 2E). Taken together, these findings show that diminished levels of GATA6 compromise early DE formation, and complete loss of GATA6 significantly perturbs the gene regulatory network (GRN) governing human DE specification.

### Establishing the GATA6 Gene Regulatory Network

**Figure 3:**
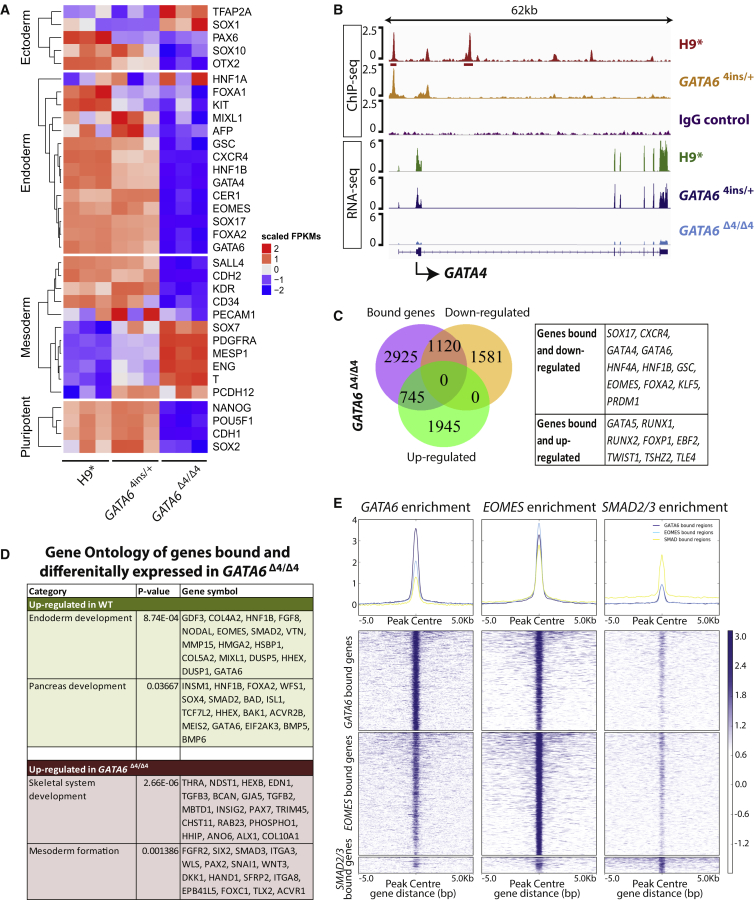
*GATA6* Is a Key Regulator of the DE Transcriptional Network (A) Heatmap illustrating differential gene expression of key germ layer markers via RNA-seq between H9^∗^ cells and H9-derived *GATA6*^4ins/+^ and *GATA6*^Δ4/Δ4^ mutant cells at the DE stage. n = 3 biological replicates for each cell line. (B) ChIP-seq binding profiles of H9^∗^ and *GATA6*^4ins/+^ showing *GATA6* enrichment near *GATA4*, and *GATA4* expression by RNA-seq in H9^∗^ and H9-derived *GATA6*^4ins/+^ and *GATA6*^Δ4/Δ4^ mutant cells at the DE stage. The input control profile (IgG control) is included for comparison. The ChIP-seq binding profile is derived from merging two biological replicates. (C) Venn diagram indicating the overlap of GATA6-bound genes from ChIP-seq at the DE stage with downregulated or upregulated genes in H9-derived *GATA6*^Δ4/Δ4^ mutant cells compared with H9^∗^ cells by RNA-seq. Key bound genes up- or downregulated are indicated in the table. (D) Enriched gene ontology showing developmental pathways from direct target genes differentially expressed between H9^∗^ and H9-derived *GATA6*^Δ4/Δ4^ mutant cells derived from BETA analysis. (E) Density heatmaps of *GATA6*-binding peak intensity at DE indicating direct overlap with known endodermal regulators, including *SMAD2/3* and *EOMES*, within a 5-kb window centered at the transcription start site.

To establish comprehensively how *GATA6* mutations alter the DE transcriptional network, we performed RNA sequencing (RNA-seq) for H9^∗^, *GATA6*^4ins/+^ and *GATA6*^Δ4/Δ4^, and patient A cells on day 3 of differentiation. Comparative analyses revealed 7,472 genes that are differentially expressed (adjusted p ≤ 0.01; fold change ≥2) between H9^∗^ and *GATA6*^Δ4/Δ4^, 2,898 genes between H9^∗^ and *GATA6*^4ins/+^, and 6,977 genes between H9^∗^ and hiPSC clones 1 to 3 from patient A (Table S1). We observed that, consistent with our qRT-PCR data in Figure 2, *GATA6*^Δ4/Δ4^ mutant cells show significantly decreased expression of cardinal endoderm markers (e.g., *SOX17*, *CXCR4*, *HNF1B*, and *FOXA2*) (Figure 3A). Similar results were observed when wild-type H9^∗^ was compared with *GATA6*^4ins/+^ and hiPSC clones 1 to 3 from patient A (Figures 3A and S3A).

We also performed GATA6 chromatin immunoprecipitation followed by high-throughput sequencing (ChIP-seq) on H9^∗^ and *GATA6*^4ins/+^ cells at the DE stage. This analysis yielded 12,098 peaks (irreproducible discovery rate ≤0.05; median peak length = 417 bp) that are associated with 10,669 genes, 4,790 of which are protein coding (Table S1). Interestingly, we observe that GATA6 binding is enriched at the *GATA4* locus in H9 cells, suggesting that GATA6 directly regulates *GATA4* during DE specification (Figure 3B). Both qRT-PCR and RNA-seq show dose-dependent effects of GATA6 on *GATA4* expression levels in *GATA6*^4ins/+^ and *GATA6*^Δ4/Δ4^ mutant cells (Figures 2A, 3A, and 3B).

We next compared our RNA-seq and ChIP-seq datasets to identify those genes bound and regulated by GATA6. This analysis revealed 1,120 protein-coding genes that are bound by GATA6 in wild-type H9^∗^ but are downregulated in *GATA6*^Δ4/Δ4^ mutant cells, including pancreatic progenitor genes such as *HNF1B* and *HNF4A* (Figure 3C). In contrast, 745 genes are bound by GATA6 in H9^∗^ and upregulated in *GATA6*^Δ4/Δ4^. Similar overlaps were performed for *GATA6*^4ins/+^ and patient A day 3 RNA-seq samples, yielding 337 and 607 GATA6-bound and downregulated genes, and 254 and 616 GATA6-bound and upregulated genes, respectively (Figures S3B and S3C). At the intersection of these comparisons are 143 commonly downregulated and 104 upregulated genes among *GATA6*^Δ4/Δ4^, *GATA6*^4ins/+^, and patient A samples (Figures S3D and S3E). Key endoderm markers *CXCR4*, *SOX17*, *GATA4*, *HNF1B*, and *HNF4A* were among the 143 genes commonly downregulated (Figure S3D).

To infer genes that are directly targeted and regulated by GATA6, we performed binding and expression target analysis (BETA) to integrate our H9 ChIP-seq dataset with differential gene expression data from *GATA6*^Δ4/Δ4^, *GATA6*^4ins/+^ and patient A (Wang et al., 2013). Targets predicted by BETA were then subjected to gene ontology analyses using the DAVID tool (Huang da et al., 2009a, Huang da et al., 2009b). We found that endoderm development is consistently upregulated in H9^∗^ compared with *GATA6*^Δ4/Δ4^ (Figure 3D), *GATA6*^4ins/+^ (Figure S3F), and patient A (Figure S3G) mutant cells. In addition, mesoderm development is consistently upregulated in *GATA6*^Δ4/Δ4^ (Figure 3D), *GATA6*^4ins/+^ (Figure S3F), and patient A (Figure S3G) mutant cells compared with H9^∗^. Motif analyses generated by BETA confirm that the GATA recognition motif is highly enriched in both “up” and “down” target genes (Figure S3H). We were unable to conclude from the BETA whether GATA6 has activating or repressive functions, or both, as the data were not significant. Thus, we propose that the most parsimonious explanation for the upregulation of mesodermal markers is aberrant differentiation. In the absence of *GATA6*, differentiation into the DE lineage is blocked, but differentiating cells remain bathed in high levels of two potent mesoderm inducers (Activin and BMP4) (Cho et al., 2012). Taken together, these results show that *GATA6* is indispensable in driving the development of the human DE.

We previously established that the T-box transcription factor EOMES interacts with the Activin/Nodal effector proteins SMAD2/3 to deploy the GRN that directs DE formation. We thus sought to establish how GATA6 integrates into the SMAD2/3/EOMES signaling network by comparing our GATA6 day 3 ChIP-seq data with previously published SMAD2/3 and EOMES ChIP-seq (Brown et al., 2011, Teo et al., 2011). Remapping of the data resulted in 16,303, 20,089, and 2,613 peaks for GATA6, EOMES, and SMAD2/3, respectively. Of the 16,303 GATA6 ChIP-seq peaks, 950/2,613 (36.4%) overlap with SMAD2/3, and 8,126/20,089 (40.5%) overlap with EOMES in DE cells, with 858 common to all three datasets (Figure 3E, Table S1). In the EOMES/GATA6/SMAD2/3 intersection, we find almost all of the telltale endodermal regulator genes, including *SOX17*, *EOMES*, *LHX1*, *MIXL1*, *FOXA2*, *HNF1B*, and C*XCR4*. These data therefore place GATA6 centrally within the core nuclear transcriptional machinery that governs the acquisition of DE fate.

### GATA6 Deficiency Impairs Pancreatic Lineage Commitment

**Figure 4:**
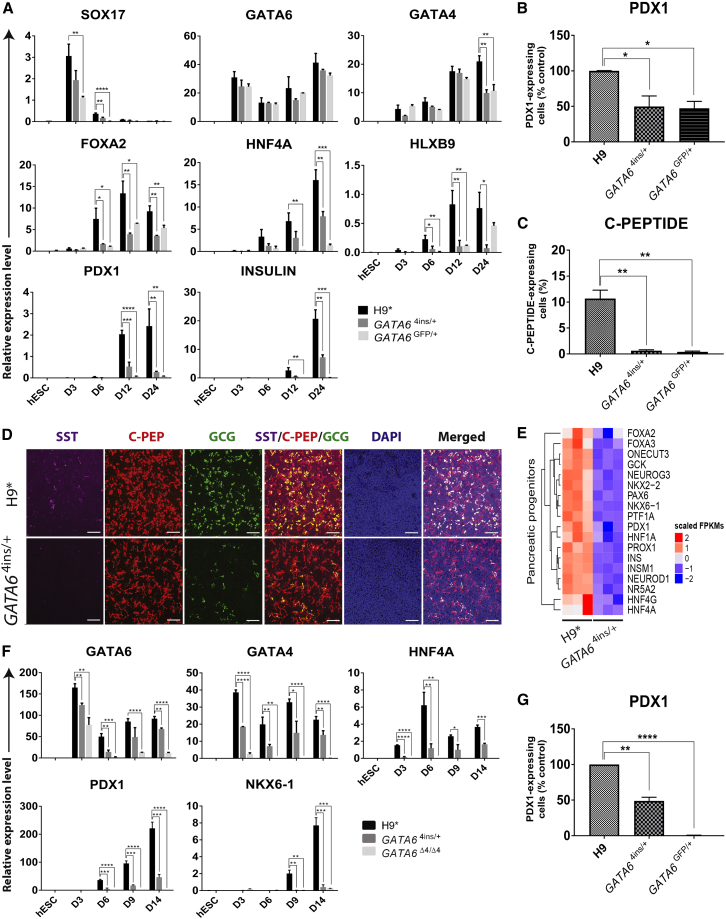
Decreased Levels of GATA6 at the DE Stage Influence Downstream Pancreatic Differentiation (A) Expression of DE (*SOX17*, *GATA6*, *GATA4*, and *FOXA2*), pancreatic (*HNF4A*, *HLXB9*, and *PDX1*), and endocrine (*INSULIN*) marker genes in H9^∗^ and H9-derived *GATA6*^4ins/+^ and *GATA6*^Δ4/Δ4^ mutant cells at the four key stages of the 24-day pancreatic differentiation protocol (Figure S1A). (B) Percentage of PDX1-positive cells in H9-derived *GATA6*^4ins/+^ and *GATA6*^GFP/+^ lines on day 12 shown relative to H9^∗^ (100%) as measured by FACS. (C) Percentage of C-PEPTIDE-positive cells in H9^∗^ and H9-derived *GATA6*^4ins/+^ and *GATA6*^GFP/+^ mutant lines at the EP stage (day 24) as measured by FACS. (D) Immunofluorescence analyses for the key PE markers SOMATOSTATIN (SST), C-PEPTIDE (C-PEP), and GLUCAGON (GCG) in H9^∗^ and H9-derived *GATA6*^4ins/+^ cells at the EP stage (day 24). DAPI, 4′,6-diamidino-2-phenylindole dihydrochloride. Scale bars, 100 μm. (E) Heatmap illustrating differential gene expression of key pancreatic progenitor markers via RNA-seq between H9^∗^cells and H9-derived *GATA6*^4ins/+^ mutant cells at the PE stage. n = 3 biological replicates for each cell line. (F) Expression of DE (*GATA6* and *GATA4*) and pancreatic (*HNF4A*, *PDX1*, and *NKX6-1*) marker genes in H9^∗^ and H9-derived *GATA6*^4ins/+^ and *GATA6*^Δ4/Δ4^ mutant cells at key stages of the differentiation protocol using the STEMdiff pancreatic progenitor kit. (G) Percentage of PDX1-positive cells in *GATA6*^4ins/+^ and *GATA6*^GFP/+^ lines on day 12 shown relative to H9^∗^ (100%) as measured by FACS in cells differentiated using the STEMdiff pancreatic progenitor kit. (A–C, F, and G) Error bars represent the SE of three independent experiments. ^∗^p < 0.05, ^∗∗^p < 0.01, ^∗∗∗^p < 0.001, ^∗∗∗∗^p < 0.0001.

We further analyzed the effects of *GATA6* heterozygous mutations on pancreatic lineage commitment at the PE (day 12) and endocrine progenitor (EP) (day 24) stages (Figure S1A). Key pancreatic markers such as *HNF4A*, *HLXB9*, *PDX1*, and *INSULIN* are significantly downregulated in *GATA6*^4ins/+^ and *GATA6*^GFP/+^ mutant cells at both stages, with one exception: *HLXB9* levels in *GATA6*^GFP/+^ are no different from those in H9^∗^ on day 24 (Figure 4A). *HNF4A*, *PDX1*, and *INSULIN* were also significantly decreased in 13.B-*GATA6*^Δ14/+^ and 13.B-*GATA6*^GFP/+^ and in patient A and B mutant cells on days 12 and 24 (Figures S4A and S4B). FACS analysis for PDX1 on day 12 reveals an approximately 50% decrease in the number of PDX1-positive *GATA6*^4ins/+^ and *GATA6*^GFP/+^ cells and 13.B-*GATA6*^GFP/+^ cells (Figures 4B and S4C). 13.B-*GATA6*^Δ14/+^, patient A, and patient B cell lines exhibit an approximately 80%–90% decrease in PDX1 (Figures S4C and S4D). At the EP stage, all *GATA6* heterozygous mutant cell lines share a common phenotype, with a strong decrease in the number of C-PEPTIDE^+^ cells (Figure 4C, S4C, and S4D). Immunostaining on H9^∗^ and *GATA6*^4ins/+^ cells confirms the diminished number of SOMATOSTATIN-, C-PEPTIDE-, and GLUCAGON-positive cells in *GATA6*^4ins/+^ cells (Figure 4D).

We also performed RNA-seq at the PE stage (day 12) for H9^∗^, *GATA6*^4ins/+^, and patient A cells. H9^∗^ RNA-seq largely reproduced a previous dataset generated using both H9 and the same differentiation protocol (Spearman's rank correlation coefficient, ρ = 0.77 for *in vitro* multipotent pancreatic progenitor cells [MPCs] and ρ = 0.59 for *in vivo* MPCs isolated from Carnegie stage 16–18 human embryos, p < 2.2 × 10^−16^) (Cebola et al., 2015). Between H9^∗^ and *GATA6*^4ins/+^ (Table S1), 1,423 genes were differentially expressed, and between H9^∗^ and patient A, 6,093 were differentially expressed (Table S1). We observed that, consistent with qRT-PCR and FACS results, *GATA6*^4ins/+^ and patient A gene expression quantified by RNA-seq in mutant cells displays a decreased pancreatic signature (Figures 4E and S4E).

Finally, the above results were independently confirmed with H9^∗^, *GATA6*^4ins/+^, and *GATA6*^Δ4/Δ4^ cells using the STEMdiff pancreatic progenitor kit: *GATA6*^4ins/+^ cells show decreased *PDX1* and *NKX6-1* expression beginning on day 9, yielding ∼50% fewer PDX1^+^ pancreatic progenitors on day 12 (Figures 4F and 4G). Collectively, these *in vitro* findings strongly argue that decreased levels of GATA6 first influence the formation of DE, and predict that with fewer DE cells at the time of allocation to the pancreatic lineage *in vivo*, hypoplasia emerges.

## Discussion

Deriving iPSC lines and the ability to rapidly engineer mutations in hPSCs have made human disease modeling *in vitro* commonplace. In the case of the well-characterized set of genes known to control mammalian pancreatic development, it is the expectation based on strong evolutionary conservation that phenotypes observed in knockout mouse models will be reproduced in human and *in vitro*. Indeed, the matching of human and mouse pancreatic and extrapancreatic phenotypes is seen for many recessive loss-of-function mutations in key pancreatic developmental transcription factors, e.g., PDX1, *PTF1A*, *RFX6*, *NEUROD1*, *NGN3*, and *NKX2-2* (Ahlgren et al., 1996, Flanagan et al., 2014, Jonsson et al., 1994, Offield et al., 1996, Rubio-Cabezas et al., 2010, Rubio-Cabezas et al., 2011, Schwitzgebel et al., 2003, Sellick et al., 2004, Smith et al., 2010, Stoffers et al., 1997). Such consistency in phenotype is, however, not observed with *Gata6*. In mice, only the simultaneous inactivation of *Gata6* and *Gata4* results in pancreatic agenesis (Carrasco et al., 2012, Xuan et al., 2012), whereas in humans *de novo* heterozygous mutations in *GATA6* underlie a similar pathology (De Franco et al., 2013, Lango Allen et al., 2011). However, *GATA6* heterozygous phenotypes range from total pancreatic agenesis to isolated diabetes in young adulthood. This phenotypic diversity partly explains the difficulties in precisely modeling *GATA6* haploinsufficiency *in vitro* across laboratories and across differentiation platforms, as evidenced by comparing our present findings with two recent reports from Shi et al. (2017) and Tiyaboonchai et al. (2017).

Here, we find a modest reduction (∼25%) in the production of DE after 3 days of directed differentiation irrespective of whether the *GATA6* heterozygous line was patient derived or generated by gene editing in hPSCs. These findings are consistent with *GATA6* expression in the DE, but contrast with the results of Shi et al. (2017) and Tiyaboonchai et al. (2017). These authors did not observe decreased DE formation using assorted *GATA6* heterozygous hPSC lines. One potential explanation for these discrepant results is that GATA6 partial protein products, generated, for example, from the *GATA6*^4ins/+^ allele in H9 cells, act in our hands in a dominant negative manner, further suppressing *GATA6* levels and compromising normal DE formation (Figure 1I). The partial protein products encoded by the *GATA6*^4ins/+^ locus are predicted to retain a long stretch of the N-terminal GATA6 transactivation domain but lack the zinc-finger DNA-binding domain and nuclear localization signal. As they are able neither to bind DNA nor to heterodimerize with GATA4 (Charron et al., 1999, Maeda et al., 2005), the biochemical mechanism by which such partial protein products interfere with *GATA6* transcription or function is entirely unclear. Moreover, Tiyaboonchai et al. (2017) and Shi et al. (2017) also observe partial protein products in their *GATA6* heterozygous hPSC lines, but do not observe effects during DE differentiation. The most significant evidence against dominant interference and in favor of a simple dosage effect comes from the fact that patient A and B iPSCs, whose mutations do not result in partial protein products (Figure 1I), also show decreased DE formation on day 3. Alternatively, because each group employed different hESC and iPSC lines, the specter of well-known line-to-line variations in differentiation efficiency could explain the results from the different laboratories (Cahan and Daley, 2013, Ortmann and Vallier, 2017).

Despite these differences among the *GATA6* heterozygous phenotypes at the DE stage, complete loss of GATA6 was found by Tiyaboonchai et al., 2017, Shi et al., 2017, and us (Figure 2), as well as more recently by Liao et al. (2018) with short hairpin RNA targeting *GATA6* in H1 cells, to unequivocally impair DE formation, a result highlighting not only the requirement for wild-type *GATA6* gene dosage for robust DE specification in humans but also the dramatic species-specific differences between mice and humans. Importantly, our genome-wide studies place GATA6 among the core transcriptional machinery that controls DE formation. We previously reported that the pluripotency factors OCT4, SOX2, and NANOG bind cooperatively and control the expression of the T-box transcription factor gene *EOMES* (Teo et al., 2011). Upon activation, EOMES, jointly with SMAD2/3, the intracellular effectors of ACTIVIN/NODAL signaling, deploys a large part of the transcriptional network governing DE formation. Here, we find 858 genes that are bound within 5 kb of the transcription start site by EOMES, SMAD2/3, and GATA6. Importantly, such cooperation has not been evidenced in mouse development, suggesting major divergences between species in the molecular mechanisms controlling germ-layer specification. Considering the importance of *GATA6* in specification of extraembryonic endoderm, this divergence in signaling pathways could result in the rewiring of downstream transcriptional networks with major consequences on the subsequent specification of DE.

With extended differentiation to the PE stage (day 12), we observe significantly decreased numbers of PDX1^+^ cells—between 50% and 90% fewer compared with wild-type depending on the *GATA6* heterozygous line. This result is consistent with *GATA6* expression in human pancreatic progenitors (Figure S1C) and with GATA6 directly regulating *PDX1* transcription (Carrasco et al., 2012, Xuan et al., 2012) and also suggests that GATA6 plays a dual role in both early DE formation and allocation to the pancreatic lineage. The diminished numbers of *GATA6* heterozygous PDX1^+^ progenitors that emerge at the PE stage ultimately differentiate into ≤10% C-PEPTIDE^+^ cells by the EP stage (day 24), across all cell types and across all alleles.

It is tempting to consider that the variation in clinical phenotype and the early phenotype in DE formation *in vitro* might be predominantly attributable to individual genetic backgrounds (Chen et al., 2016, Lek et al., 2016). *GATA4* is an obvious choice for a genetic modifier, given its expression in the DE, genetic interaction with *Gata6* in mice, and the identification of rare *GATA4* heterozygous patients with pancreatic agenesis, as well as our finding that *GATA4* is bound and regulated by GATA6 *in vitro* (Figure 3) (D'Amato et al., 2010, Freyer et al., 2015, Morrisey et al., 1996, Shaw-Smith et al., 2014). Indeed, Shi et al. (2017) elegantly show dosage-dependent effects of *GATA4* alleles on phenotypes associated with *GATA6* heterozygosity during *in vitro* differentiation. Despite reports of considerable phenotypic variation between family members with the same *GATA6* mutation (Bonnefond et al., 2012, De Franco et al., 2013, Yau et al., 2017), in some cases a parent is a mosaic for the phenotype, so the variation between parental and offspring phenotypes can be explained by different mutation load in target tissues (Yau et al., 2017). If the variation in the human phenotype altered significantly with the genetic background, then most cases with severe pancreatic agenesis would likely be born to parents with the same mutation, but a 50% different (protective) genetic background would have a milder phenotype. However, this is not the case, as the vast majority of severe pancreatic agenesis is from *de novo* mutations (De Franco et al., 2013, Lango Allen et al., 2011). This means it is possible, but not likely, that genetic background explains why Shi et al. (2017) engineered, using CRISPR/Cas9, the common *GATA6* agenesis mutation c.1366C>T (p.Arg456Cys) in HUES8 cells—the same allele present in our patient A-derived iPSC line (*GATA6*^*R456C/+*^)—and observed no heterozygous phenotype at the DE or pancreatic progenitor (PDX1^+^) stages, whereas we do, at both the DE stage and beyond.

In addition to line-to-line differentiation efficiencies *in vitro* (Cahan and Daley, 2013, Ortmann and Vallier, 2017), fundamental differences in the differentiation protocols themselves may underlie (or contribute to) the results we report here and those published by Shi et al. (2017) and Tiyaboonchai et al. (2017). For example, the growth factor and small-molecule components as well as medium formulations differ substantially for the first 3 days of DE differentiation among the three studies. Furthermore, our differentiation protocol relies on culture media devoid of serum or complex extracellular matrices such as Matrigel. Thus, the minimalist approach of our system could exacerbate the *GATA6* phenotype, revealing a function for this gene that is otherwise masked. This possibility highlights the importance of culture conditions to study gene function in hPSCs and during their differentiation. Tiyaboonchai et al. (2017) additionally show that a *GATA6* heterozygous iPSC line derived from an agenesis patient unexpectedly produced β-like cells *in vitro*. Simply reducing the concentration of retinoic acid 80-fold led to statistically significantly fewer PDX1^+^ cells compared with a wild-type iPSC line that showed negligible sensitivity to the same culture regime. Indeed, current hPSC pancreatic differentiation protocols have been highly tailored and refined, providing redundant and reinforcing signals that perhaps reconfigure underlying GRNs and bypass certain *in vivo* genetic requirements. Moreover, it must be acknowledged that adherent differentiation fails to achieve the 3D complexity of human endoderm formation *in vivo*. Thus, studies of early pancreatic lineage commitment would greatly benefit from universal protocols standardized intra- and inter-laboratory in an effort to minimize line-to-line and protocol-to-protocol differences.

## Experimental Procedures

### Human Pluripotent Stem Cell Culture and Pancreatic Differentiation

hESCs (H9 [WA09 from www.wicell.org]), hiPSCs (FSPS13.B derived in-house from human fibroblasts [http://www.hipsci.org/lines/#/lines/HPSI0813i-fpdm_2]), and *GATA6* patient-derived iPSCs, from patients A and B, were routinely cultured under feeder-free conditions on vitronectin-coated (STEMCELL Technologies #07180) tissue culture plates (Corning) with Essential 8 Medium (Life Technologies #A1517001). All tissue culture was carried out in 5% CO_2_ at 37°C. Pancreatic differentiation was carried out as previously described (Cho et al., 2012), with modifications described in Supplemental Experimental Procedures.

### *GATA6* Patient Samples

The generation of *GATA6* patient-derived hiPSCs was approved by the Great Ormond Street Hospital and Institute of Child Health Research Ethics Committee (ethics reference: 08/H0713/82), and informed consent was obtained from all patients. Skin punch biopsy samples were collected from patients and all hiPSC lines used were derived and validated by the Cambridge Biomedical Research Center hiPSC Core Facility. Reprogramming of the *GATA6* patient fibroblasts to derive *GATA6* patient iPSCs was done by the hiPSC core facility at the Anne McLaren Laboratory for Regenerative Medicine using Sendai virus reprogramming.

### *GATA6*-Mutant and *GATA6*-emGFP Reporter hPSC Derivation

The construction of TALEN vectors, their introduction into H9 or 13.B cells via electroporation, and the screening of drug-resistant clones are described in detail in the Supplemental Experimental Procedures. Two TALEN pairs were generated, each targeting a different site within *GATA6* exon 2. The first TALEN pair targets a site that is 6 bp downstream of the first *GATA6* start codon. The second targets a site that is 149 bp downstream of the second *GATA6* start codon. Primers used for TALEN construction and screening of genomic DNA are listed in Table S2.

### RNA- and ChIP-Sequencing Analysis of Gene Expression

Library preparation and deep sequencing were performed at the Wellcome Trust Sanger Institute (Hinxton, UK). RNA-seq and ChIP-seq were run on Illumina Hiseq v.3 and v.4, respectively, with read length 75 bp and paired ends, and a library fragment size of 100–1,000 bp using a multiplex strategy. RNA-seq and ChIP-seq samples were run in biological triplicates and duplicates, respectively. Additional details of how RNA-seq and ChIP-seq reads were aligned and analyzed can be found in the Supplemental Experimental Procedures.

## Author Contributions

L.V., N.R.D., C.Y.C., and M.C. designed the methods and experiments. C.Y.C. performed the experiments. C.Y.C., P.M., and S.L.I.J.D performed bioinformatics analyses. I.M. and J.G.-B. performed additional differentiation experiments for the revisions. A.T.H. and M.H.S identified the patients and designed the patient sample collection. M.H.S. and R.E.K. collected the patient samples. C.Y.C. and N.R.D. wrote the first draft. All authors approved the final draft and made modifications to the text.

## Accession Numbers

Primary RNA-seq and ChIP-seq datasets generated here are available at ArrayExpress under accession numbers ArrayExpress: E-MTAB-5958 and E-MTAB-5959, respectively.

## Supplemental Information

Document S1. Supplemental Experimental Procedures, Figures S1–S4 and Tables S2–S4

Table S1. GATA6 RNA- and ChIP-Sequencing Analyses

Document S2. Article plus Supplemental Information
