# Supplementary material for: GATA6 Cooperates with EOMES/SMAD2/3 to Deploy the Gene Regulatory Network Governing Human Definitive Endoderm and Pancreas Formation
Source: Stem Cell Reports. 2019 Jan 8;12(1):57–70. doi: 10.1016/j.stemcr.2018.12.003 (PMC6335596; doi:10.1016/j.stemcr.2018.12.003)
Supplement: Document S1. Supplemental Experimental Procedures, Figures S1–S4 and Tables S2–S4 [file mmc1.pdf]

Stem Cell Reports, Volume *12*

Supplemental Information

GATA6 Cooperates with EOMES/SMAD2/3 to Deploy the Gene Regulatory Network Governing Human Definitive Endoderm and Pancreas Formation

Crystal Y. Chia, Pedro Madrigal, Simon L.I.J. Denil, Iker Martinez, Jose Garcia-Bernardo, Ranna El-Khairi, Mariya Chhatriwala, Maggie H. Shepherd, Andrew T. Hattersley, N. Ray Dunn, and Ludovic Vallier

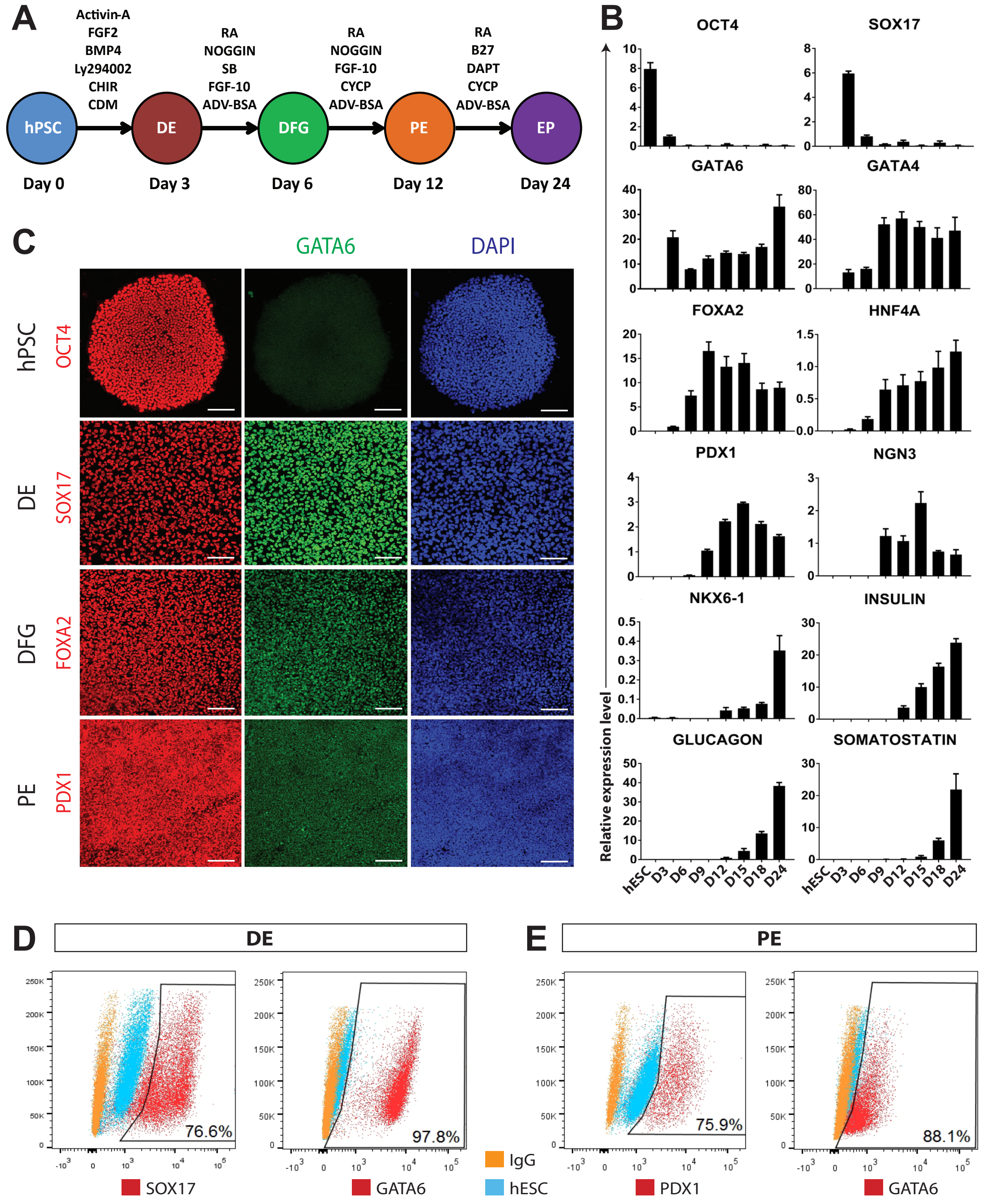
A
Activin-A
FGF2
BMP4
Ly294002
CHIR
CDM
RA
NOGGIN
SB
FGF-10
ADV-BSA
RA
NOGGIN
FGF-10
CYCP
ADV-BSA
RA
B27
DAPT
CYCP
ADV-BSA
hPSC
DE
DFG
PE
EP
Day 0
Day 3
Day 6
Day 12
Day 24
B
OCT4
SOX17
GATA6
GATA4
FOXA2
HNF4A
PDX1
NGN3
NKX6-1
INSULIN
GLUCAGON
SOMATOSTATIN
Relative expression level
hESC D3 D6 D9 D12 D15 D18 D24
C
GATA6
DAPI
hPSC OCT4
DE SOX17
DFG FOXA2
PE PDX1
D
DE
76.6%
97.8%
SOX17
GATA6
IgG
hESC
E
PE
75.9%
88.1%
PDX1
GATA6

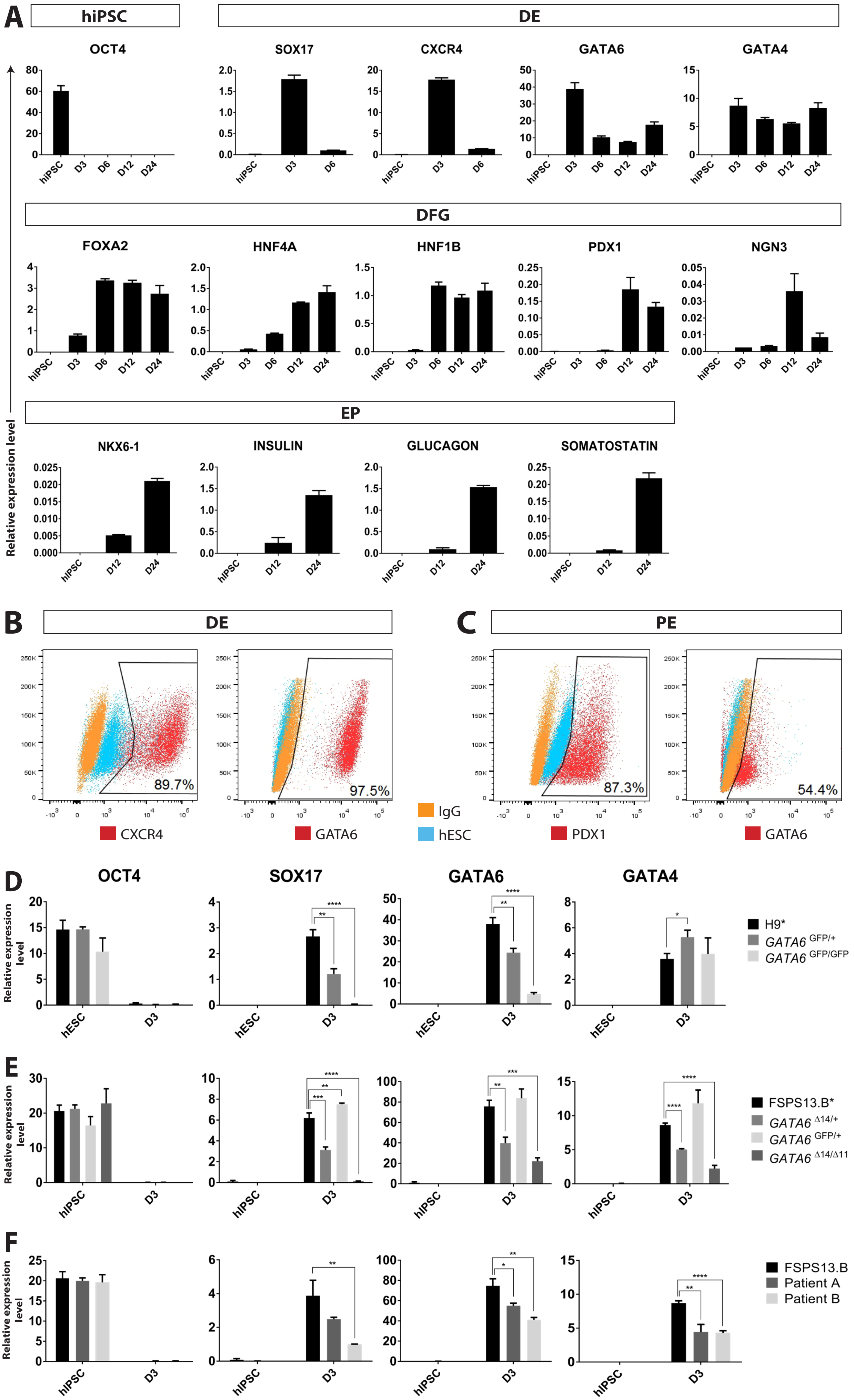

A
hiPSC
DE
DFG
EP
OCT4
SOX17
CXCR4
GATA6
GATA4
FOXA2
HNF4A
HNF1B
PDX1
NGN3
NKX6-1
INSULIN
GLUCAGON
SOMATOSTATIN
Relative expression level
B
DE
C
PE
89.7%
97.5%
87.3%
54.4%
CXCR4
GATA6
IgG
hESC
PDX1
GATA6
D
OCT4
SOX17
GATA6
GATA4
H9*
GATA6 GFP/+
GATA6 GFP/GFP
E
FSPS13.B*
GATA6 Δ14/+
GATA6 GFP/+
GATA6 Δ14/Δ11
F
FSPS13.B
Patient A
Patient B
Relative expression level

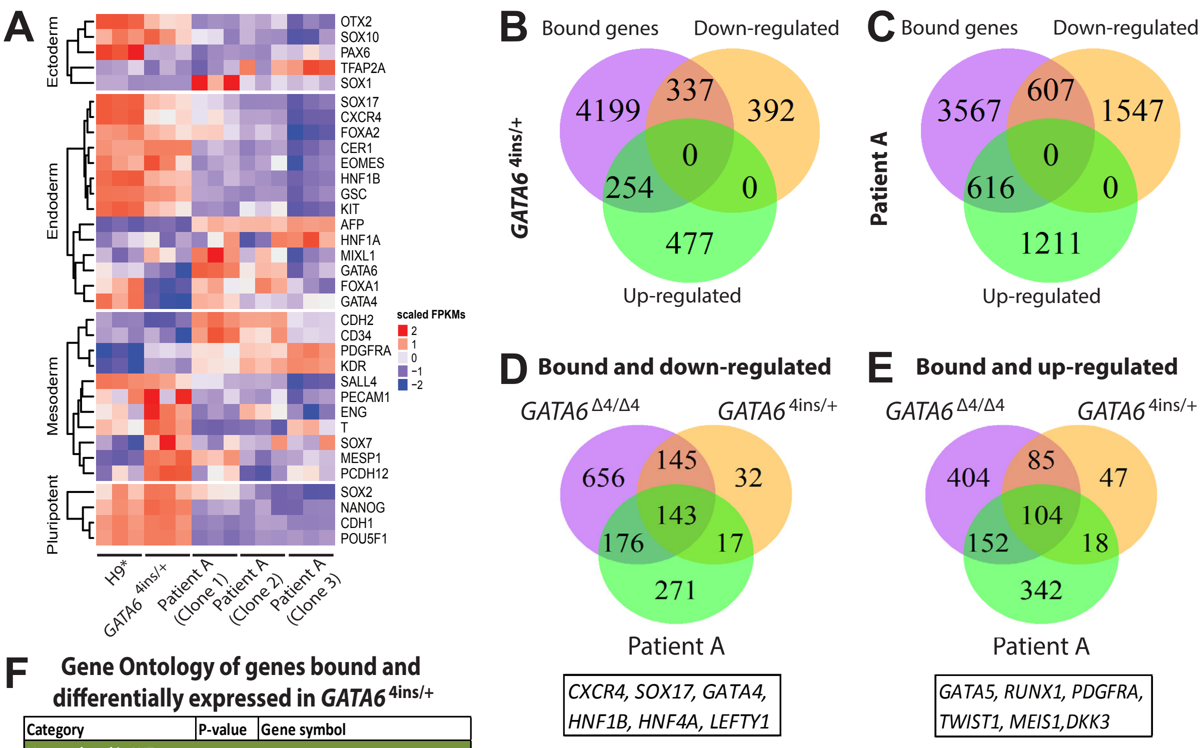

F **Gene Ontology of genes bound and differentially expressed in *GATA6* 4ins/+**

Category	P-value	Gene symbol
**Up-regulated in WT**		
Endoderm development	6.78E-04	GDF3, COL4A2, NOG, HNF1B, NODAL, SMAD2, MMP15, HMGA2, HSBP1, DUSP5, HHEX, DUSP1, GATA6, ITGA7, COL11A1
**Up-regulated in *GATA6* 4ins/+**		
Mesoderm formation	3.31E-04	FGFR2, SIX2, ITGA3, WLS, SMAD1, ITGB1, SNAI1, WNT3, DKK1, HAND1, SFRP2, ITGA8, FOXC1, TLX2

G **Gene Ontology of genes bound and differentially expressed in Patient A**

Category	P-value	Gene symbol
**Up-regulated in WT**		
Endoderm development	0.02678	GDF3, NANOG, HNF1B, ONECUT1, NODAL, EOMES, MMP15, KIF16B, HSBP1, ZFP36L1, HHEX, LHX1, ITGA7, COL6A1
**Up-regulated in Patient A**		
Mesoderm development	0.00119	FGFR2,PAX2, WNT3, OSR1, HAND1, YAP1, TBX3, SMAD4, SIX2, SMAD3, ITGA2, ITGA3, SMAD1, DKK1, ITGA8

H

PART1: UP TARGET GENES

Symbol	DNA BindDom	Species	Pvalue (T Test)	T Score	Logo
GATA5	GATA Domain Family	Homo sapiens	1.06e-222	35.72	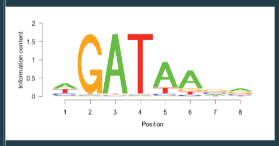
EOMES	Transcription Factor T-Domain				
TAL1	Helix-Loop-Helix Family				
POLR2B	DNA Polymerase-Beta Family				
LMO2	Homeodomain Family				
GATA6	GATA Domain Family				
GATA1	GATA Domain Family				
GATA2	GATA Domain Family				
GATA3	GATA Domain Family				
GATA4	GATA Domain Family				

PART2: DOWN TARGET GENES

Symbol	DNA BindDom	Species	Pvalue (T Test)	T Score	Logo
GATA4	GATA Domain Family	Homo sapiens	9.50e-199	33.97	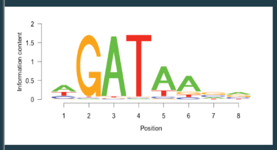
GSC	Homeodomain Family				
TAL1	Helix-Loop-Helix Family				
POLR2B	DNA Polymerase-Beta Family				
LMO2	Homeodomain Family				
GATA1	GATA Domain Family				
GATA6	GATA Domain Family				
GATA3	GATA Domain Family				
GATA2	GATA Domain Family				
GATA5	GATA Domain Family				

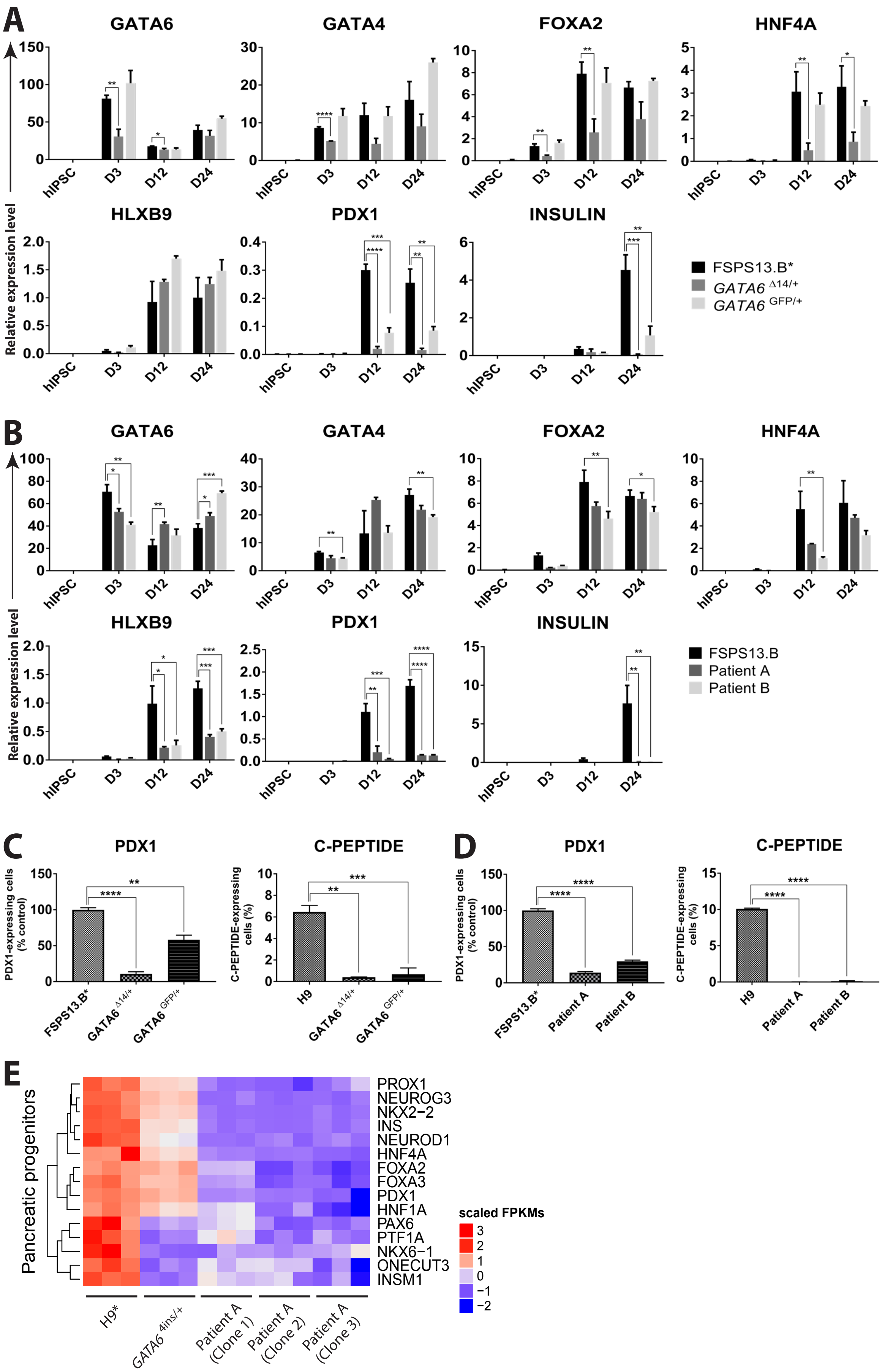
A
GATA6
GATA4
FOXA2
HNF4A
HLXB9
PDX1
INSULIN
Relative expression level
hIPSC
D3
D12
D24
FSPS13.B*
GATA6 Δ14/+
GATA6 GFP/+
B
GATA6
GATA4
FOXA2
HNF4A
HLXB9
PDX1
INSULIN
Relative expression level
FSPS13.B
Patient A
Patient B
C
PDX1
C-PEPTIDE
PDX1-expressing cells (% control)
C-PEPTIDE-expressing cells (%)
FSPS13.B*
GATA6 Δ14/+
GATA6 GFP/+
H9
D
PDX1
C-PEPTIDE
PDX1-expressing cells (% control)
C-PEPTIDE-expressing cells (%)
FSPS13.B*
Patient A
Patient B
H9
E
Pancreatic progenitors
PROX1
NEUROG3
NKX2−2
INS
NEUROD1
HNF4A
FOXA2
FOXA3
PDX1
HNF1A
PAX6
PTF1A
NKX6−1
ONECUT3
INSM1
scaled FPKMs
3
2
1
0
−1
−2
H9*
GATA6 4ins/+
Patient A (Clone 1)
Patient A (Clone 2)
Patient A (Clone 3)

SUPPLEMENTAL FIGURE LEGENDS

Supplemental Figure 1. Directed differentiation of H9 cells into the pancreatic lineage.

(A) Schematic of the 24-day differentiation protocol. DE, definitive endoderm; DFG, dorsal foregut; PE, pancreatic endoderm; EP, endocrine progenitors. The culture medium and supplements indicated are BMP (Bone Morphogenetic Protein 4), the PI3 kinase inhibitor Ly294002, CHIR (the GSK3 inhibitor CHIR99021), CDM (Chemically Defined Medium), Adv-BSA (Advanced Dulbecco's Modified Eagle Medium/Ham's F-12 medium supplemented with BSA and L-glutamine), RA (retinoic acid), SB (the ALK4/5/78 inhibitor SB-431542), FGF2 (Fibroblast Growth Factor 2), FGF10 (Fibroblast Growth Factor 10), CYCP (the Hedgehog inhibitor Cyclopamine-KAAD), B27 supplement, and the NOTCH inhibitor DAPT.

(B) Expression of key marker genes during pancreatic differentiation in H9 cells. $n = 3$ independent experiments for each stage of differentiation.

(C) Immunofluorescence analyses showing co-expression of GATA6 with SOX17, FOXA2, and PDX1. DAPI, 4', 6-diamidino-2-phenylindole dihydrochloride. Scale bars, 100 µm.

(D, E) Differentiation efficiency measured by FACS analysis of SOX17 and GATA6 at day 3 definitive endoderm, and PDX1 and GATA6 at day 12 pancreatic endoderm. Undifferentiated hESC stained with the respective primary and secondary antibodies and secondary antibody only (IgG) were both used as controls. Gates were set according to hESC control.

Supplemental Figure 2. Directed differentiation of FSPS13.B cells into the pancreatic lineage, and mutant hPSC lines and Patients A and B display impaired DE formation.

(A) Expression of key marker genes during pancreatic differentiation in FSPS13.B cells. $n = 3$ independent experiments for each stage of differentiation (**Supp. Fig. 1A**).

(B, C) Differentiation efficiency measured by FACS analysis of CXCR4 and GATA6 at day 3 definitive endoderm, and PDX1 and GATA6 at day 12 pancreatic endoderm. Undifferentiated hESC stained with the respective primary and secondary antibodies and secondary antibody only (IgG) were both used as controls. Gates were set according to hESC control.

(D) Expression of pluripotency (*OCT4*) and definitive endoderm (*SOX17, GATA6* and *GATA4*) markers at day 3 DE in H9* and H9-derived $GATA6^{GFP/+}$ and $GATA6^{GFP/GFP}$ mutant cells.

(E) Expression of pluripotency (*OCT4*) and definitive endoderm (*SOX17, GATA6* and *GATA4*) markers at day 3 DE in FSBS13.B* and FSBS13.B-derived *GATA6*$^{\Delta 14/+}$, *GATA6*$^{GFP/+}$ and *GATA6*$^{\Delta 14/\Delta 11}$ mutant cells.

(F) Expression of pluripotency (*OCT4*) and definitive endoderm (*SOX17, GATA6* and *GATA4*) markers at day 3 DE in Patient A and Patient B mutant cells.

(D-F) Error bars represent the SE of three independent experiments. $^{*}p < 0.05$, $^{**}p < 0.01$, $^{***}p < 0.001$, $^{****}p < 0.0001$.

Supplemental Figure 3. *GATA6* is a key regulator of the DE transcriptional network.

(A) Heat map illustrating differential gene expression of key germ layer markers via RNA-seq between H9* cells, H9-derived *GATA6*$^{4ins/+}$ and clones 1, 2 and 3 of Patient A mutant cells at the DE stage. $n = 3$ biological replicates for each cell line.

(B, C) Venn diagram indicating the overlap of *GATA6*-bound genes from ChIP-seq at the DE stage with downregulated or upregulated genes of H9-derived *GATA6*$^{4ins/+}$ mutant cells or Patient A compared to H9* cells derived from RNA-seq.

(D, E) Venn diagram indicating the triple overlap of *GATA6*-bound genes from ChIP-seq at the DE stage with downregulated or upregulated genes of H9-derived *GATA6*$^{\Delta 4/\Delta 4}$ mutant cells compared to H9* cells derived from RNA-seq. Key bound genes up- or downregulated are indicated in the respective tables.

(F, G) Enriched gene ontology showing developmental pathways from direct target genes differentially expressed between H9* and H9-derived *GATA6*$^{4ins/+}$ or H9* and Patient A mutant cells derived from BETA analysis.

(H) Motif analysis of up and down target genes derived from BETA analysis.

Supplemental Figure 4. Decreased levels of *GATA6* impact downstream pancreatic differentiation.

(A) Expression of DE (*GATA6, GATA4* and *FOXA2*), pancreatic (*HNF4A, HLXB9* and *PDX1*), and endocrine (*INSULIN*) marker genes in FSPS13.B-derived *GATA6*$^{\Delta 14/+}$, *GATA6*$^{GFP/+}$ mutant cells at key stages of the 24-day pancreatic differentiation protocol (**Supp. Fig. 1A**).

(B) Expression of DE (*GATA6, GATA4* and *FOXA2*), pancreatic (*HNF4A, HLXB9* and *PDX1*), and endocrine (*INSULIN*) marker genes in Patient A and Patient B mutant cells.

(C) Percentage PDX1-positive cells in FSPS13.B-derived *GATA6* $^{\Delta14/+}$ and *GATA6*$^{GFP/+}$ lines at day 12 shown relative to FSPS13.B* (100%) as measured by FACS. Absolute percentage of C-PEPTIDE-positive cells in FSPS13.B* and FSPS13.B-derived *GATA6*$^{\Delta14/+}$ and *GATA6*$^{GFP/+}$ lines at the EP stage (day 24).

(D) Percentage PDX1-positive cells in Patient A and B lines at day 12 shown relative to FSPS13.B (100%) as measured by FACS. Absolute percentage of C-PEPTIDE-positive cells in Patient A and B lines at the EP stage (day 24).

(E) Heat map illustrating differential gene expression of key pancreatic progenitor markers via RNA-seq between H9*cells, H9-derived *GATA6*$^{4ins/+}$ and clones 1, 2 and 3 of Patient A at the PE stage. n = 3 biological replicates for each cell line.

(A-D) Error bars represent the SE of three independent experiments. $^{*}p < 0.05$, $^{**}p < 0.01$, $^{***}p < 0.001$, $^{****}p < 0.0001$.

Supplemental Table 2

TALEN target sites for *GATA6*		
**TALEN pair**	**Name**	**Sequence (5' → 3')**
After first ATG	Left arm	GACTGACGGCGGCTGGT
	Right arm	CCGCACCCGCGGCCCCG
After second ATG	Left arm	GCTGCCCGGCCTACCGT
	Right arm	GGCTGGCCCACTGCCC
**Primers used to screen for mutations**		
**TALEN target site**		**Primer sequence (5' → 3')**
After first ATG	F	CTTTGAGAAGTCAGATCCCATTTGA
	R	CGCCTCCGCTGCCGTATGGAGGGCT
After second ATG	F	CGCCAGCAAGCTGCTGTGGTCCAGC
	R	TCCGCGCACCCGGACGAGAAAGTCC

**Primers used to assemble TALEN repeat arrays**	
**Primer name**	**Primer sequence (5' → 3')**
TALEN-RVDs 1 Fwd	CTGACCCCAGACCAGGTAGTCGCA
TALEN-RVDs 1 Rev	CACGACTTGATCCGGTGTAAGGCCGTGGTCTTGACAAAGG
TALEN-RVDs 2 Fwd	CCTTTGTCAAGACCACGGCCTTACACCGGATCAAGTCGTG
TALEN-RVDs 2 Rev	TACAACTTGATCGGGAGTCAGCCCGTGgtCTTGACAGAGA
TALEN-RVDs 3 Fwd	TCTCTGTCAAGacCACGGGCTGACTCCCGATCAAGTTGTA
TALEN-RVDs 3 Rev	GACCACTTGgtCAGGCGTCAAACCGTGatCTTGACACAAC
TALEN-RVDs 4 Fwd	GTTGTGTCAAGatCACGGTTTGACGCCTGacCAAGTGGTC
TALEN-RVDs 4 Rev	TCCATGATCCTGGCACAGTACAGG
TALEN-RVDs 1-4 Fwd	tcagGGTCTCAGAACCTGACCCCAGACCAGGTAGTC
TALEN-RVDs 1-4 Rev	tcagGGTCTCTAGTCCATGATCCTGGCACAGT
TALEN-RVDs 5-8 Fwd	tcagGGTCTCAGACTGACCCCAGACCAGGTAGTC

TALEN-RVDs 5-8 Rev	tcagGGTCTCTGTCAGTCCATGATCCTGGCACAGT
TALEN-RVDs 9-12 Fwd	tcagGGTCTCATGACCCCAGACCAGGTAGTC
TALEN-RVDs 9-12 Rev	tcagGGTCTCTCAGTCCATGATCCTGGCACAGT
TALEN-RVDs 13-16 Fwd	tcagGGTCTCAACTGACCCCAGACCAGGTAGTC
TALEN-RVDs 13-16 Rev	tcagGGTCTCTTCAGTCCATGATCCTGGCACAGT
**Primers used to construct the donor plasmid**	
**Primer name**	**Primer sequence (5' → 3')**
5' Arm-KpnI-GATA6 Fwd	tcagGGTACCTTTGGGGTCGCCTCGGCTCTGG
5' Arm-GATA6 Rev	CTTGCTCACCATGGTGGCCACGGTCCGGCGCCGCTCCAA
5' Arm-GATA6-emGFP Fwd	CGCCGGACCGTGGCCACCATGGTGAGCAAGGGCGAGGAGC
3' Arm-XbaI-TALEN1 Fwd	tcagTCTAGAAAGCGCTTCGGGGCCGCGGGTG
3' Arm-SacI-TALEN1 Rev	tcagGAGCTCTGGCGCCCCCACGTAGGGCGAG
**Primers used for sequencing donor plasmid**	
**Primer name**	**Primer sequence (5' → 3')**
EmGFP3'-Fwd	TCACATGGTCCTGCTGGAGTTC
BGHpA-mid-Rev	TTAGGAAAGGACAGTGGGAGTG
EmGFP5'-Rev	CGCTGAACTTGTGGCCGTTTAC
EmGFP-mid-Rev	GACCTTGTGGCTGTTGTAGTTG
mPGKpA-Fwd	AAGAAGGGTGAGAACAGAGTACC
M13-Rev (-24)	GGAAACAGCTATGACCATG
M13-Fwd (-20)	GTAAAACGACGGCCAGT
pCAGGS pre-SA Fwd	CTGCTAACCATGTTCATGCCTTC

Supplemental Table 2: Primers supporting Figure 1.

Sequence of left and right TALEN arms for *GATA6* mutant generation at two different cut sites in exon 2, sequence of primers used to screen for mutations, assemble the TALEN repeat arrays, construct and sequence the donor plasmid.

Supplemental Table 3

Gene		Primer sequence (5' → 3')
OCT4	F	AGTGAGAGGCAACCTGGAGA
	R	ACACTCGGACCACATCCTTC
SOX2	F	TGGACAGTTACGCGCACAT
	R	CGAGTAGGACATGCTGTAGGT
BRACHURY	F	TGCTTCCCTGAGACCCAGTT
	R	GATCACTTCTTTCCTTTGCATCAAG
EOMESODERMIN	F	ATCATTACGAAACAGGGCAGGC
	R	CGGGGTTGGTATTTGTGTAAGG
GATA4	F	TCCCTCTTCCCTCCTCAAAT
	R	TCAGCGTGTAAAGGCATCTG
GATA6	F	TGTGCAATGCTTGTGGACTC
	R	AGTTGGAGTCATGGGAATGG
SOX17	F	CGCACGGAATTTGAACAGTA
	R	GGATCAGGGACCTGTCACAC
CXCR4	F	CACCGCATCTGGAGAACCA
	R	GCCCATTTCCTCGGTGTAGTT
FOXA2	F	GGGAGCGGTGAAGATGGA
	R	TCATGTTGCTCACGGAGGAGTA
GCG	F	AAGCATTTACTTTGTGGCTGGATT
	R	TGATCTGGATTTCTCCTCTGTGTCT
HLXB9	F	CACCGCGGGCATGATC
	R	ACTTCCCCAGGAGGTTCGA
HNF4A	F	CATGGCCAAGATTGACAACCT
	R	TTCCCATATGTTCCTGCATCAG
INSULIN	F	GAAGCGTGGCATTGTGGAAC
	R	GCTGCGTCTAGTTGCAGTAGT
NGN3	F	GCTCATCGCTCTCTATTCTTTTGC
	R	GGTTGAGGCGTCATCCTTTCT
NKX6.1	F	GGCCTGTACCCCTCATCAAG
	R	TCCGGAAAAAGTGGGTCTCG
PDX1	F	GATTGGCGTTGTTTGTGGCT
	R	GCCGGCTTCTCTAAACAGGT
SST	F	CCCCAGACTCCGTCAGTTTC
	R	TCCGTCTGGTTGGGTTCAG
PBGD	F	GGAGCCATGTCTGGTAACGG
	R	CCACGCGAATCACTCTCATCT

Supplemental Table 3: Table of forward and reverse primers used for RT-qPCR supporting Fig. 2 and 4, and Supp. Fig. 1, 2, and 4.

Supplemental Table 4

**Primary antibody for Immunofluorescence (IF) staining**	**Dilution ratio**	**Duration**
Goat anti-human Nanog (R&D, #AF1997)	1:100	Overnight
Goat anti-human Sox2 (R&D, #AF2018)	1:100	Overnight
Goat anti-human Oct4 (Santa Cruz, #sc-8628)	1:100	Overnight
Rabbit anti-human GATA6 (Cell Signaling, #5851)	1:200	Overnight
Goat anti-human Sox17 (R&D, #AF1924)	1:200	Overnight
Goat anti-human FoxA2 (R&D, #AF2400)	1:100	Overnight
Goat anti-human PDX1 (R&D, #AF2419)	1:100	Overnight
Mouse anti-human C-Peptide (Acris Antibodies, #BM270S)	1:100	Overnight
Goat anti-human Glucagon G-17 (Santa Cruz, #sc7780)	1:100	Overnight
Rabbit anti-human Somatostatin (Daka, #A0566)	1:200	Overnight
**Secondary antibody for Immunofluorescence (IF) staining**	**Dilution ratio**	**Duration**
Alexa Fluor 568 Donkey Anti-Goat IgG (H+L) (Invitrogen, #A11057)	1:1000	1 hr
Alexa Fluor 568 Donkey Anti-Mouse IgG (H+L) (Invitrogen, #A10037)	1:1000	1 hr
Alexa Fluor 568 Donkey Anti-Rabbit IgG (H+L) (Invitrogen, #A10042)	1:1000	1 hr
Alexa Fluor 488 Donkey anti-Goat IgG (H+L) (Invitrogen, #A11055)	1:1000	1 hr
Alexa Fluor 488 Donkey anti-Mouse IgG (H+L) (Invitrogen, #A21202)	1:1000	1 hr
Alexa Fluor 488 Donkey anti-Rabbit IgG (H+L) (Invitrogen, #A21206)	1:1000	1 hr
Alexa Fluor 647 Donkey anti-Goat IgG (H+L) (Invitrogen, #A21447)	1:1000	1 hr
Alexa Fluor 647 Donkey anti-Mouse IgG (H+L) (Invitrogen, #A31571)	1:1000	1 hr
Alexa Fluor 647 Donkey anti-Rabbit IgG (H+L) (Invitrogen, #A31573)	1:1000	1 hr
**Primary antibody for FACS analysis**	**Dilution ratio**	**Duration**
Goat anti-human Sox17 (R&D, #AF1924)	1:20	2 hr
Rabbit anti-human GATA6 (Cell Signaling, #5851)	1:20	2 hr
Goat anti-human PDX1 (R&D, #AF2419)	1:20	2 hr
Mouse anti-human C-Peptide (Acris Antibodies, #BM270S)	1:100	2 hr

Goat anti-human Glucagon G-17 (Santa Cruz, #sc7780)	1:20	2 hr
Rabbit anti-human Somatostatin (Daka, #A0566)	1:200	2 hr
**Secondary antibody for FACS analysis**	**Dilution ratio**	**Duration**
Alexa Fluor 568 Donkey Anti-Goat IgG (H+L) (Invitrogen, #A11057)	1:1000	30 min
Alexa Fluor 568 Donkey Anti-Mouse IgG (H+L) (Invitrogen, #A10037)	1:1000	30 min
Alexa Fluor 568 Donkey Anti-Rabbit IgG (H+L) (Invitrogen, #A10042)	1:1000	30 min
Alexa Fluor 647 Donkey anti-Mouse IgG (H+L) (Invitrogen, #A31571)	1:1000	30 min
Alexa Fluor 488 Donkey anti-Rabbit IgG (H+L) (Invitrogen, #A21206)	1:1000	30 min
**Conjugated primary and secondary antibody for FACS analysis**	**Dilution ratio**	**Duration**
Anti-Human CD184 (CXCR4) PE (eBioscience, #12-9999-41)	1:50	1 hr
**Primary antibody for western blotting**	**Dilution ratio**	**Duration**
Rabbit anti-human GATA6 (N-terminus; Cell Signaling, #5851)	1:2000	2 hr
Rabbit anti-human GATA6 (C-terminus; Cell Signalling, #4253)	1:2000	2 hr
Rabbit anti-human GATA4 (Cell Signalling, #36966)	1:2000	2 hr
Mouse anti-alpha-Tubulin (Sigma-Aldrich, #T6199)	1:5000	1 hr
**Secondary antibody for western blotting**	**Dilution ratio**	**Duration**
Anti-Rabbit IgG- Peroxidase antibody produced in goat (Sigma-Aldrich, #A6154)	1:10000	1 hr
Anti-Mouse IgG- Peroxidase antibody produced in goat (Sigma-Aldrich, #A5278)	1:10000	1 hr

Supplemental Table 4: Primary, conjugated and secondary antibodies supporting Fig. 1, 2 and 4, and Supp. Fig. 1, 2 and 4.

Tables of primary and secondary antibodies used for Immunofluorescence, FACS and western blotting.

SUPPLEMENTAL EXPERIMENTAL PROCEDURES

Modifications in pancreatic differentiation

(1) 3 μM CHIR99201 was added on day 1, (2) BMP4 and LY294002 were excluded on day 3, (3) 2 μM retinoic acid (RA) and 0.25 μg/ml KAAD-cyclopamine were included on days 10-12 and 16-18, (4) 2 μM RA and 0.1 mM 6-Bnz-cAMP sodium salt (BNZ; Sigma-Aldrich, #B4560) were included on days 13-15, and (5) the protocol was extended from 18 to 24 days where 1% B27, 2 μM RA acid and 0.25 μg/ml KAAD-cyclopamine were added on days 19-24.

Assembly of the TALEN vectors and donor vector

The TALEN vectors were assembled using the Joung Lab REAL Assembly TALEN kit (Addgene, #1000000017) (Sander et al., 2007). The pTAL scaffold was modified to a second generation GoldyTALEN scaffold, which was shown to improve genome editing efficiency (Bedell et al., 2012). In addition, NN repeat variable domains (RVDs) were modified to become NH. Suitable TALEN target sites in the *GATA6* gene were first generated using an online TALEN targeter software tool (Cermak et al., 2011; Doyle et al., 2012). TALEN targets were selected based on higher numbers of HDs (= C) and NHs (= G) for stronger binding (Streubel et al., 2012) and the presence of a restriction enzyme site in the spacer region to aid in screening. For vector construction, the selected target sequences were entered into a ZiFiT targeter software (Sander et al., 2010; Sander et al., 2007). The sequences of the first and second selected TALEN target pairs are 5' GACTGACGGCGGCTGGT 3' (left) and 5' CCGCACCCGCGGCCCCG 3' (right), and 5' GCTGCCCGGCCTACCGT 3' (left) and 5' GGCTGGCCCACTGCCC 3' (right), respectively. The TALEN vectors were then assembled using a three-step PCR approach to combine the RVDs. The success of the TALEN assembly was verified by Sanger sequencing.

Next, the assembled TALEN RVDs were cloned into vectors containing a CAG promoter and a puromycin, zeocin or blasticidin antibiotic resistant gene. Vectors used to generate mutants via the NHEJ pathway contained the puromycin and zeocin antibiotic resistant gene for the left and right TALEN arms, respectively. Vectors used to generate mutants via the HR pathway contained the blasticidin and zeocin antibiotic resistant gene for the left and right TALEN arms, respectively. The final TALEN constructs were then sequenced to confirm that the TALEN arms were cloned in the correct orientation using the following forward and reverse primers 5' AATACGACTCACTATAG 3' and 5' AACTTTTAAACCGGTCTCGAGCTGA 3' respectively.

A donor vector aimed at terminating transcription of *GATA6* prematurely by inserting a 'donor template' through HR was also constructed. Within the donor vector is a cassette which contains 5' and 3' homology arms each 1kb in length recognising the flanking regions of the TALEN 1 target

site, an EmGFP gene, a puromycin antibiotic resistant cassette and a polyA tail. Primers used to construct the donor vector are listed in **Supp. Table 2**. The final construct was sequenced to confirm that the donor vector was cloned successfully. Primers used to sequence the donor vector are listed in **Supp. Table 2**.

Electroporation and screening of drug-resistant clones

TALEN vectors were introduced into cells via electroporation (Human Stem Cell Nucleofector Kit 1, Lonza) using the Amaxa Nucleofector. Briefly, cells were harvested after treatment with StemPro Accutase Cell Dissociation Reagent (Gibco, #A1110501) and counted. 8 x 10^5 cells were used for each electroporation. Electroporation was performed according to the manufacturer's recommendations and cells were plated with ROCK inhibitor Y-27632 (Sigma-Aldrich, #Y0503). 24-hour antibiotic selection using puromycin (1 µg/ml; Sigma-Aldrich, #P8833), zeocin (2.5 µg/ml; Gibco, #R250-01) or blasticidin (3.5 µg/ml; Sigma-Aldrich, #15205) was started 24 hours after electroporation. Individual colony screening was carried out by PCR on genomic DNA with primers listed in **Supp. Table 2**. PCR products were sub-cloned when necessary to determine the precise mutation(s).

Multiplex fluorescence *in situ* hydridization (M-FISH) karyotyping

For each cell line, 10-20 randomly selected metaphases were karyotyped based on multiplex fluorescence *in situ* hydridization (M-FISH) with human 24-colour painting probe and DAPI-banding pattern analyses.

RNA isolation and RT-quantitative (q)PCR

Cells were grown in 12-well plates for total RNA isolation. Three wells were individually harvested per sample to obtain biological replicates. The RNeasy Mini Kit (Qiagen, #74106) together with the Qiacube was used for total RNA extraction. Cell were washed once with D-PBS then lysed with 350 µl of RLT Buffer. Each sample was treated with RNase-Free DNase (Qiagen, #79254). RNA was eluted in a volume of 30 µl. For first strand cDNA synthesis, 500 ng of RNA, random primer (Promega, #C1181) and dNTP (Promega, #U1511) were incubated for 5 min at 65°C then quickly chilled on ice. For reverse transcription of RNA, RNaseOUT Recombinant Ribonuclease Inhibitor (Invitrogen, # 10777019) and SuperScript II Reverse Transcriptase (Invitrogen, #18064014) were incubated with material obtained from the previous step in a PCR machine programmed at 10 min at 25°C for the primer annealing step, 50 min at 42°C for the extension step, and finally 15 min at 70°C for the inactivation of the enzyme. The resulting cDNA was diluted to a final volume of 600 µl with nuclease-free water prior to use for RT-qPCR. RT-qPCR master mix was prepared using Sensi Mix Sybr Low Rox Kit (Bioline, #QT625-20). RT-qPCR reactions were performed using Mx3005P

system (Stratagene) according to the manufacturer's instructions. Samples were run in technical triplicates and normalised to *PBGD*. Gene-specific primers are listed in **Supp. Table 3**.

Immunofluorescence (IF) staining

Cells in 12 well plates were fixed by aspirating the culture media then immediately adding 500 µl of 4% paraformaldehyde (PFA; VWR, #43368.9M) solution and incubating for 20 min at 4°C. They were then washed thrice in D-PBS. To block unspecific binding, cells were incubated in 500 µl of PBST (0.1% Triton X-100 in D-PBS) containing 10% donkey serum (AbD Serotec, #C06SB) per well for 20 min at room temperature. Cells were then incubated overnight at 4°C with 300 µl of primary antibodies diluted in PBST containing 1% donkey serum. Cells were next washed thrice with PBST to remove unbound primary antibodies and thereafter incubated with 300 µl of fluorescence-dye-conjugated secondary antibodies diluted in PBST containing 1% donkey serum in for 1 hr at room temperature. Unbound antibodies were removed by three 5 min washes in D-PBS. 4′,6-Diamidino-2-phenylindole dihydrochloride (DAPI; Sigma-Aldrich, #D-8417) at a dilution of 1:1000 was added to the first wash. Antibodies used for immunostaining are listed in **Supp. Table 4**.

Fluorescence activated cell sorting (FACS) analysis

Cells in 12 well plates were washed twice in D-PBS and incubated in 0.3 ml of Accutase per well for 5 min at 37°C. The Accutase was neutralised by adding 0.6 ml of 5% FBS diluted in D-PBS and the cells were dissociated by gentle pipetting. Cells were re-suspended in D-PBS at approximately 0.1-1 x 10^6 cells/ml and washed twice with D-PBS. They were then pelleted and fixed by re-suspending in 500 µl of 4% PFA solution diluted in D-PBS per well and incubating at for 20 min at 4°C, then washed twice in D-PBS. For all primary antibodies except CXCR4, cells were permeabilised in 500 µl of D-PBS containing 1% Saponin (Sigma-Aldrich, #47036-50G-F) for 30 min at room temperature. Cells were then incubated for 2 hr at room temperature with primary antibody diluted in 100 µl of Staining Solution (1% Saponin and 5% FBS in D-PBS). After which, they were washed three times with 1 ml of Staining Solution per wash and incubated with secondary antibodies diluted in 100 µl of Staining Solution for 30 min at room temperature. Unbound antibody was then removed by three washes in 1 ml of Staining Solution per wash and cells were re-suspended in 200 µl of 2% FBS diluted in D-PBS prior to analysis. For CXCR4 staining, cells were fixed in 4% PFA and washed as described above. Thereafter, primary antibody diluted in 100 µl of 5% FBS in D-PBS was added to the cells and incubated for 1 hr at room temperature. Unbound antibody was then removed by three washes of 1ml 2% FBS in D-PBS per wash. Cells were then re-suspended in 200 µl of 2% FBS in PBS prior to analysis. Analyses were performed using a BD LRSFortessa cell analyser (BD Biosciences). All flow cytometry experiments were gated using unstained cells. Data analyses were performed on FlowJo. On all flow cytometry plots, the undifferentiated population is shown in blue.

All gates shown on scatterplots were set according to the undifferentiated population control. Antibodies used for FACS analyses are listed in **Supp. Table 4**.

Western blotting

Cells were washed once in D-PBS and incubated in 0.5 ml of Accutase per well of a 6 well plate for 5 min at 37°C. The Accutase was neutralised by adding 1 ml of 5% FBS diluted in D-PBS per well and the cells were dissociated by gentle pipetting. The cells were washed twice with D-PBS and pelleted by centrifuging at 1,200 rpm. The pelleted cells were re-suspended in 50-200 µl of Lysis Buffer (50 mM Tris-Cl pH 7.5, 150 mM NaCl, 1% Triton X-100, 10% glycerol, 0.1% deocycholate, 25 mM β-glycerophosphate) containing freshly added inhibitors cOmplete Protease Inhibitor Cocktail (Roche, #11697498001), Sodium Fluoride (NaF; New England Biolabs, #P0759), Sodium Vanadate (Na_3VO_4; New England Biolabs, #P0758). The cell lysates were kept on ice for at least 15 min, vortexed at maximum speed for 15 s then centrifuged for 30 min at 15,000 g at 4°C. The supernatants were collected and protein concentrations were determined by Bradford assay (Protein Assay Dye Reagent Concentrate, Bio-Rad) according to the manufacturer's protocol. The protein concentrations of the cell lysates were normalised to 10 µg of protein for probing with *GATA6* and *GATA4* and 1 µg for probing with alpha-tubulin. The normalised cell lysates were heat denatured at 98°C in the presence of Laemmli Sample Buffer (Bio-Rad) and β-mercaptoethanol for 5 min, then subjected to SDS-PAGE electrophoresis on NuPAGE Novex 4-12% Bis-Tris Protein Gels using the XCell SureLock Mini-Cell (Invitrogen) system. The separated proteins were next transferred from the gel onto Immun-Blot PVDF membrane (Bio-Rad, #162-0177) using Mini Trans-Blot Cell (Bio-Rad) at 25 V overnight at 4°C. Membranes were blocked in 5% Blotting-Grade Blocker (Bio-Rad, #170-6404) diluted in 0.1% Triton X-100 in D-PBS (PBST) for 1 hr at room temperature. Primary antibodies were incubated for 2 hr at room temperature. Membranes were then washed and incubated with horseradish peroxidase (HRP)-conjugated secondary antibodies for 1 hr at room temperature. Unbound antibodies were removed by three 10 min washes in PBST. Proteins were detected via chemiluminescence using SuperSignal West Femto Maximum Sensitivity Substrate (ThermoFisher Scientific, #PI34095) and finally developed using Amersham Hyperfilm ECL (GE Healthcare). Antibodies used for western blotting are listed in **Supp. Table 4**.

Chromatin Immunoprecipitation (ChIP)

Co-binding of DNA to DNA-binding proteins was determined by ChIP against *GATA6* (Cell Signaling, #5851) on approximately 1 x 10^7 cells per antibody or control sample. Cells were cross-linked with 1% formaldehyde (ThermoFisher UK, #11586711) for 10 min at room temperature. The reaction was quenched with 0.125 M glycine (Millipore, #357002) for 5 min. Cells were washed twice with ice-cold PBS then collected in ice-cold PBS containing freshly-added protease inhibitors (10 µl/ml of 5 mg/ml phenylmethylsulfonylfluoride (PMSF; Sigma-Aldrich, #93482), 10 µl/ml of 1

M Sodium Butyrate (Sigma-Aldrich, #303410) and 1 µl/ml of 1 mg/ml Leupeptin (Roche, #11017101001)). Harvested cells were centrifuged for 5 min at 1,200 rpm at 4°C to pellet. For all subsequent steps, the samples were kept on ice. For all subsequent buffers used, the aforementioned protease inhibitors were added freshly to the buffers before use. The pelleted cells were subsequently re-suspended in 2 ml of ice-cold Cell Lysis Buffer (10 mM Tris-Cl pH 8.0, 10 mM NaCl and 0.2% NP-40), incubated on ice for 10 min, and then centrifuged for 5 min at 1,800 rpm at 4°C. The supernatant was discarded and the pellet was gently re-suspended in 1.25 ml of ice-cold Nuclear Lysis Buffer (50 mM Tris-Cl pH 8.0, 10 mM EDTA and 1% SDS) and incubated on ice for 10 min. 0.75 ml of ice-cold IP Dilution Buffer (20 mM Tris-Cl pH 8.0, 2 mM EDTA, 150 mM NaCl, 0.01% SDS, 1% Triton X-100) was then added.

The chromatin was sonicated using Diagenode Biorupter Pico in 15 ml Diagenode sonication tubes containing sonication beads (Diagenode, #C01020031) pre-washed with 10 ml D-PBS and 10 ml IP Dilution Buffer for 10 cycles of 30s on/45s off. Chromatin fragments were determined by a Bioanalyser (Agilent 2100 Bioanalyzer) and analysed using High Sensitivity DNA Kit (Agilent, #5067-4626) according to the manufacturer's protocol. The sonicated chromatin was then centrifuged at 14,000 rpm for 10 min at 4°C to pellet debris. 3.5 ml of IP Dilution Buffer was added to the supernatant and mixed gently. The cross-linked DNA was pre-cleared by incubating with rotation 10 µg of rabbit IgG (Sigma-Aldrich, #I5006) for 1 hr at 4°C, followed by incubating with rotation 100 µl of Protein G agarose beads (50% v/v; Roche, #11243233001) pre-washed twice with D-PBS for 1 hr at 4°C. The samples were then centrifuged for 3 min at 3,000 rpm at 4°C and the supernatant was transferred to a fresh 15 ml tube. An aliquot of 300 µl for Input sample was taken and stored at 4°C.

10 µg of *GATA6* antibody or rabbit IgG control was added per sample and incubated rotating overnight at 4°C. Antibody-bound chromatin was then collected using 60 µl of Protein G agarose beads (50% v/v) pre-washed twice with D-PBS by incubating with rotation for 1 hr at 4°C. Thereafter, the tubes were centrifuged for 3 min at 3,000 rpm at 4°C. The supernatant was discarded and the pellet containing the protein-DNA complexes bound onto the protein G agarose beads were kept.

Samples were washed twice with 500 µl of IP Wash Buffer 1 (20 mM Tris-Cl pH 8.0, 2 mM EDTA, 50 mM NaCl, 0.1% SDS and 1% Triton X-100), twice with 500 µl of IP Wash Buffer 2 (10 mM Tris-Cl pH 8.0, 1 mM EDTA, 0.25 M LiCl, 1% NP-40 and 1% Sodium deoxycholic acid), twice with 500 µl of TE Buffer (10mM Tris-Cl pH 8.0, 1mM EDTA) then eluted by washing twice with 150 µl of Elution Buffer (100 mM $NaHCO_3$ and 1% SDS). ChIP and Input DNA cross-links were reversed and RNA degraded by adding 1 µl of 1 mg/ml RNase A and 18 µl of 5M NaCl and incubating at 67°C in a heat block with shaking at 1,300 rpm overnight. Protein was degraded by adding 3 µl of 20 mg/ml Proteinase K and incubating for 3 hrs at 45°C in a heat block with shaking at 1,300 rpm. Pulled-down

genomic DNA was extracted using 300 µl of phenol/chloroform wash. The samples were next incubated with 30 µl of 3M NaAc pH 5.2 (Ambion, #AM9740), 30 µg glycoblue (Ambion, #AM9516) and 750 µl of 100% ethanol for at least 30 min at -80°C to precipitate the DNA. Precipitated DNA was pelleted by centrifuging at 14,000 rpm for 30 min at 4°C. The DNA pellet was then washed with ice-cold 70% ethanol then air dried. 70 µl of deionised water was added to Input samples whereas 30 µl of deionised water was added to ChIP samples.

RNA-seq data analysis

Tophat v2 (Kim et al., 2013) was used to align the reads to the reference human genome assembly (GRCh38/hg20), using Ensembl release 76 as reference transcriptome. featureCounts was used on paired-end reads to count fragments in annotated gene features, with parameters '-p -T 8 -t exon -g gene_id' (Liao et al., 2014). DESeq2 R/Bioconductor package was used in differential gene expression analysis between samples, requiring at least a twofold expression change and adjusted using the Benjamini–Hochberg procedure to p-value smaller than 0.01 (Love et al., 2014) for a gene to be declared as differentially expressed. The function 'rpkm' in the R/Bioconductor package edgeR (give reference) was used with default parameters to normalize count gene expression (Robinson et al., 2010). Raw bedGraphs were normalized per million mapped reads in the library per library size in all ChIP-seq and RNA-seq samples (Conesa et al., 2016; Genome Biol). Genome browser panels were generated using IGV (Thorvaldsdóttir et al., 2013). Gene Ontology (GO) analyses were performed using Amigo2 separately for up- and down- regulated differentially expressed genes (Carbon et al., 2009). Spearman's correlation values were calculated in R for FPKM expression values of genes expressed at more than 5 FPKM in at least one of the samples under comparison.

ChIP-seq data analysis

We followed recommended guidelines in the analysis of ChIP-seq data for read mapping, normalization, peak-calling and assessment of reproducibility among biological replicates (Bailey et al, 2013. PloS Comput Biol). Paired-end reads were aligned to the reference human genome assembly (GRCh38/hg20) using BWA v0.5.10 (Li and Durbin, 2009) with -q 15 and default for the rest of parameters. Reproducibility between replicates was first assessed using the Pearson Correlation Coefficient (PCC) for the two biological replicates, using the genome-wide normalized read (extended to 300 bp) count distribution on a single nucleotide resolution. For this, we used the UCSC tool bigwigCorrelate provided in http://hgdownload.cse.ucsc.edu/admin/exe/. PCC was equal to 0.949326.

Peak calling was performed using MACS version 2.0.10 (Zhang et al., 2008) allowing a p-value cut-off of 1e-3 and default settings for all other parameters. Relaxed thresholds are suggested in order to enable the correct computation of IDR values (Landt et al., 2012). Following the recommendations

for the analysis of self-consistency and reproducibility between replicates, the negative control samples (IgG and input DNA) were combined into one single control; code for IDR analysis was downloaded from https://sites.google.com/site/anshulkundaje/projects/idr (Li et al., 2011). This is also beneficial as control samples with substantially higher number of reads are recommended for peak calling (Bailey et al., 2013). 37,777 and 35,408 peaks were found for first and second replicate, respectively, with >26k of regions of direct overlap.

To estimate the Irreproducible Discovery Rate (IDR) between replicates, top 35k peaks for each biological replicate were submitted for IDR analysis. For IDR computation using MACS results, we used p-values rather than q-values as suggested in https://sites.google.com/site/anshulkundaje/projects/idr (Li et al., 2011). The number of peaks found passing a threshold of IDR ≤ 5% (12,107) was selected as a conservative estimated number of candidate transcription factor binding sites. After excluding autosomal and sex chromosomes, we have 12,098 peaks. We searched for the closest gene feature in ensembl_76_transcriptome using BEDTools closest with parameter '-D b' (Quinlan and Hall, 2010). To associate peak to genes in a 20kb window, we ran BEDTools window with '-w 20000' and own R scripts.

Co-localization plots of the transcription factors *GATA6*, *EOMES* and *SMAD2/3* ChIP-seq, was generated with deepTools (Ramirez et al., 2014). The input data was obtained by combining our ChIP data of H9 cells at day 3 (*GATA6*) with previously published *EOMES* (uploaded to Gene Expression Omnibus with accession number GSE26097) (Teo et al., 2011) and *SMAD2/3* ChIP data (uploaded to Gene Expression Omnibus with accession number GSE19461) (Brown et al., 2011). To make the results more comparable, we remapped the 3 data sets with STAR v2.5.1a (Dobin et al., 2013) (BWA failed on short single end SMAD reads) and processed them with MACS version 2.0.10 and IDR as described earlier. The resulting peak files (bed format) were used as input for deepTools. The mapped read files (bam format) were pre-processed with deepTools' "bamCompare" function (bin size = 50, assumed genome size = 2451960000 bp, ignoring chromosomes X and Y for normalization and extending single end reads by 250bp).

SUPPLEMENTARY REFERENCES

Bailey, T., Krajewski, P., Ladunga, I., Lefebvre, C., Li, Q., Liu, T., Madrigal, P., Taslim, C., and Zhang, J. (2013). Practical guidelines for the comprehensive analysis of ChIP-seq data. PLoS Comput Biol *9*, e1003326.

Bedell, V.M., Wang, Y., Campbell, J.M., Poshusta, T.L., Starker, C.G., Krug, R.G., 2nd, Tan, W., Penheiter, S.G., Ma, A.C., Leung, A.Y., *et al.* (2012). In vivo genome editing using a high-efficiency TALEN system. Nature *491*, 114-118.

Brown, S., Teo, A., Pauklin, S., Hannan, N., Cho, C.H., Lim, B., Vardy, L., Dunn, N.R., Trotter, M., Pedersen, R., *et al.* (2011). Activin/Nodal signaling controls divergent transcriptional networks in human embryonic stem cells and in endoderm progenitors. Stem Cells *29*, 1176-1185.

Carbon, S., Ireland, A., Mungall, C.J., Shu, S., Marshall, B., Lewis, S., the Ami, G.O.H., and the Web Presence Working, G. (2009). AmiGO: online access to ontology and annotation data. Bioinformatics (Oxford, England) *25*, 288-289.

Cermak, T., Doyle, E.L., Christian, M., Wang, L., Zhang, Y., Schmidt, C., Baller, J.A., Somia, N.V., Bogdanove, A.J., and Voytas, D.F. (2011). Efficient design and assembly of custom TALEN and other TAL effector-based constructs for DNA targeting. Nucleic Acids Research *39*, e82-e82.

Dobin, A., Davis, C.A., Schlesinger, F., Drenkow, J., Zaleski, C., Jha, S., Batut, P., Chaisson, M., and Gingeras, T.R. (2013). STAR: ultrafast universal RNA-seq aligner. Bioinformatics *29*, 15-21.

Doyle, E.L., Booher, N.J., Standage, D.S., Voytas, D.F., Brendel, V.P., VanDyk, J.K., and Bogdanove, A.J. (2012). TAL Effector-Nucleotide Targeter (TALE-NT) 2.0: tools for TAL effector design and target prediction. Nucleic Acids Research *40*, W117-W122.

Kim, D., Pertea, G., Trapnell, C., Pimentel, H., Kelley, R., and Salzberg, S.L. (2013). TopHat2: accurate alignment of transcriptomes in the presence of insertions, deletions and gene fusions. Genome Biol *14*, R36.

Landt, S.G., Marinov, G.K., Kundaje, A., Kheradpour, P., Pauli, F., Batzoglou, S., Bernstein, B.E., Bickel, P., Brown, J.B., Cayting, P.*, et al.* (2012). ChIP-seq guidelines and practices of the ENCODE and modENCODE consortia. Genome research *22*, 1813-1831.

Li, H., and Durbin, R. (2009). Fast and accurate short read alignment with Burrows-Wheeler transform. Bioinformatics *25*, 1754-1760.

Li, Q., Brown, J.B., Huang, H., and Bickel, P.J. (2011). Measuring reproducibility of high-throughput experiments. 1752-1779.

Liao, Y., Smyth, G.K., and Shi, W. (2014). featureCounts: an efficient general purpose program for assigning sequence reads to genomic features. Bioinformatics *30*, 923-930.
Love, M.I., Huber, W., and Anders, S. (2014). Moderated estimation of fold change and dispersion for RNA-seq data with DESeq2. Genome Biol *15*, 550.

Quinlan, A.R., and Hall, I.M. (2010). BEDTools: a flexible suite of utilities for comparing genomic features. Bioinformatics (Oxford, England) *26*, 841-842.

Ramirez, F., Dundar, F., Diehl, S., Gruning, B.A., and Manke, T. (2014). deepTools: a flexible platform for exploring deep-sequencing data. Nucleic Acids Res *42*, W187-191.

Robinson, M.D., McCarthy, D.J., and Smyth, G.K. (2010). edgeR: a Bioconductor package for differential expression analysis of digital gene expression data. Bioinformatics *26*, 139-140.

Sander, J.D., Maeder, M.L., Reyon, D., Voytas, D.F., Joung, J.K., and Dobbs, D. (2010). ZiFiT (Zinc Finger Targeter): an updated zinc finger engineering tool. Nucleic Acids Research.

Sander, J.D., Zaback, P., Joung, J.K., Voytas, D.F., and Dobbs, D. (2007). Zinc Finger Targeter (ZiFiT): an engineered zinc finger/target site design tool. Nucleic Acids Research *35*, W599-W605.

Streubel, J., Blucher, C., Landgraf, A., and Boch, J. (2012). TAL effector RVD specificities and efficiencies. Nat Biotech *30*, 593-595.

Teo, A.K., Arnold, S.J., Trotter, M.W., Brown, S., Ang, L.T., Chng, Z., Robertson, E.J., Dunn, N.R., and Vallier, L. (2011). Pluripotency factors regulate definitive endoderm specification through eomesodermin. Genes Dev *25*, 238-250.

Thorvaldsdóttir, H., Robinson, J.T., and Mesirov, J.P. (2013). Integrative Genomics Viewer (IGV): high-performance genomics data visualization and exploration. Briefings in Bioinformatics *14*, 178-192.

Zhang, Y., Liu, T., Meyer, C.A., Eeckhoute, J., Johnson, D.S., Bernstein, B.E., Nusbaum, C., Myers, R.M., Brown, M., Li, W.*, et al.* (2008). Model-based analysis of ChIP-Seq (MACS). Genome Biol *9*, R137.
