# Supplementary material for: GATA6 Cooperates with EOMES/SMAD2/3 to Deploy the Gene Regulatory Network Governing Human Definitive Endoderm and Pancreas Formation
Source: Stem Cell Reports. 2019 Jan 8;12(1):57–70. doi: 10.1016/j.stemcr.2018.12.003 (PMC6335596; doi:10.1016/j.stemcr.2018.12.003)
Supplement: Document S2. Article plus Supplemental Information [file mmc3.pdf]

Stem Cell Reports
Article
ISSCR
OPEN ACCESS

GATA6 Cooperates with EOMES/SMAD2/3 to Deploy the Gene Regulatory Network Governing Human Definitive Endoderm and Pancreas Formation

Crystal Y. Chia,[1,2] Pedro Madrigal,[6] Simon L.I.J. Denil,[2] Iker Martinez,[1] Jose Garcia-Bernardo,[1] Ranna El-Khairi,[1] Mariya Chhatriwala,[1] Maggie H. Shepherd,[3] Andrew T. Hattersley,[3] N. Ray Dunn,[2,4,5,7,*] and Ludovic Vallier[1,6,7,*]
[1]Wellcome Trust Sanger Institute, Hinxton, Cambridge, UK
[2]Institute of Medical Biology, A*STAR (Agency for Science, Technology and Research), 8A Biomedical Grove, #06-06 Immunos, 138648, Singapore
[3]Institute of Biomedical and Clinical Science, University of Exeter Medical School, Level 3 RILD Building, Barrack Road, Exeter EX25DW, UK
[4]Lee Kong Chian School of Medicine, Nanyang Technological University, 50 Nanyang Avenue, 639798, Singapore
[5]School of Biological Sciences, Nanyang Technological University, 60 Nanyang Drive, 637551, Singapore
[6]Wellcome Trust-Medical Research Council Cambridge Stem Cell Institute, Anne McLaren Laboratory for Regenerative Medicine, University of Cambridge, Cambridge, UK, and Department of Surgery, University of Cambridge, Cambridge, UK
[7]Co-senior author
*Correspondence: ray.dunn@imb.a-star.edu.sg (N.R.D.), lv225@cam.ac.uk (L.V.)
https://doi.org/10.1016/j.stemcr.2018.12.003

SUMMARY

Heterozygous *de novo* mutations in *GATA6* are the most frequent cause of pancreatic agenesis in humans. In mice, however, a similar phenotype requires the biallelic loss of *Gata6* and its paralog *Gata4*. To elaborate the human-specific requirements for GATA6, we chose to model *GATA6* loss *in vitro* by combining both gene-edited and patient-derived pluripotent stem cells (hPSCs) and directed differentiation toward β-like cells. We find that *GATA6* heterozygous hPSCs show a modest reduction in definitive endoderm (DE) formation, while *GATA6*-null hPSCs fail to enter the DE lineage. Consistent with these results, genome-wide studies show that GATA6 binds and cooperates with EOMES/SMAD2/3 to regulate the expression of cardinal endoderm genes. The early deficit in DE is accompanied by a significant reduction in PDX1^{+} pancreatic progenitors and C-PEPTIDE^{+} β-like cells. Taken together, our data position GATA6 as a gatekeeper to early human, but not murine, pancreatic ontogeny.

INTRODUCTION

Pancreatic agenesis is an extremely rare human condition resulting from the impaired formation of the pancreas during embryonic development. Clinically, patients can entirely lack the pancreas or present with only a partially formed organ (hypoplasia). The majority of patients have complete absence of a functioning pancreas, resulting in intrauterine growth retardation, neonatal diabetes, and exocrine pancreatic failure, and thus require insulin and exocrine enzyme replacement therapy. Less commonly, less severely affected patients can display a reduction in total islet number or insulin-secreting β cells and present diabetic symptoms during adolescence or adulthood.

The vast majority of human pancreatic agenesis cases owe their genetic origins to mutations in a small handful of pancreatic regulatory genes. The first described is *Pancreatic and Duodenal Homeobox 1* (*Pdx1*) (Schwitzgebel, 2014; Stoffers et al., 1997). In mice, *Pdx1* transcripts label the incipient pancreatic primordium—two epithelial buds that are situated dorsally and ventrally on opposite sides of the posterior foregut around embryonic day 9.5 (Jørgensen et al., 2007; Pan and Wright, 2011). In *Pdx1*-null mutant mice, these buds initially form but quickly regress, resulting in complete pancreatic agenesis, severe hyperglycemia, and death within a few days of birth (Ahlgren et al., 1996; Jonsson et al., 1994; Offield et al., 1996). PDX1 similarly labels the human embryonic dorsal and ventral foregut around Carnegie stage 12 (29–31 days post conception) (Jennings et al., 2013). Significantly, the pathology of human patients with homozygous or compound heterozygous mutations in *PDX1* mirrors the agenesis phenotype observed in *Pdx1*-deficient mice (Schwitzgebel et al., 2003; Stoffers et al., 1997).

The most common cause of pancreatic agenesis in humans is heterozygous mutations in the *GATA6* gene (De Franco et al., 2013; Lango Allen et al., 2011). *GATA6* encodes a highly conserved zinc-finger transcription factor that recognizes and binds the (A/T)GATA(A/G) regulatory motif, two of which are located in the mouse *Pdx1* and human *PDX1* promoters (Carrasco et al., 2012; Lentjes et al., 2016; Patient and McGhee, 2002; Viger et al., 2008; Xuan et al., 2012). GATA6, along with its five other family members (GATA1–5), functions in diverse cellular contexts, from coordinating morphogenesis during embryonic development to the maintenance of lineage-specific gene expression in adult hematopoietic stem cells (Lentjes et al., 2016; Viger et al., 2008). *Gata6* is expressed in the definitive endoderm (DE) that emerges during gastrulation, as well as its derivative the gut tube epithelium and the early pancreas primordium (Freyer et al., 2015; Morrisey et al., 1996). *Gata6* expression persists as the pancreas undergoes branching

This is an open access article under the CC BY license (http://creativecommons.org/licenses/by/4.0/).

morphogenesis, becoming restricted in later development to the ductal epithelial compartment and a subset of endocrine cells (Decker et al., 2006; Ketola et al., 2004).

In contrast to *PDX1*, *GATA6* mutations that result in pancreatic agenesis are heterozygous and predominantly *de novo* (Chao et al., 2015; De Franco et al., 2013; Lango Allen et al., 2011; Stanescu et al., 2015; Suzuki et al., 2014). The majority of cases have full pancreatic agenesis, but there are some associated with incomplete penetrance, resulting in a broad spectrum of clinical manifestations (De Franco et al., 2013). At the extreme, family members with the same inherited *GATA6* allele can present with markedly different phenotypes (Bonnefond et al., 2012; Yau et al., 2017; Yorifuji et al., 2012). In addition, *GATA6* patients usually display a number of extrapancreatic abnormalities, including congenital heart defects, as well as several whose origins are endodermal—hepatobiliary malformations, gall bladder agenesis, and gut herniation (Chao et al., 2015; De Franco et al., 2013; Lango Allen et al., 2011).

Given the observations that haploinsufficiency results in severe pancreatic and non-pancreatic anomalies in humans, it is surprising that *Gata6* heterozygous null mice are viable and fertile, with no reported abnormalities (Koutsourakis et al., 1999; Morrisey et al., 1998). In a recent study, Schrode et al. (2014) showed that the specification of the extraembryonic primitive endoderm entirely fails in *Gata6* homozygous embryos at the blastocyst stage, while in a series of older reports *Gata6*-null mutant embryos were recovered at post-implantation stages with defects in the cardiac mesoderm and visceral endoderm (Koutsourakis et al., 1999; Morrisey et al., 1998). Interestingly, tetraploid complementation experiments between wild-type embryos and *Gata6*-deficient embryonic stem cells, a technique that overcomes the early lethality resulting from the absence of Gata6 in the extraembryonic lineages, reveal that *Gata6*-deficient cells can indeed contribute descendants to the DE in chimeric embryos (Zhao et al., 2005). Moreover, conditional loss of *Gata6* specifically in $Pdx1^{+}$ pancreatic progenitors has no impact on pancreatic morphogenesis. Only when a closely related gene, *Gata4*, is simultaneously deleted is an agenesis phenotype recovered that resembles *GATA6* heterozygous human patients (Carrasco et al., 2012; Xuan et al., 2012).

The striking discrepancy between the mouse and the human phenotypes and the complex genetic landscape of *GATA6* agenesis patients led us to model *GATA6* deficiency *in vitro* using human pluripotent stem cells (hPSCs). We generated a large panel of heterozygous, homozygous, and compound heterozygous *GATA6* mutations by performing genome editing in human embryonic stem cells (hESCs) and human induced pluripotent stem cells (hiPSCs). We additionally derived hiPSCs from two *GATA6* heterozygous pancreatic agenesis patients. Subjecting these *GATA6* heterozygous hPSCs to directed differentiation into the pancreatic lineage unexpectedly revealed a modest requirement for wild-type *GATA6* gene dosage for robust formation of the DE. In contrast to the mouse, complete loss of *GATA6* abrogates DE production. Consistent with these results, genome-wide studies show that GATA6 binds and cooperates with EOMES/SMAD2/3 to regulate the expression of cardinal endoderm genes. In addition, *GATA6* haploinsufficiency diminishes the ability of those DE cells that form to become $PDX1^{+}$ pancreatic progenitors and to further mature into C-PEPTIDE-containing β-like cells. These findings show that in humans, the formation of DE and acquisition of pancreatic fate are exquisitely sensitive to *GATA6* gene dosage.

RESULTS

GATA6 Expression during Directed Differentiation of hPSCs into the Endocrine Lineage

Consistent with *Gata6* expression in the mouse embryo, we previously showed that *GATA6* is activated during the early differentiation of hESCs into the DE lineage (Teo et al., 2015; Vallier et al., 2009). We next determined the precise expression kinetics of *GATA6* during extended differentiation into the pancreatic lineage using the well-characterized hESC line H9 and a slightly revised version of an 18-day chemically defined protocol previously published by our group (Figure S1A and see Experimental Procedures for complete details) (Cho et al., 2012). *GATA6* transcripts are not detected in undifferentiated hESCs, but are abundant by day 3, a time point characterized by the expression of canonical DE markers (*SOX17*, *GATA4*, *FOXA2*, and *HNF4A*) (Figure S1B). Roughly, ~75% and ~98% of cells on day 3 are $SOX17^{+}$ and $GATA6^{+}$, respectively (Figure S1D). *GATA6* expression persists from day 6 onward, coinciding with the activation of the signature pancreatic lineage marker *PDX1* (Figure S1B). By day 12, *GATA6* is co-expressed with genes associated with endocrine commitment (*NGN3* and *NKX6-1*), with approximately 76% and 88% of the differentiated cells $PDX1^{+}$ or $GATA6^{+}$, respectively (Figures S1B and S1E). The expression of islet hormone genes (*INSULIN*, *GLUCAGON*, and *SOMATOSTATIN*) increases from day 12 (Figure S1B). Importantly, immunofluorescence (IF) staining reveals co-localization of SOX17 and GATA6 in day 3 DE as well as PDX1 and GATA6 in day 12 pancreatic endoderm (PE) (Figure S1C). These data were confirmed in a healthy hiPSC line, FSPS13.B, hereafter designated 13.B (Figures S2A–S2C). Taken together, these findings establish developmental windows where *GATA6* insufficiency can result in the pancreatic hypoplasia observed in human *GATA6* heterozygous patients.

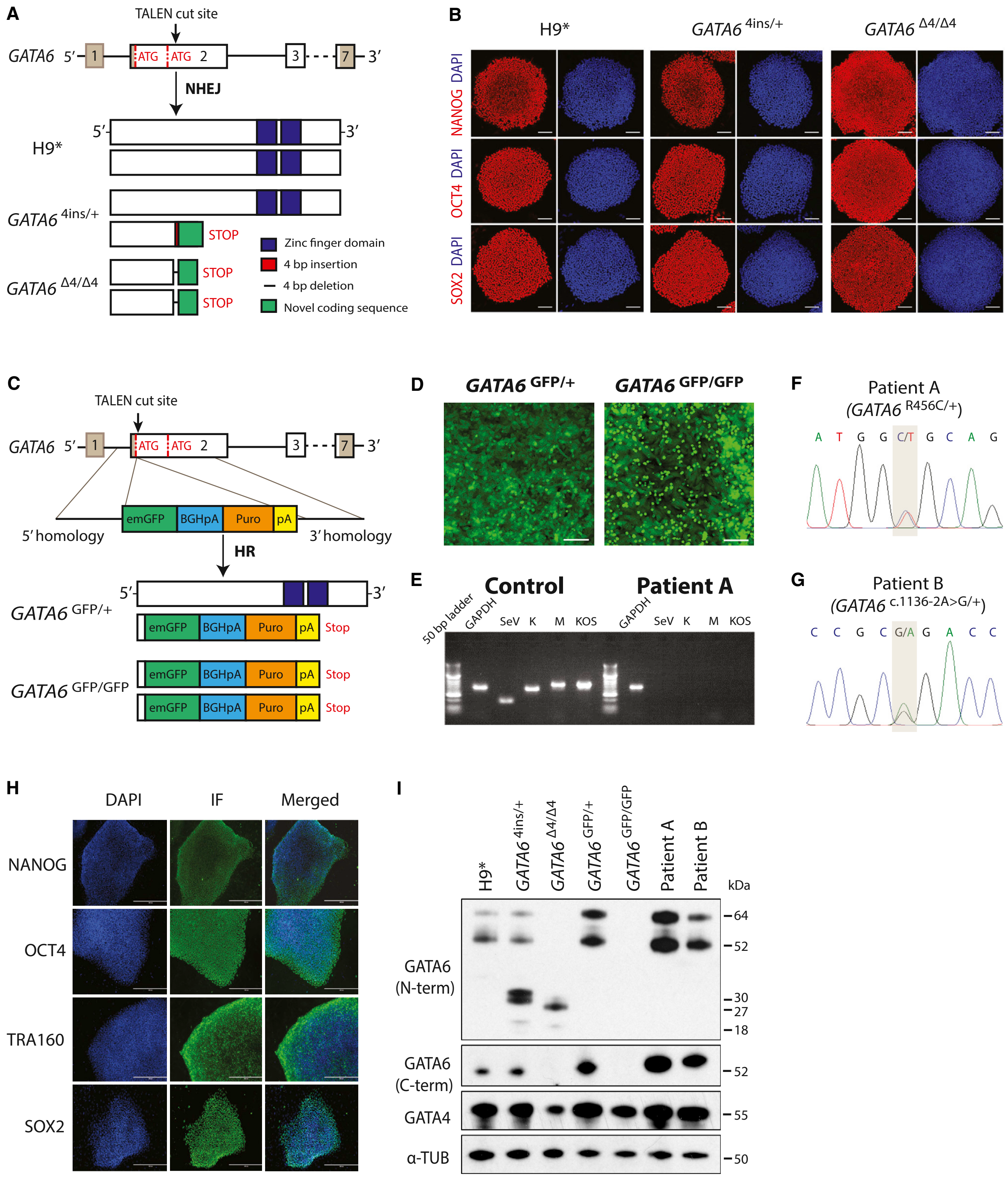

Figure 1. Derivation and Characterization of *GATA6* Mutant Lines

(A) Schematic of the *GATA6* locus. Gray shading highlights the 5′ and 3′ untranslated regions. The TALEN cut site lies downstream of the second start ATG in exon 2. Successful gene editing in H9 cells yielded a *GATA6* heterozygous line containing a 4-bp insertion ($GATA6^{4ins/+}$)

(legend continued on next page)

Generation of *GATA6* Mutant Alleles Using TALENs and Derivation of hiPSCs from Two Independent *GATA6* Heterozygous Patients

To pinpoint the precise role of *GATA6* in the human pancreatic lineage, we performed genome-editing in hPSCs as well as isolated patient-derived hiPSCs to generate a panel of *GATA6* mutant alleles to model pancreatic agenesis *in vitro*. The human *GATA6* gene is transcribed from two distinct promoter regions, contains two initiation codons in exon 2 (a second at Met147), and consequently encodes two GATA6 protein isoforms, with masses of 60 and 45 kDa, respectively (Figure 1A; Brewer et al., 1999). We initially targeted both H9 and 13.B at a TALEN cut site immediately 3′ of the first ATG in *GATA6*. Despite the introduction of frameshift mutations that result in premature stop codons, translation still initiated at the second ATG, producing the shorter GATA6 isoform at wild-type levels (data not shown). Thus, in subsequent experiments, we targeted *GATA6* at a second TALEN cut site 3′ of the second ATG (Figure 1A) and successfully recovered *GATA6* heterozygous ($GATA6^{c.618\_619insTGCA/+}$, hereafter $GATA6^{4ins/+}$) and homozygous ($GATA6^{c.611\_614delACCT/c.611\_614delACCT}$, hereafter $GATA6^{\Delta4/\Delta4}$) mutations in H9 cells. We generated similar insertion or deletion alleles, both heterozygous ($GATA6^{c.del614\_627TGCAGGGGTCGGGC/+}$, hereafter $GATA6^{\Delta14/+}$) and compound heterozygous ($GATA6^{c.del614\_627TGCAGGGGTCGGGC/c.del613\_623CTGCAGGGGTC}$, hereafter $GATA6^{\Delta14/\Delta11}$), in 13.B. In parallel, we inserted an emerald GFP (emGFP) reporter in-frame with the first *GATA6* ATG via homologous recombination ($GATA6^{GFP/+}$) in H9 cells (Figures 1C and 1D) and 13.B cells. An H9 $GATA6^{GFP}$ homozygous clone was also recovered ($GATA6^{GFP/GFP}$) (Figure 1C). Unfortunately, no 13.B $GATA6^{GFP}$ homozygous clone was recovered despite numerous attempts. Control TALEN-targeted lines that harbor no mutations in *GATA6* (designated H9* or 13.B*) served as wild-type, isogenic positive controls for differentiation experiments involving genome-edited hPSCs. Last, we obtained fibroblasts from two *GATA6* heterozygous patients, whose mutations were previously described (De Franco et al., 2013; Yu et al., 2014). Patient A contains a missense mutation (c.1366C>T) at a highly conserved amino acid within the second zinc-finger DNA-binding domain (Arg456Cys) (Figure 1F), while patient B contains a splice acceptor mutation in exon 3 ($GATA6^{c.1136-2A>G/+}$) (Figure 1G). Three independent hiPSC clones were isolated for each patient line. All hESC and hiPSC lines were found to have a normal karyotype by multiplex fluorescence *in situ* hybridization (see Supplemental Experimental Procedures) and assayed by immunohistochemistry to confirm their pluripotency (Figures 1B and 1H and data not shown). hiPSC lines were also monitored for absence of the Sendai virus (Figure 1E).

Differentiation of *GATA6* Mutant hPSC Lines into the Definitive Endoderm Lineage

Mutant lines were next differentiated to the DE stage and GATA6 protein levels determined by western blot using anti-N- and anti-C-terminal GATA6 antibodies (Figure 1I). In H9* DE cells, both GATA6 isoforms are detected by the N-terminal antibody, whereas the C-terminal antibody predominantly recognizes the short isoform (Figure 1I) (Brewer et al., 1999). $GATA6^{4ins/+}$ and $GATA6^{\Delta4/\Delta4}$ contain frameshift mutations that result in truncated partial

and a homozygous line with an identical 4-bp deletion on each chromosome ($GATA6^{\Delta4/\Delta4}$). Each mutation results in the addition of novel coding sequence (green) and a premature stop. H9* cells were subjected to gene editing and selection, but have no mutation in *GATA6*.
(B) OCT4, SOX2, and NANOG immunofluorescence in H9*, $GATA6^{4ins/+}$, and $GATA6^{\Delta4/\Delta4}$ lines confirms pluripotency in gene-edited clones. Scale bars, 100 μm.
(C) A second TALEN cut site downstream of the first ATG in exon 2 of *GATA6* is depicted. Cartoon schematic of the "knockin" vector that introduces an emerald GFP (emGFP) reporter in-frame and a puromycin-resistance cassette. Successful homologous recombination resulted in both heterozygous ($GATA6^{GFP/+}$) and homozygous ($GATA6^{GFP/GFP}$) mutant cells
(D) Immunofluorescence showing emGFP-expressing heterozygous $GATA6^{GFP/+}$ and homozygous $GATA6^{GFP/GFP}$ mutant cells on day 3 of differentiation. Scale bars, 100 μm.
(E) PCR showing loss of transgenes in a patient A mutant hiPSC line, clone 1, compared with positive controls. Data are representative of three independent clones derived from either patient A or patient B.
(F and G) Genotype confirmation by Sanger sequencing of two *GATA6* patient-derived hiPSC lines: (F) patient A, $GATA6^{R465C/+}$, and (G) patient B, $GATA6^{c.1136-2A>G/+}$.
(H) Immunofluorescence confirming the successful reprogramming and pluripotency of one patient A-derived ($GATA6^{R465C/+}$) mutant line. Scale bars, 200 μm. Images are representative of three independent clones derived from either patient A or patient B ($GATA6^{c.1136-2A>G/+}$).
(I) Western blot analysis of GATA6 and GATA4 protein levels in undifferentiated H9*, $GATA6^{4ins/+}$, $GATA6^{\Delta4/\Delta4}$, $GATA6^{GFP/+}$, and $GATA6^{GFP/GFP}$ mutant cells, as well as the two patient-derived mutant lines: patient A, $GATA6^{R465C/+}$, and patient B, $GATA6^{c.1136-2A>G/+}$. α-tubulin was used as a loading control. Long and short isoforms of wild-type GATA6 are 60 and 45 kDa, respectively; the partial protein products for $GATA6^{4ins/+}$ are 30 and 18 kDa for the long and short isoforms, respectively; the partial protein products for $GATA6^{\Delta4/\Delta4}$ are 27 and 15 kDa for the long and short isoforms, respectively. No GATA6 protein was present for the $GATA6^{GFP/GFP}$ mutant.

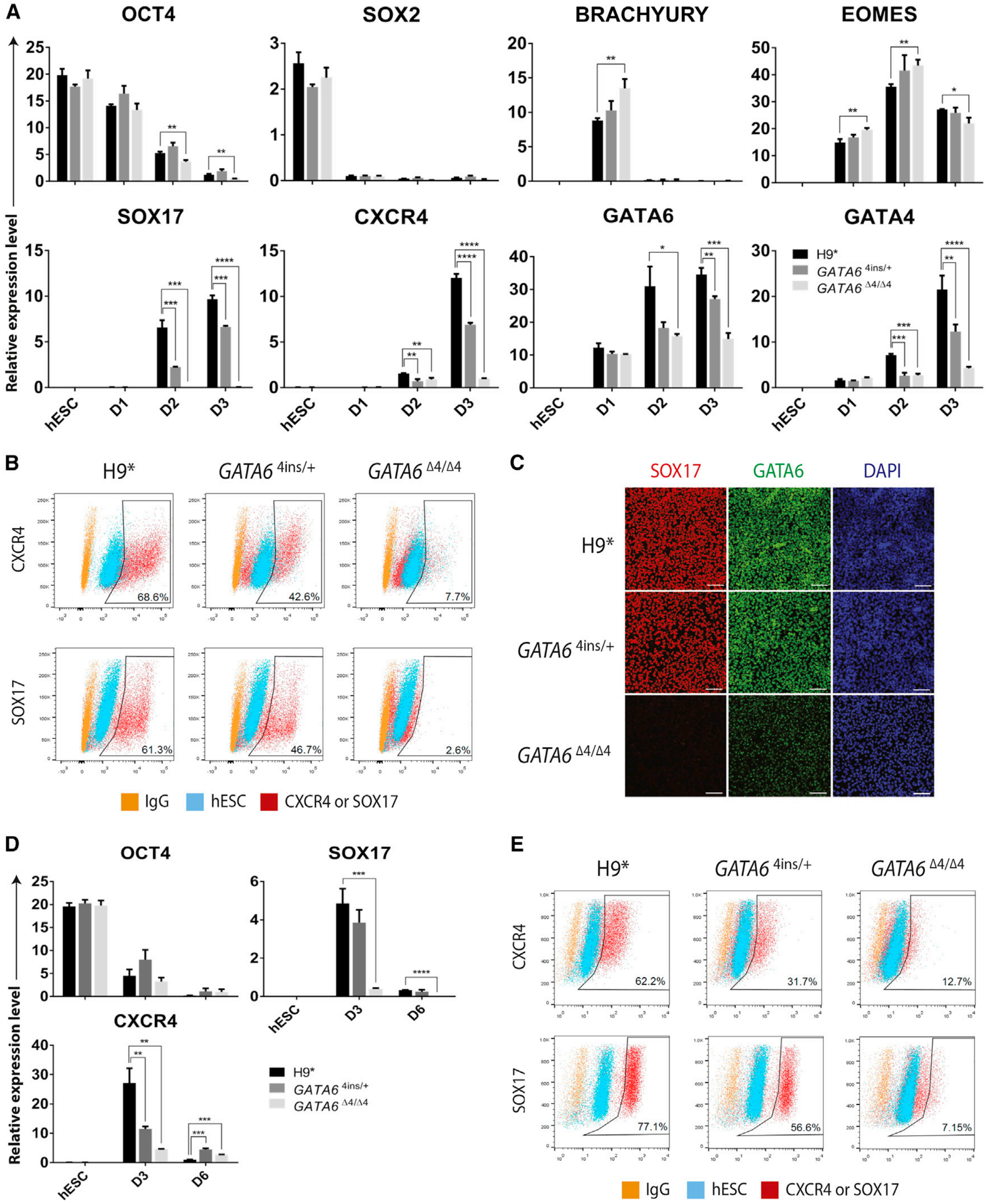

Figure 2. *GATA6*$^{4ins/+}$ and *GATA6*$^{\Delta4/\Delta4}$ Mutant hESC Lines Display Impaired DE Formation

(A) Expression of pluripotency (*OCT4*, *SOX2*), primitive streak (*BRACHYURY*), mesendoderm (*EOMES*), and definitive endoderm (*CXCR4*, *SOX17*, *GATA4*) markers, as well as *GATA6* itself, in H9* and H9-derived *GATA6*$^{4ins/+}$ and *GATA6*$^{\Delta4/\Delta4}$ mutant cells over 3 days of differentiation (Figure S1A).

(B) Differentiation efficiency measured by FACS analysis of CXCR4 and SOX17 at day 3 DE in H9* and H9-derived *GATA6*$^{4ins/+}$ and *GATA6*$^{\Delta4/\Delta4}$ mutant cells.

(C) Immunofluorescence analyses for the key DE markers GATA6 with SOX17 in H9* and H9-derived *GATA6*$^{4ins/+}$ and *GATA6*$^{\Delta4/\Delta4}$ mutant cells. DAPI, 4′,6-diamidino-2-phenylindole. Scale bars, 100 μm.

(legend continued on next page)

protein products predicted to contain 205 and 203 N-terminal amino acids, respectively, of the longer GATA6 isoform as well as additional novel C-terminal sequences (Figures 1A and 1I) that terminate before the two C-terminal zinc-finger DNA-binding domains. The insertion of the GFP reporter and puromycin-resistance cassettes in *GATA6* exon 2 generates a loss-of-function allele, since neither wild-type GATA6 isoform nor novel partial protein products were observed in *GATA6*$^{GFP/GFP}$ knockin H9 cells (Figures 1C and 1I).

Using the H9-derived *GATA6*$^{4ins/+}$ and *GATA6*$^{\Delta4/\Delta4}$ lines, we next asked whether reduced levels of GATA6 have an impact on early mesendoderm (corresponding to days 1 and 2) to DE (day 3) differentiation. qRT-PCR analyses show that in H9*, *GATA6*$^{4ins/+}$, and *GATA6*$^{\Delta4/\Delta4}$ cells, the levels of the pluripotency markers *OCT4* and *SOX2* were comparable in undifferentiated cells and expectedly declined during differentiation (Figure 2A). The expression of primitive streak (*BRACHYURY*) and mesendoderm (*EOMESODERMIN* [*EOMES*]) markers was also relatively unchanged across the control H9* and *GATA6* mutant lines, suggesting that early mesendoderm formation was not affected by either single or biallelic loss of *GATA6* (Figure 2A). Key DE markers *SOX17* and *CXCR4* were, however, modestly downregulated beginning on day 2 in *GATA6*$^{4ins/+}$ cells (Figure 2A), and on day 3, *GATA6*$^{4ins/+}$ differentiations yielded roughly 25% fewer SOX17^{+} cells by fluorescence-activated cell sorting (FACS) and IF compared with wild-type H9* (Figures 2B and 2C). This heterozygous effect on *SOX17* transcription was also observed to varying degrees in H9-*GATA6*$^{GFP/+}$ and 13.B-derived *GATA6*$^{\Delta14/+}$ as well as in patients A (*GATA6*$^{R456C/+}$) and B (*GATA6*$^{c.1136-2A>G/+}$) (Figures S2D–S2F). Interestingly, this heterozygous effect was not observed in 13.B-derived *GATA6*$^{GFP/+}$ (Figure S2E). Further depleting GATA6 with homozygous (*GATA6*$^{\Delta4/\Delta4}$ or *GATA6*$^{GFP/GFP}$) or compound heterozygous (13.B-*GATA6*$^{\Delta14/\Delta11}$) allelic combinations dramatically affects DE formation, yielding ~3% SOX17^{+} cells on day 3 (Figure 2B and data not shown). We further validated these results using the commercially available STEMdiff pancreatic progenitor kit from STEMCELL Technologies. Using this differentiation platform, H9*, *GATA6*$^{4ins/+}$, and *GATA6*$^{\Delta4/\Delta4}$ formed DE at efficiencies indistinguishable from the results obtained with the protocol outlined in Figure 1A (cf. Figures 2A and 2B with 2D and 2E). Taken together, these findings show that diminished levels of GATA6 compromise early DE formation, and complete loss of GATA6 significantly perturbs the gene regulatory network (GRN) governing human DE specification.

Establishing the GATA6 Gene Regulatory Network

To establish comprehensively how *GATA6* mutations alter the DE transcriptional network, we performed RNA sequencing (RNA-seq) for H9*, *GATA6*$^{4ins/+}$ and *GATA6*$^{\Delta4/\Delta4}$, and patient A cells on day 3 of differentiation. Comparative analyses revealed 7,472 genes that are differentially expressed (adjusted $p \leq 0.01$; fold change ≥ 2) between H9* and *GATA6*$^{\Delta4/\Delta4}$, 2,898 genes between H9* and *GATA6*$^{4ins/+}$, and 6,977 genes between H9* and hiPSC clones 1 to 3 from patient A (Table S1). We observed that, consistent with our qRT-PCR data in Figure 2, *GATA6*$^{\Delta4/\Delta4}$ mutant cells show significantly decreased expression of cardinal endoderm markers (e.g., *SOX17*, *CXCR4*, *HNF1B*, and *FOXA2*) (Figure 3A). Similar results were observed when wild-type H9* was compared with *GATA6*$^{4ins/+}$ and hiPSC clones 1 to 3 from patient A (Figures 3A and S3A).

We also performed GATA6 chromatin immunoprecipitation followed by high-throughput sequencing (ChIP-seq) on H9* and *GATA6*$^{4ins/+}$ cells at the DE stage. This analysis yielded 12,098 peaks (irreproducible discovery rate ≤ 0.05; median peak length = 417 bp) that are associated with 10,669 genes, 4,790 of which are protein coding (Table S1). Interestingly, we observe that GATA6 binding is enriched at the *GATA4* locus in H9 cells, suggesting that GATA6 directly regulates *GATA4* during DE specification (Figure 3B). Both qRT-PCR and RNA-seq show dose-dependent effects of GATA6 on *GATA4* expression levels in *GATA6*$^{4ins/+}$ and *GATA6*$^{\Delta4/\Delta4}$ mutant cells (Figures 2A, 3A, and 3B).

We next compared our RNA-seq and ChIP-seq datasets to identify those genes bound and regulated by GATA6. This analysis revealed 1,120 protein-coding genes that are bound by GATA6 in wild-type H9* but are downregulated in *GATA6*$^{\Delta4/\Delta4}$ mutant cells, including pancreatic progenitor genes such as *HNF1B* and *HNF4A* (Figure 3C). In contrast, 745 genes are bound by GATA6 in H9* and upregulated in *GATA6*$^{\Delta4/\Delta4}$. Similar overlaps were performed for *GATA6*$^{4ins/+}$ and patient A day 3 RNA-seq samples, yielding 337 and 607 GATA6-bound and downregulated genes, and

(D) Expression of pluripotency (*OCT4*) and definitive endoderm (*SOX17*, *CXCR4*) markers in H9* and H9-derived *GATA6*$^{4ins/+}$ and *GATA6*$^{\Delta4/\Delta4}$ mutant cells on days 3 and 6 of differentiation with the STEMdiff pancreatic progenitor kit.
(E) Differentiation efficiency measured by FACS analysis of CXCR4 and SOX17 at day 3 DE in H9* and H9-derived *GATA6*$^{4ins/+}$ and *GATA6*$^{\Delta4/\Delta4}$ mutant cells differentiated using the STEMdiff pancreatic progenitor kit.
(A and D) Error bars represent the SE of three independent experiments. *$p < 0.05$, **$p < 0.01$, ***$p < 0.001$, ****$p < 0.0001$.
(B and E) Undifferentiated hESCs stained with the respective primary and secondary antibodies and secondary antibody only (IgG) were both used as controls. Gates were set according to an hESC control.

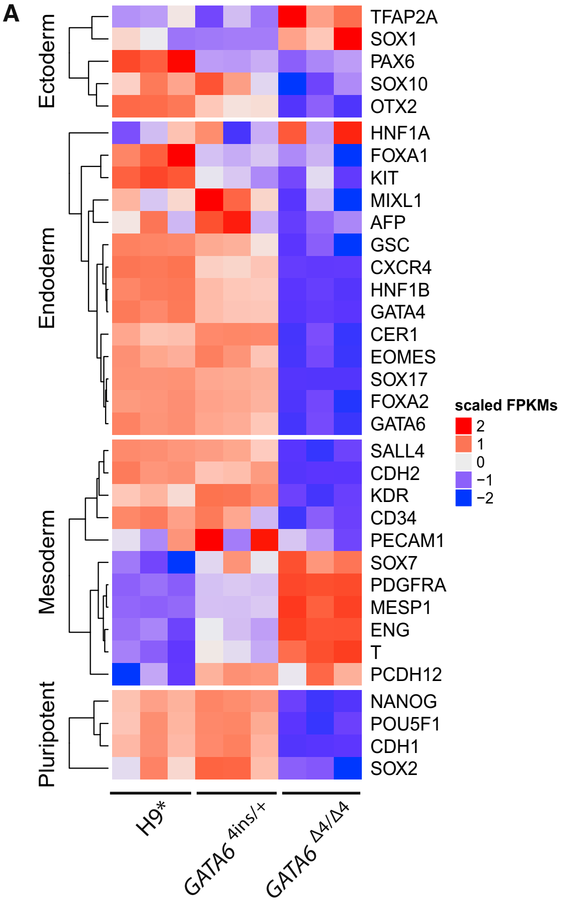

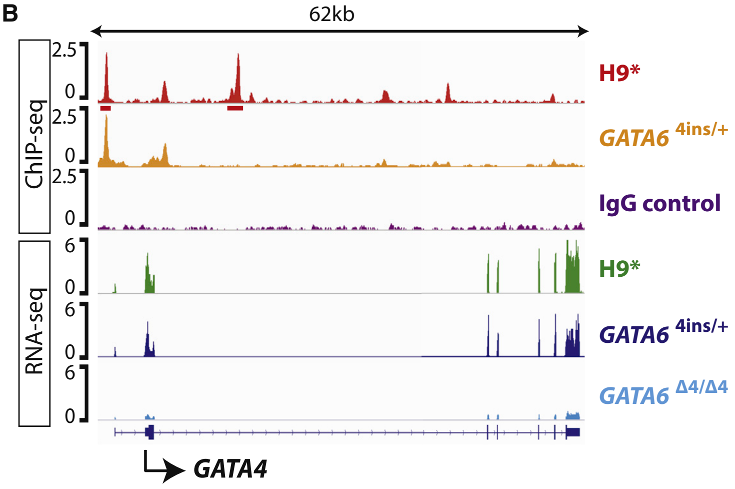

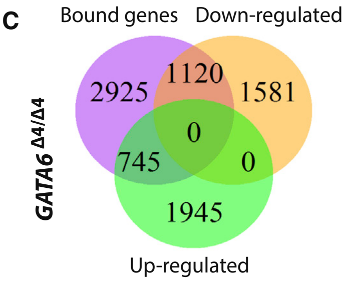

Genes bound and down-regulated	*SOX17, CXCR4, GATA4, GATA6, HNF4A, HNF1B, GSC, EOMES, FOXA2, KLF5, PRDM1*
Genes bound and up-regulated	*GATA5, RUNX1, RUNX2, FOXP1, EBF2, TWIST1, TSHZ2, TLE4*

D

Gene Ontology of genes bound and differenitally expressed in *GATA6* $^{\Delta4/\Delta4}$

Category	P-value	Gene symbol
**Up-regulated in WT**		
Endoderm development	8.74E-04	GDF3, COL4A2, HNF1B, FGF8, NODAL, EOMES, SMAD2, VTN, MMP15, HMGA2, HSBP1, COL5A2, MIXL1, DUSP5, HHEX, DUSP1, GATA6
Pancreas development	0.03667	INSM1, HNF1B, FOXA2, WFS1, SOX4, SMAD2, BAD, ISL1, TCF7L2, HHEX, BAK1, ACVR2B, MEIS2, GATA6, EIF2AK3, BMP5, BMP6
**Up-regulated in *GATA6* $^{\Delta4/\Delta4}$**		
Skeletal system development	2.66E-06	THRA, NDST1, HEXB, EDN1, TGFB3, BCAN, GJA5, TGFB2, MBTD1, INSIG2, PAX7, TRIM45, CHST11, RAB23, PHOSPHO1, HHIP, ANO6, ALX1, COL10A1
Mesoderm formation	0.001386	FGFR2, SIX2, SMAD3, ITGA3, WLS, PAX2, SNAI1, WNT3, DKK1, HAND1, SFRP2, ITGA8, EPB41L5, FOXC1, TLX2, ACVR1

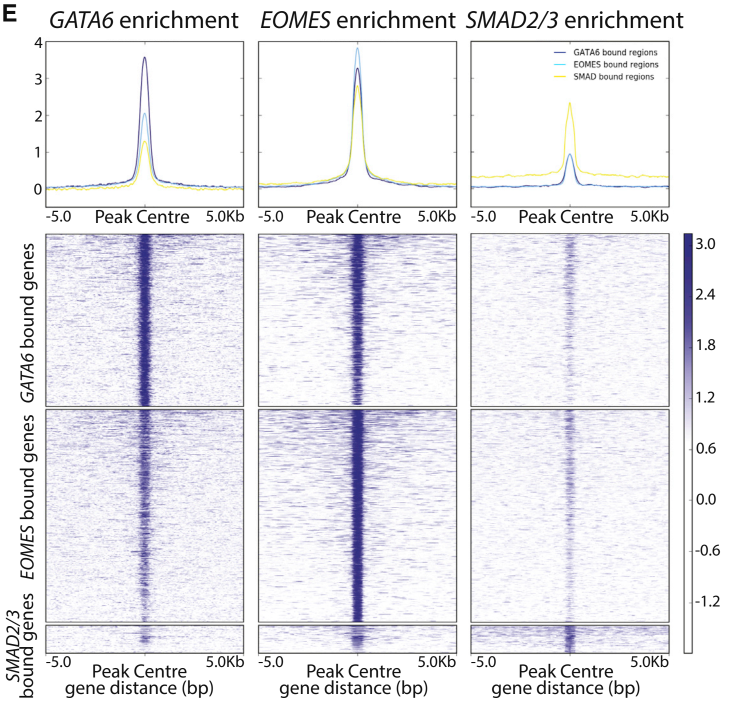

Figure 3. *GATA6* Is a Key Regulator of the DE Transcriptional Network

(A) Heatmap illustrating differential gene expression of key germ layer markers via RNA-seq between H9* cells and H9-derived *GATA6* $^{4ins/+}$ and *GATA6* $^{\Delta4/\Delta4}$ mutant cells at the DE stage. n = 3 biological replicates for each cell line.

(legend continued on next page)

254 and 616 GATA6-bound and upregulated genes, respectively (Figures S3B and S3C). At the intersection of these comparisons are 143 commonly downregulated and 104 upregulated genes among *GATA6*$^{\Delta4/\Delta4}$, *GATA6*$^{4ins/+}$, and patient A samples (Figures S3D and S3E). Key endoderm markers *CXCR4*, *SOX17*, *GATA4*, *HNF1B*, and *HNF4A* were among the 143 genes commonly downregulated (Figure S3D).

To infer genes that are directly targeted and regulated by GATA6, we performed binding and expression target analysis (BETA) to integrate our H9 ChIP-seq dataset with differential gene expression data from *GATA6*$^{\Delta4/\Delta4}$, *GATA6*$^{4ins/+}$ and patient A (Wang et al., 2013). Targets predicted by BETA were then subjected to gene ontology analyses using the DAVID tool (Huang da et al., 2009a, 2009b). We found that endoderm development is consistently upregulated in H9* compared with *GATA6*$^{\Delta4/\Delta4}$ (Figure 3D), *GATA6*$^{4ins/+}$ (Figure S3F), and patient A (Figure S3G) mutant cells. In addition, mesoderm development is consistently upregulated in *GATA6*$^{\Delta4/\Delta4}$ (Figure 3D), *GATA6*$^{4ins/+}$ (Figure S3F), and patient A (Figure S3G) mutant cells compared with H9*. Motif analyses generated by BETA confirm that the GATA recognition motif is highly enriched in both "up" and "down" target genes (Figure S3H). We were unable to conclude from the BETA whether GATA6 has activating or repressive functions, or both, as the data were not significant. Thus, we propose that the most parsimonious explanation for the upregulation of mesodermal markers is aberrant differentiation. In the absence of *GATA6*, differentiation into the DE lineage is blocked, but differentiating cells remain bathed in high levels of two potent mesoderm inducers (Activin and BMP4) (Cho et al., 2012). Taken together, these results show that *GATA6* is indispensable in driving the development of the human DE.

We previously established that the T-box transcription factor EOMES interacts with the Activin/Nodal effector proteins SMAD2/3 to deploy the GRN that directs DE formation. We thus sought to establish how GATA6 integrates into the SMAD2/3/EOMES signaling network by comparing our GATA6 day 3 ChIP-seq data with previously published SMAD2/3 and EOMES ChIP-seq (Brown et al., 2011; Teo et al., 2011). Remapping of the data resulted in 16,303, 20,089, and 2,613 peaks for GATA6, EOMES, and SMAD2/3, respectively. Of the 16,303 GATA6 ChIP-seq peaks, 950/2,613 (36.4%) overlap with SMAD2/3, and 8,126/20,089 (40.5%) overlap with EOMES in DE cells, with 858 common to all three datasets (Figure 3E, Table S1). In the EOMES/GATA6/SMAD2/3 intersection, we find almost all of the telltale endodermal regulator genes, including *SOX17*, *EOMES*, *LHX1*, *MIXL1*, *FOXA2*, *HNF1B*, and *CXCR4*. These data therefore place GATA6 centrally within the core nuclear transcriptional machinery that governs the acquisition of DE fate.

GATA6 Deficiency Impairs Pancreatic Lineage Commitment

We further analyzed the effects of *GATA6* heterozygous mutations on pancreatic lineage commitment at the PE (day 12) and endocrine progenitor (EP) (day 24) stages (Figure S1A). Key pancreatic markers such as *HNF4A*, *HLXB9*, *PDX1*, and *INSULIN* are significantly downregulated in *GATA6*$^{4ins/+}$ and *GATA6*$^{GFP/+}$ mutant cells at both stages, with one exception: *HLXB9* levels in *GATA6*$^{GFP/+}$ are no different from those in H9* on day 24 (Figure 4A). *HNF4A*, *PDX1*, and *INSULIN* were also significantly decreased in 13.B-*GATA6*$^{\Delta14/+}$ and 13.B-*GATA6*$^{GFP/+}$ and in patient A and B mutant cells on days 12 and 24 (Figures S4A and S4B). FACS analysis for PDX1 on day 12 reveals an approximately 50% decrease in the number of PDX1-positive *GATA6*$^{4ins/+}$ and *GATA6*$^{GFP/+}$ cells and 13.B-*GATA6*$^{GFP/+}$ cells (Figures 4B and S4C). 13.B-*GATA6*$^{\Delta14/+}$, patient A, and patient B cell lines exhibit an approximately 80%–90% decrease in PDX1 (Figures S4C and S4D). At the EP stage, all *GATA6* heterozygous mutant cell lines share a common phenotype, with a strong decrease in the number of C-PEPTIDE^{+} cells (Figure 4C, S4C, and S4D). Immunostaining on H9* and *GATA6*$^{4ins/+}$ cells confirms the diminished number of SOMATOSTATIN-, C-PEPTIDE-, and GLUCAGON-positive cells in *GATA6*$^{4ins/+}$ cells (Figure 4D).

We also performed RNA-seq at the PE stage (day 12) for H9*, *GATA6*$^{4ins/+}$, and patient A cells. H9* RNA-seq largely reproduced a previous dataset generated using both H9 and the same differentiation protocol (Spearman's rank correlation coefficient, $\rho = 0.77$ for *in vitro* multipotent

(B) ChIP-seq binding profiles of H9* and *GATA6*$^{4ins/+}$ showing *GATA6* enrichment near *GATA4*, and *GATA4* expression by RNA-seq in H9* and H9-derived *GATA6*$^{4ins/+}$ and *GATA6*$^{\Delta4/\Delta4}$ mutant cells at the DE stage. The input control profile (IgG control) is included for comparison. The ChIP-seq binding profile is derived from merging two biological replicates.

(C) Venn diagram indicating the overlap of GATA6-bound genes from ChIP-seq at the DE stage with downregulated or upregulated genes in H9-derived *GATA6*$^{\Delta4/\Delta4}$ mutant cells compared with H9* cells by RNA-seq. Key bound genes up- or downregulated are indicated in the table.

(D) Enriched gene ontology showing developmental pathways from direct target genes differentially expressed between H9* and H9-derived *GATA6*$^{\Delta4/\Delta4}$ mutant cells derived from BETA analysis.

(E) Density heatmaps of *GATA6*-binding peak intensity at DE indicating direct overlap with known endodermal regulators, including *SMAD2/3* and *EOMES*, within a 5-kb window centered at the transcription start site.

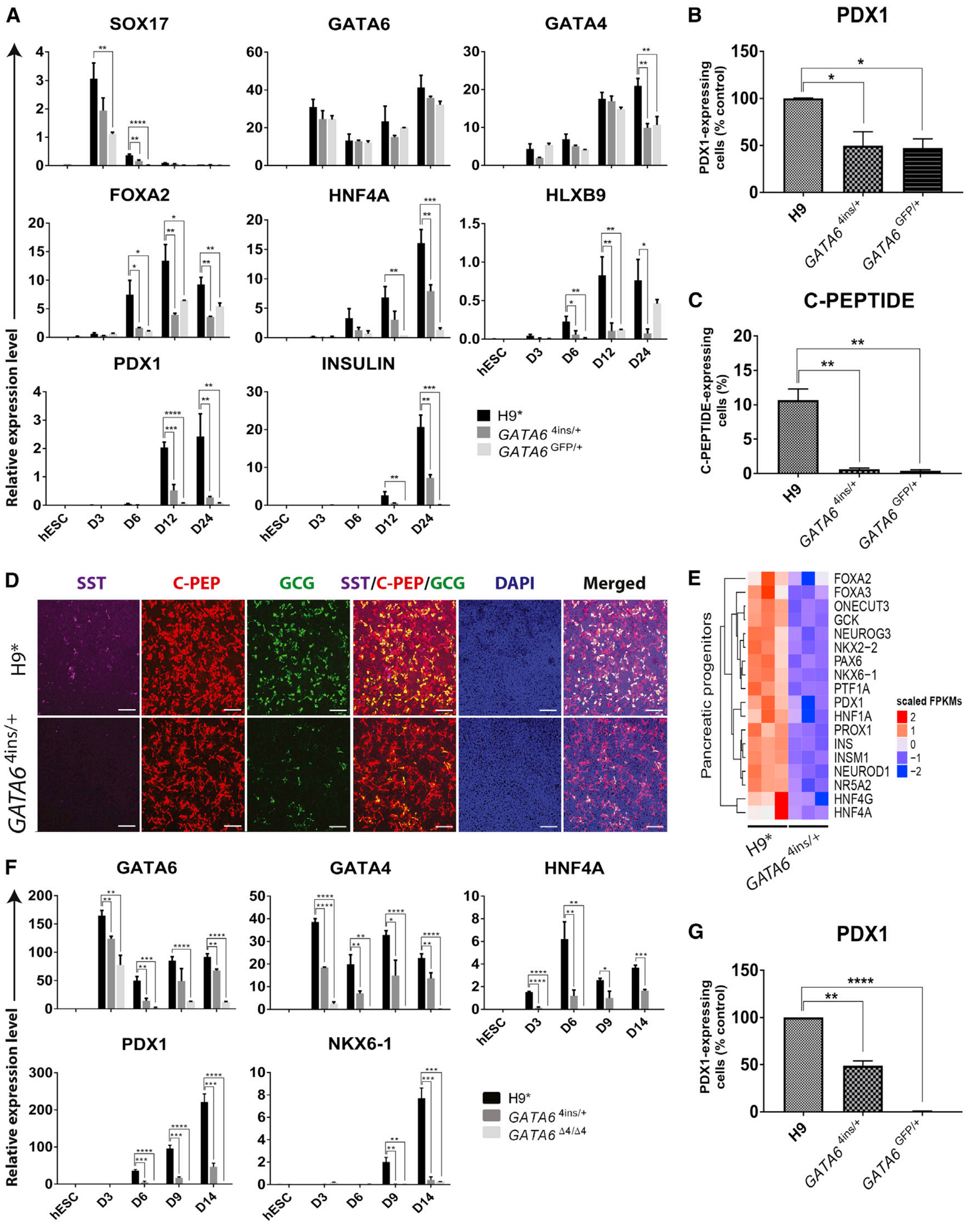

Figure 4. Decreased Levels of GATA6 at the DE Stage Influence Downstream Pancreatic Differentiation

(A) Expression of DE (*SOX17*, *GATA6*, *GATA4*, and *FOXA2*), pancreatic (*HNF4A*, *HLXB9*, and *PDX1*), and endocrine (*INSULIN*) marker genes in H9* and H9-derived *GATA6*$^{4ins/+}$ and *GATA6*$^{\Delta4/\Delta4}$ mutant cells at the four key stages of the 24-day pancreatic differentiation protocol (Figure S1A).

(B) Percentage of PDX1-positive cells in H9-derived *GATA6*$^{4ins/+}$ and *GATA6*$^{GFP/+}$ lines on day 12 shown relative to H9* (100%) as measured by FACS.

(C) Percentage of C-PEPTIDE-positive cells in H9* and H9-derived *GATA6*$^{4ins/+}$ and *GATA6*$^{GFP/+}$ mutant lines at the EP stage (day 24) as measured by FACS.

(legend continued on next page)

pancreatic progenitor cells [MPCs] and $\rho = 0.59$ for *in vivo* MPCs isolated from Carnegie stage 16–18 human embryos, $p < 2.2 \times 10^{-16}$) (Cebola et al., 2015). Between H9* and $GATA6^{4ins/+}$ (Table S1), 1,423 genes were differentially expressed, and between H9* and patient A, 6,093 were differentially expressed (Table S1). We observed that, consistent with qRT-PCR and FACS results, $GATA6^{4ins/+}$ and patient A gene expression quantified by RNA-seq in mutant cells displays a decreased pancreatic signature (Figures 4E and S4E).

Finally, the above results were independently confirmed with H9*, $GATA6^{4ins/+}$, and $GATA6^{\Delta4/\Delta4}$ cells using the STEMdiff pancreatic progenitor kit: $GATA6^{4ins/+}$ cells show decreased *PDX1* and *NKX6-1* expression beginning on day 9, yielding ~50% fewer $PDX1^{+}$ pancreatic progenitors on day 12 (Figures 4F and 4G). Collectively, these *in vitro* findings strongly argue that decreased levels of GATA6 first influence the formation of DE, and predict that with fewer DE cells at the time of allocation to the pancreatic lineage *in vivo*, hypoplasia emerges.

DISCUSSION

Deriving iPSC lines and the ability to rapidly engineer mutations in hPSCs have made human disease modeling *in vitro* commonplace. In the case of the well-characterized set of genes known to control mammalian pancreatic development, it is the expectation based on strong evolutionary conservation that phenotypes observed in knockout mouse models will be reproduced in human and *in vitro*. Indeed, the matching of human and mouse pancreatic and extrapancreatic phenotypes is seen for many recessive loss-of-function mutations in key pancreatic developmental transcription factors, e.g., PDX1, *PTF1A*, *RFX6*, *NEUROD1*, *NGN3*, and *NKX2-2* (Ahlgren et al., 1996; Flanagan et al., 2014; Jonsson et al., 1994; Offield et al., 1996; Rubio-Cabezas et al., 2010, 2011; Schwitzgebel et al., 2003; Sellick et al., 2004; Smith et al., 2010; Stoffers et al., 1997). Such consistency in phenotype is, however, not observed with *Gata6*. In mice, only the simultaneous inactivation of *Gata6* and *Gata4* results in pancreatic agenesis (Carrasco et al., 2012; Xuan et al., 2012), whereas in humans *de novo* heterozygous mutations in *GATA6* underlie a similar pathology (De Franco et al., 2013; Lango Allen et al., 2011). However, *GATA6* heterozygous phenotypes range from total pancreatic agenesis to isolated diabetes in young adulthood. This phenotypic diversity partly explains the difficulties in precisely modeling *GATA6* haploinsufficiency *in vitro* across laboratories and across differentiation platforms, as evidenced by comparing our present findings with two recent reports from Shi et al. (2017) and Tiyaboonchai et al. (2017).

Here, we find a modest reduction (~25%) in the production of DE after 3 days of directed differentiation irrespective of whether the *GATA6* heterozygous line was patient derived or generated by gene editing in hPSCs. These findings are consistent with *GATA6* expression in the DE, but contrast with the results of Shi et al. (2017) and Tiyaboonchai et al. (2017). These authors did not observe decreased DE formation using assorted *GATA6* heterozygous hPSC lines. One potential explanation for these discrepant results is that GATA6 partial protein products, generated, for example, from the $GATA6^{4ins/+}$ allele in H9 cells, act in our hands in a dominant negative manner, further suppressing *GATA6* levels and compromising normal DE formation (Figure 1I). The partial protein products encoded by the $GATA6^{4ins/+}$ locus are predicted to retain a long stretch of the N-terminal GATA6 transactivation domain but lack the zinc-finger DNA-binding domain and nuclear localization signal. As they are able neither to bind DNA nor to heterodimerize with GATA4 (Charron et al., 1999; Maeda et al., 2005), the biochemical mechanism by which such partial protein products interfere with *GATA6* transcription or function is entirely unclear. Moreover, Tiyaboonchai et al. (2017) and Shi et al. (2017) also observe partial protein products in their *GATA6* heterozygous hPSC lines, but do not observe effects during DE differentiation. The most significant evidence against dominant interference and in favor of a simple dosage effect comes from the fact that patient A and B iPSCs, whose mutations do not result in partial protein products (Figure 1I), also show decreased DE formation on day 3. Alternatively, because each group employed different hESC and iPSC lines, the specter of well-known line-to-line variations in differentiation efficiency could explain the results from the different laboratories (Cahan and Daley, 2013; Ortmann and Vallier, 2017).

(D) Immunofluorescence analyses for the key PE markers SOMATOSTATIN (SST), C-PEPTIDE (C-PEP), and GLUCAGON (GCG) in H9* and H9-derived $GATA6^{4ins/+}$ cells at the EP stage (day 24). DAPI, 4′,6-diamidino-2-phenylindole dihydrochloride. Scale bars, 100 μm.
(E) Heatmap illustrating differential gene expression of key pancreatic progenitor markers via RNA-seq between H9*cells and H9-derived $GATA6^{4ins/+}$ mutant cells at the PE stage. n = 3 biological replicates for each cell line.
(F) Expression of DE (*GATA6* and *GATA4*) and pancreatic (*HNF4A*, *PDX1*, and *NKX6-1*) marker genes in H9* and H9-derived $GATA6^{4ins/+}$ and $GATA6^{\Delta4/\Delta4}$ mutant cells at key stages of the differentiation protocol using the STEMdiff pancreatic progenitor kit.
(G) Percentage of PDX1-positive cells in $GATA6^{4ins/+}$ and $GATA6^{GFP/+}$ lines on day 12 shown relative to H9* (100%) as measured by FACS in cells differentiated using the STEMdiff pancreatic progenitor kit.
(A–C, F, and G) Error bars represent the SE of three independent experiments. *$p < 0.05$, **$p < 0.01$, ***$p < 0.001$, ****$p < 0.0001$.

Despite these differences among the *GATA6* heterozygous phenotypes at the DE stage, complete loss of GATA6 was found by Tiyaboonchai et al. (2017), Shi et al. (2017), and us (Figure 2), as well as more recently by Liao et al. (2018) with short hairpin RNA targeting *GATA6* in H1 cells, to unequivocally impair DE formation, a result highlighting not only the requirement for wild-type *GATA6* gene dosage for robust DE specification in humans but also the dramatic species-specific differences between mice and humans. Importantly, our genome-wide studies place GATA6 among the core transcriptional machinery that controls DE formation. We previously reported that the pluripotency factors OCT4, SOX2, and NANOG bind cooperatively and control the expression of the T-box transcription factor gene *EOMES* (Teo et al., 2011). Upon activation, EOMES, jointly with SMAD2/3, the intracellular effectors of ACTIVIN/NODAL signaling, deploys a large part of the transcriptional network governing DE formation. Here, we find 858 genes that are bound within 5 kb of the transcription start site by EOMES, SMAD2/3, and GATA6. Importantly, such cooperation has not been evidenced in mouse development, suggesting major divergences between species in the molecular mechanisms controlling germ-layer specification. Considering the importance of *GATA6* in specification of extraembryonic endoderm, this divergence in signaling pathways could result in the rewiring of downstream transcriptional networks with major consequences on the subsequent specification of DE.

With extended differentiation to the PE stage (day 12), we observe significantly decreased numbers of $PDX1^+$ cells—between 50% and 90% fewer compared with wild-type depending on the *GATA6* heterozygous line. This result is consistent with *GATA6* expression in human pancreatic progenitors (Figure S1C) and with GATA6 directly regulating *PDX1* transcription (Carrasco et al., 2012; Xuan et al., 2012) and also suggests that GATA6 plays a dual role in both early DE formation and allocation to the pancreatic lineage. The diminished numbers of *GATA6* heterozygous $PDX1^+$ progenitors that emerge at the PE stage ultimately differentiate into $\leq$10% $C\text{-}PEPTIDE^+$ cells by the EP stage (day 24), across all cell types and across all alleles.

It is tempting to consider that the variation in clinical phenotype and the early phenotype in DE formation *in vitro* might be predominantly attributable to individual genetic backgrounds (Chen et al., 2016; Lek et al., 2016). *GATA4* is an obvious choice for a genetic modifier, given its expression in the DE, genetic interaction with *Gata6* in mice, and the identification of rare *GATA4* heterozygous patients with pancreatic agenesis, as well as our finding that *GATA4* is bound and regulated by GATA6 *in vitro* (Figure 3) (D'Amato et al., 2010; Freyer et al., 2015; Morrisey et al., 1996; Shaw-Smith et al., 2014). Indeed, Shi et al. (2017) elegantly show dosage-dependent effects of *GATA4* alleles on phenotypes associated with *GATA6* heterozygosity during *in vitro* differentiation. Despite reports of considerable phenotypic variation between family members with the same *GATA6* mutation (Bonnefond et al., 2012; De Franco et al., 2013; Yau et al., 2017), in some cases a parent is a mosaic for the phenotype, so the variation between parental and offspring phenotypes can be explained by different mutation load in target tissues (Yau et al., 2017). If the variation in the human phenotype altered significantly with the genetic background, then most cases with severe pancreatic agenesis would likely be born to parents with the same mutation, but a 50% different (protective) genetic background would have a milder phenotype. However, this is not the case, as the vast majority of severe pancreatic agenesis is from *de novo* mutations (De Franco et al., 2013; Lango Allen et al., 2011). This means it is possible, but not likely, that genetic background explains why Shi et al. (2017) engineered, using CRISPR/Cas9, the common *GATA6* agenesis mutation c.1366C>T (p.Arg456Cys) in HUES8 cells—the same allele present in our patient A-derived iPSC line ($GATA6^{R456C/+}$)—and observed no heterozygous phenotype at the DE or pancreatic progenitor ($PDX1^+$) stages, whereas we do, at both the DE stage and beyond.

In addition to line-to-line differentiation efficiencies *in vitro* (Cahan and Daley, 2013; Ortmann and Vallier, 2017), fundamental differences in the differentiation protocols themselves may underlie (or contribute to) the results we report here and those published by Shi et al. (2017) and Tiyaboonchai et al. (2017). For example, the growth factor and small-molecule components as well as medium formulations differ substantially for the first 3 days of DE differentiation among the three studies. Furthermore, our differentiation protocol relies on culture media devoid of serum or complex extracellular matrices such as Matrigel. Thus, the minimalist approach of our system could exacerbate the *GATA6* phenotype, revealing a function for this gene that is otherwise masked. This possibility highlights the importance of culture conditions to study gene function in hPSCs and during their differentiation. Tiyaboonchai et al. (2017) additionally show that a *GATA6* heterozygous iPSC line derived from an agenesis patient unexpectedly produced β-like cells *in vitro*. Simply reducing the concentration of retinoic acid 80-fold led to statistically significantly fewer $PDX1^+$ cells compared with a wild-type iPSC line that showed negligible sensitivity to the same culture regime. Indeed, current hPSC pancreatic differentiation protocols have been highly tailored and refined, providing redundant and reinforcing signals that perhaps reconfigure underlying GRNs and bypass certain *in vivo* genetic requirements. Moreover, it must be acknowledged

that adherent differentiation fails to achieve the 3D complexity of human endoderm formation *in vivo*. Thus, studies of early pancreatic lineage commitment would greatly benefit from universal protocols standardized intra- and inter-laboratory in an effort to minimize line-to-line and protocol-to-protocol differences.

EXPERIMENTAL PROCEDURES

Human Pluripotent Stem Cell Culture and Pancreatic Differentiation

hESCs (H9 [WA09 from www.wicell.org]), hiPSCs (FSPS13.B derived in-house from human fibroblasts [http://www.hipsci.org/lines/#/lines/HPSI0813i-fpdm_2]), and *GATA6* patient-derived iPSCs, from patients A and B, were routinely cultured under feeder-free conditions on vitronectin-coated (STEMCELL Technologies #07180) tissue culture plates (Corning) with Essential 8 Medium (Life Technologies #A1517001). All tissue culture was carried out in 5% CO_2 at 37°C. Pancreatic differentiation was carried out as previously described (Cho et al., 2012), with modifications described in Supplemental Experimental Procedures.

GATA6 Patient Samples

The generation of *GATA6* patient-derived hiPSCs was approved by the Great Ormond Street Hospital and Institute of Child Health Research Ethics Committee (ethics reference: 08/H0713/82), and informed consent was obtained from all patients. Skin punch biopsy samples were collected from patients and all hiPSC lines used were derived and validated by the Cambridge Biomedical Research Center hiPSC Core Facility. Reprogramming of the *GATA6* patient fibroblasts to derive *GATA6* patient iPSCs was done by the hiPSC core facility at the Anne McLaren Laboratory for Regenerative Medicine using Sendai virus reprogramming.

GATA6-Mutant and *GATA6*-emGFP Reporter hPSC Derivation

The construction of TALEN vectors, their introduction into H9 or 13.B cells via electroporation, and the screening of drug-resistant clones are described in detail in the Supplemental Experimental Procedures. Two TALEN pairs were generated, each targeting a different site within *GATA6* exon 2. The first TALEN pair targets a site that is 6 bp downstream of the first *GATA6* start codon. The second targets a site that is 149 bp downstream of the second *GATA6* start codon. Primers used for TALEN construction and screening of genomic DNA are listed in Table S2.

RNA- and ChIP-Sequencing Analysis of Gene Expression

Library preparation and deep sequencing were performed at the Wellcome Trust Sanger Institute (Hinxton, UK). RNA-seq and ChIP-seq were run on Illumina Hiseq v.3 and v.4, respectively, with read length 75 bp and paired ends, and a library fragment size of 100–1,000 bp using a multiplex strategy. RNA-seq and ChIP-seq samples were run in biological triplicates and duplicates, respectively. Additional details of how RNA-seq and ChIP-seq reads were aligned and analyzed can be found in the Supplemental Experimental Procedures.

ACCESSION NUMBERS

Primary RNA-seq and ChIP-seq datasets generated here are available at ArrayExpress under accession numbers ArrayExpress: E-MTAB-5958 and E-MTAB-5959, respectively.

SUPPLEMENTAL INFORMATION

Supplemental Information includes Supplemental Experimental Procedures, four figures, and four tables and can be found with this article online at https://doi.org/10.1016/j.stemcr.2018.12.003.

AUTHOR CONTRIBUTIONS

L.V., N.R.D., C.Y.C., and M.C. designed the methods and experiments. C.Y.C. performed the experiments. C.Y.C., P.M., and S.L.I.J.D performed bioinformatics analyses. I.M. and J.G.-B. performed additional differentiation experiments for the revisions. A.T.H. and M.H.S identified the patients and designed the patient sample collection. M.H.S. and R.E.K. collected the patient samples. C.Y.C. and N.R.D. wrote the first draft. All authors approved the final draft and made modifications to the text.

ACKNOWLEDGMENTS

We thank the NIHR Cambridge BRC hiPSC core facility for the derivation of *GATA6* hiPSC lines, Dr. Norihiro Tsuneyoshi for advice and guidance on gene editing using TALENs, and Dr. Bruno Reversade for insightful discussions. This work was funded by an EDB Singapore Childhood Undiagnosed Diseases Program grant and an A*STAR Strategic Positioning Fund (SPF) Genetic Orphan Diseases Adopted: Fostering Innovation Therapy (GODAFIT) grant and by an ERC starting grant Relieve IMDs and core grant funding from the Wellcome Trust and Medical Research Council (PSAG028) (L.V. and P.M.). A.T.H. is a Wellcome Trust Senior Investigator.

Received: September 12, 2017
Revised: December 9, 2018
Accepted: December 10, 2018
Published: January 8, 2019

REFERENCES

Ahlgren, U., Jonsson, J., and Edlund, H. (1996). The morphogenesis of the pancreatic mesenchyme is uncoupled from that of the pancreatic epithelium in IPF1/PDX1-deficient mice. Development *122*, 1409–1416.

Bonnefond, A., Sand, O., Guerin, B., Durand, E., De Graeve, F., Huyvaert, M., Rachdi, L., Kerr-Conte, J., Pattou, F., Vaxillaire, M., et al. (2012). GATA6 inactivating mutations are associated with heart defects and, inconsistently, with pancreatic agenesis and diabetes. Diabetologia *55*, 2845–2847.

Brewer, A., Gove, C., Davies, A., McNulty, C., Barrow, D., Koutsourakis, M., Farzaneh, F., Pizzey, J., Bomford, A., and Patient, R. (1999).

The human and mouse GATA-6 genes utilize two promoters and two initiation codons. J. Biol. Chem. *274*, 38004–38016.

Brown, S., Teo, A., Pauklin, S., Hannan, N., Cho, C.H., Lim, B., Vardy, L., Dunn, N.R., Trotter, M., Pedersen, R., et al. (2011). Activin/Nodal signaling controls divergent transcriptional networks in human embryonic stem cells and in endoderm progenitors. Stem Cells *29*, 1176–1185.

Cahan, P., and Daley, G.Q. (2013). Origins and implications of pluripotent stem cell variability and heterogeneity. Nat. Rev. Mol. Cell Biol. *14*, 357–368.

Carrasco, M., Delgado, I., Soria, B., Martin, F., and Rojas, A. (2012). GATA4 and GATA6 control mouse pancreas organogenesis. J. Clin. Invest. *122*, 3504–3515.

Cebola, I., Rodriguez-Segui, S.A., Cho, C.H., Bessa, J., Rovira, M., Luengo, M., Chhatriwala, M., Berry, A., Ponsa-Cobas, J., Maestro, M.A., et al. (2015). TEAD and YAP regulate the enhancer network of human embryonic pancreatic progenitors. Nat. Cell Biol. *17*, 615–626.

Chao, C.S., McKnight, K.D., Cox, K.L., Chang, A.L., Kim, S.K., and Feldman, B.J. (2015). Novel GATA6 mutations in patients with pancreatic agenesis and congenital heart malformations. PLoS One *10*, e0118449.

Charron, F., Paradis, P., Bronchain, O., Nemer, G., and Nemer, M. (1999). Cooperative interaction between GATA-4 and GATA-6 regulates myocardial gene expression. Mol. Cell. Biol. *19*, 4355–4365.

Chen, R., Shi, L., Hakenberg, J., Naughton, B., Sklar, P., Zhang, J., Zhou, H., Tian, L., Prakash, O., Lemire, M., et al. (2016). Analysis of 589,306 genomes identifies individuals resilient to severe Mendelian childhood diseases. Nat. Biotechnol. *34*, 531–538.

Cho, C.H., Hannan, N.R., Docherty, F.M., Docherty, H.M., Joao Lima, M., Trotter, M.W., Docherty, K., and Vallier, L. (2012). Inhibition of activin/nodal signalling is necessary for pancreatic differentiation of human pluripotent stem cells. Diabetologia *55*, 3284–3295.

D'Amato, E., Giacopelli, F., Giannattasio, A., D'Annunzio, G., Bocciardi, R., Musso, M., Lorini, R., and Ravazzolo, R. (2010). Genetic investigation in an Italian child with an unusual association of atrial septal defect, attributable to a new familial GATA4 gene mutation, and neonatal diabetes due to pancreatic agenesis. Diabet. Med. *27*, 1195–1200.

Decker, K., Goldman, D.C., Grasch, C.L., and Sussel, L. (2006). Gata6 is an important regulator of mouse pancreas development. Dev. Biol. *298*, 415–429.

Flanagan, S.E., De Franco, E., Lango Allen, H., Zerah, M., Abdul-Rasoul, M.M., Edge, J.A., Stewart, H., Alamiri, E., Hussain, K., Wallis, S., et al. (2014). Analysis of transcription factors key for mouse pancreatic development establishes NKX2-2 and MNX1 mutations as causes of neonatal diabetes in man. Cell Metab. *19*, 146–154.

De Franco, E., Shaw-Smith, C., Flanagan, S.E., Shepherd, M.H., International, N.D.M.C., Hattersley, A.T., and Ellard, S. (2013). GATA6 mutations cause a broad phenotypic spectrum of diabetes from pancreatic agenesis to adult-onset diabetes without exocrine insufficiency. Diabetes *62*, 993–997.

Freyer, L., Schroter, C., Saiz, N., Schrode, N., Nowotschin, S., Martinez-Arias, A., and Hadjantonakis, A.K. (2015). A loss-of-function and H2B-Venus transcriptional reporter allele for Gata6 in mice. BMC Dev. Biol. *15*, 38.

Huang da, W., Sherman, B.T., and Lempicki, R.A. (2009a). Bioinformatics enrichment tools: paths toward the comprehensive functional analysis of large gene lists. Nucleic Acids Res. *37*, 1–13.

Huang da, W., Sherman, B.T., and Lempicki, R.A. (2009b). Systematic and integrative analysis of large gene lists using DAVID bioinformatics resources. Nat. Protoc. *4*, 44–57.

Jennings, R.E., Berry, A.A., Kirkwood-Wilson, R., Roberts, N.A., Hearn, T., Salisbury, R.J., Blaylock, J., Piper Hanley, K., and Hanley, N.A. (2013). Development of the human pancreas from foregut to endocrine commitment. Diabetes *62*, 3514–3522.

Jonsson, J., Carlsson, L., Edlund, T., and Edlund, H. (1994). Insulin-promoter-factor 1 is required for pancreas development in mice. Nature *371*, 606–609.

Jørgensen, M.C., Ahnfelt-Rønne, J., Hald, J., Madsen, O.D., Serup, P., and Hecksher-Sørensen, J. (2007). An illustrated review of early pancreas development in the mouse. Endocr. Rev. *28*, 685–705.

Ketola, I., Otonkoski, T., Pulkkinen, M.A., Niemi, H., Palgi, J., Jacobsen, C.M., Wilson, D.B., and Heikinheimo, M. (2004). Transcription factor GATA-6 is expressed in the endocrine and GATA-4 in the exocrine pancreas. Mol. Cell. Endocrinol. *226*, 51–57.

Koutsourakis, M., Langeveld, A., Patient, R., Beddington, R., and Grosveld, F. (1999). The transcription factor GATA6 is essential for early extraembryonic development. Development *126*, 723–732.

Lango Allen, H., Flanagan, S.E., Shaw-Smith, C., De Franco, E., Akerman, I., Caswell, R., International Pancreatic Agenesis, C., Ferrer, J., Hattersley, A.T., and Ellard, S. (2011). GATA6 haploinsufficiency causes pancreatic agenesis in humans. Nat. Genet. *44*, 20–22.

Lek, M., Karczewski, K.J., Minikel, E.V., Samocha, K.E., Banks, E., Fennell, T., O'Donnell-Luria, A.H., Ware, J.S., Hill, A.J., Cummings, B.B., et al. (2016). Analysis of protein-coding genetic variation in 60,706 humans. Nature *536*, 285–291.

Lentjes, M.H., Niessen, H.E., Akiyama, Y., de Bruine, A.P., Melotte, V., and van Engeland, M. (2016). The emerging role of GATA transcription factors in development and disease. Expert Rev. Mol. Med. *18*, e3.

Liao, C.M., Mukherjee, S., Tiyaboonchai, A., Maguire, J.A., Cardenas-Diaz, F.L., French, D.L., and Gadue, P. (2018). GATA6 suppression enhances lung specification from human pluripotent stem cells. J. Clin. Invest. *128*, 2944–2950.

Maeda, M., Ohashi, K., and Ohashi-Kobayashi, A. (2005). Further extension of mammalian GATA-6. Dev. Growth Differ. *47*, 591–600.

Morrisey, E.E., Ip, H.S., Lu, M.M., and Parmacek, M.S. (1996). GATA-6: a zinc finger transcription factor that is expressed in multiple cell lineages derived from lateral mesoderm. Dev. Biol. *177*, 309–322.

Morrisey, E.E., Tang, Z., Sigrist, K., Lu, M.M., Jiang, F., Ip, H.S., and Parmacek, M.S. (1998). GATA6 regulates HNF4 and is required for differentiation of visceral endoderm in the mouse embryo. Genes Dev. *12*, 3579–3590.

Offield, M.F., Jetton, T.L., Labosky, P.A., Ray, M., Stein, R.W., Magnuson, M.A., Hogan, B.L., and Wright, C.V. (1996). PDX-1 is required for pancreatic outgrowth and differentiation of the rostral duodenum. Development *122*, 983–995.

Ortmann, D., and Vallier, L. (2017). Variability of human pluripotent stem cell lines. Curr. Opin. Genet. Dev. *46*, 179–185.

Pan, F.C., and Wright, C. (2011). Pancreas organogenesis: from bud to plexus to gland. Dev. Dyn. *240*, 530–565.

Patient, R.K., and McGhee, J.D. (2002). The GATA family (vertebrates and invertebrates). Curr. Opin. Genet. Dev. *12*, 416–422.

Rubio-Cabezas, O., Minton, J.A., Kantor, I., Williams, D., Ellard, S., and Hattersley, A.T. (2010). Homozygous mutations in NEUROD1 are responsible for a novel syndrome of permanent neonatal diabetes and neurological abnormalities. Diabetes *59*, 2326–2331.

Rubio-Cabezas, O., Jensen, J.N., Hodgson, M.I., Codner, E., Ellard, S., Serup, P., and Hattersley, A.T. (2011). Permanent neonatal diabetes and enteric anendocrinosis associated with biallelic mutations in NEUROG3. Diabetes *60*, 1349–1353.

Schrode, N., Saiz, N., Di Talia, S., and Hadjantonakis, A.K. (2014). GATA6 levels modulate primitive endoderm cell fate choice and timing in the mouse blastocyst. Dev. Cell *29*, 454–467.

Schwitzgebel, V.M. (2014). Many faces of monogenic diabetes. J. Diabetes Investig. *5*, 121–133.

Schwitzgebel, V.M., Mamin, A., Brun, T., Ritz-Laser, B., Zaiko, M., Maret, A., Jornayvaz, F.R., Theintz, G.E., Michielin, O., Melloul, D., et al. (2003). Agenesis of human pancreas due to decreased half-life of insulin promoter factor 1. J. Clin. Endocrinol. Metab. *88*, 4398–4406.

Sellick, G.S., Barker, K.T., Stolte-Dijkstra, I., Fleischmann, C., Coleman, R.J., Garrett, C., Gloyn, A.L., Edghill, E.L., Hattersley, A.T., Wellauer, P.K., et al. (2004). Mutations in PTF1A cause pancreatic and cerebellar agenesis. Nat. Genet. *36*, 1301–1305.

Shaw-Smith, C., De Franco, E., Lango Allen, H., Batlle, M., Flanagan, S.E., Borowiec, M., Taplin, C.E., van Alfen-van der Velden, J., Cruz-Rojo, J., Perez de Nanclares, G., et al. (2014). GATA4 mutations are a cause of neonatal and childhood-onset diabetes. Diabetes *63*, 2888–2894.

Shi, Z.D., Lee, K., Yang, D., Amin, S., Verma, N., Li, Q.V., Zhu, Z., Soh, C.L., Kumar, R., Evans, T., et al. (2017). Genome editing in hPSCs reveals GATA6 haploinsufficiency and a genetic interaction with GATA4 in human pancreatic development. Cell Stem Cell *20*, 675–688.e6.

Smith, S.B., Qu, H.Q., Taleb, N., Kishimoto, N.Y., Scheel, D.W., Lu, Y., Patch, A.M., Grabs, R., Wang, J., Lynn, F.C., et al. (2010). Rfx6 directs islet formation and insulin production in mice and humans. Nature *463*, 775–780.

Stanescu, D.E., Hughes, N., Patel, P., and De Leon, D.D. (2015). A novel mutation in GATA6 causes pancreatic agenesis. Pediatr. Diabetes *16*, 67–70.

Stoffers, D.A., Zinkin, N.T., Stanojevic, V., Clarke, W.L., and Habener, J.F. (1997). Pancreatic agenesis attributable to a single nucleotide deletion in the human IPF1 gene coding sequence. Nat. Genet. *15*, 106–110.

Suzuki, S., Nakao, A., Sarhat, A.R., Furuya, A., Matsuo, K., Tanahashi, Y., Kajino, H., and Azuma, H. (2014). A case of pancreatic agenesis and congenital heart defects with a novel GATA6 nonsense mutation: evidence of haploinsufficiency due to nonsense-mediated mRNA decay. Am. J. Med. Genet. A *164A*, 476–479.

Teo, A.K., Arnold, S.J., Trotter, M.W., Brown, S., Ang, L.T., Chng, Z., Robertson, E.J., Dunn, N.R., and Vallier, L. (2011). Pluripotency factors regulate definitive endoderm specification through eomesodermin. Genes Dev. *25*, 238–250.

Teo, A.K., Tsuneyoshi, N., Hoon, S., Tan, E.K., Stanton, L.W., Wright, C.V., and Dunn, N.R. (2015). PDX1 binds and represses hepatic genes to ensure robust pancreatic commitment in differentiating human embryonic stem cells. Stem Cell Reports *4*, 578–590.

Tiyaboonchai, A., Cardenas-Diaz, F.L., Ying, L., Maguire, J.A., Sim, X., Jobaliya, C., Gagne, A.L., Kishore, S., Stanescu, D.E., Hughes, N., et al. (2017). GATA6 plays an important role in the induction of human definitive endoderm, development of the pancreas, and functionality of pancreatic beta cells. Stem Cell Reports *8*, 589–604.

Vallier, L., Touboul, T., Chng, Z., Brimpari, M., Hannan, N., Millan, E., Smithers, L.E., Trotter, M., Rugg-Gunn, P., Weber, A., et al. (2009). Early cell fate decisions of human embryonic stem cells and mouse epiblast stem cells are controlled by the same signalling pathways. PLoS One *4*, e6082.

Viger, R.S., Guittot, S.M., Anttonen, M., Wilson, D.B., and Heikinheimo, M. (2008). Role of the GATA family of transcription factors in endocrine development, function, and disease. Mol. Endocrinol. *22*, 781–798.

Wang, S., Sun, H., Ma, J., Zang, C., Wang, C., Wang, J., Tang, Q., Meyer, C.A., Zhang, Y., and Liu, X.S. (2013). Target analysis by integration of transcriptome and ChIP-seq data with BETA. Nat. Protoc. *8*, 2502–2515.

Xuan, S., Borok, M.J., Decker, K.J., Battle, M.A., Duncan, S.A., Hale, M.A., Macdonald, R.J., and Sussel, L. (2012). Pancreas-specific deletion of mouse Gata4 and Gata6 causes pancreatic agenesis. J. Clin. Invest. *122*, 3516–3528.

Yau, D., De Franco, E., Flanagan, S.E., Ellard, S., Blumenkrantz, M., and Mitchell, J.J. (2017). Case report: maternal mosaicism resulting in inheritance of a novel GATA6 mutation causing pancreatic agenesis and neonatal diabetes mellitus. Diagn. Pathol. *12*, 1.

Yorifuji, T., Kawakita, R., Hosokawa, Y., Fujimaru, R., Yamaguchi, E., and Tamagawa, N. (2012). Dominantly inherited diabetes mellitus caused by GATA6 haploinsufficiency: variable intrafamilial presentation. J. Med. Genet. *49*, 642–643.

Yu, L., Bennett, J.T., Wynn, J., Carvill, G.L., Cheung, Y.H., Shen, Y., Mychaliska, G.B., Azarow, K.S., Crombleholme, T.M., Chung, D.H., et al. (2014). Whole exome sequencing identifies de novo mutations in GATA6 associated with congenital diaphragmatic hernia. J. Med. Genet. *51*, 197–202.

Zhao, R., Watt, A.J., Li, J., Luebke-Wheeler, J., Morrisey, E.E., and Duncan, S.A. (2005). GATA6 is essential for embryonic development of the liver but dispensable for early heart formation. Mol. Cell. Biol. *25*, 2622–2631.

Stem Cell Reports, Volume *12*

Supplemental Information

GATA6 Cooperates with EOMES/SMAD2/3 to Deploy the Gene Regulatory Network Governing Human Definitive Endoderm and Pancreas Formation

Crystal Y. Chia, Pedro Madrigal, Simon L.I.J. Denil, Iker Martinez, Jose Garcia-Bernardo, Ranna El-Khairi, Mariya Chhatriwala, Maggie H. Shepherd, Andrew T. Hattersley, N. Ray Dunn, and Ludovic Vallier

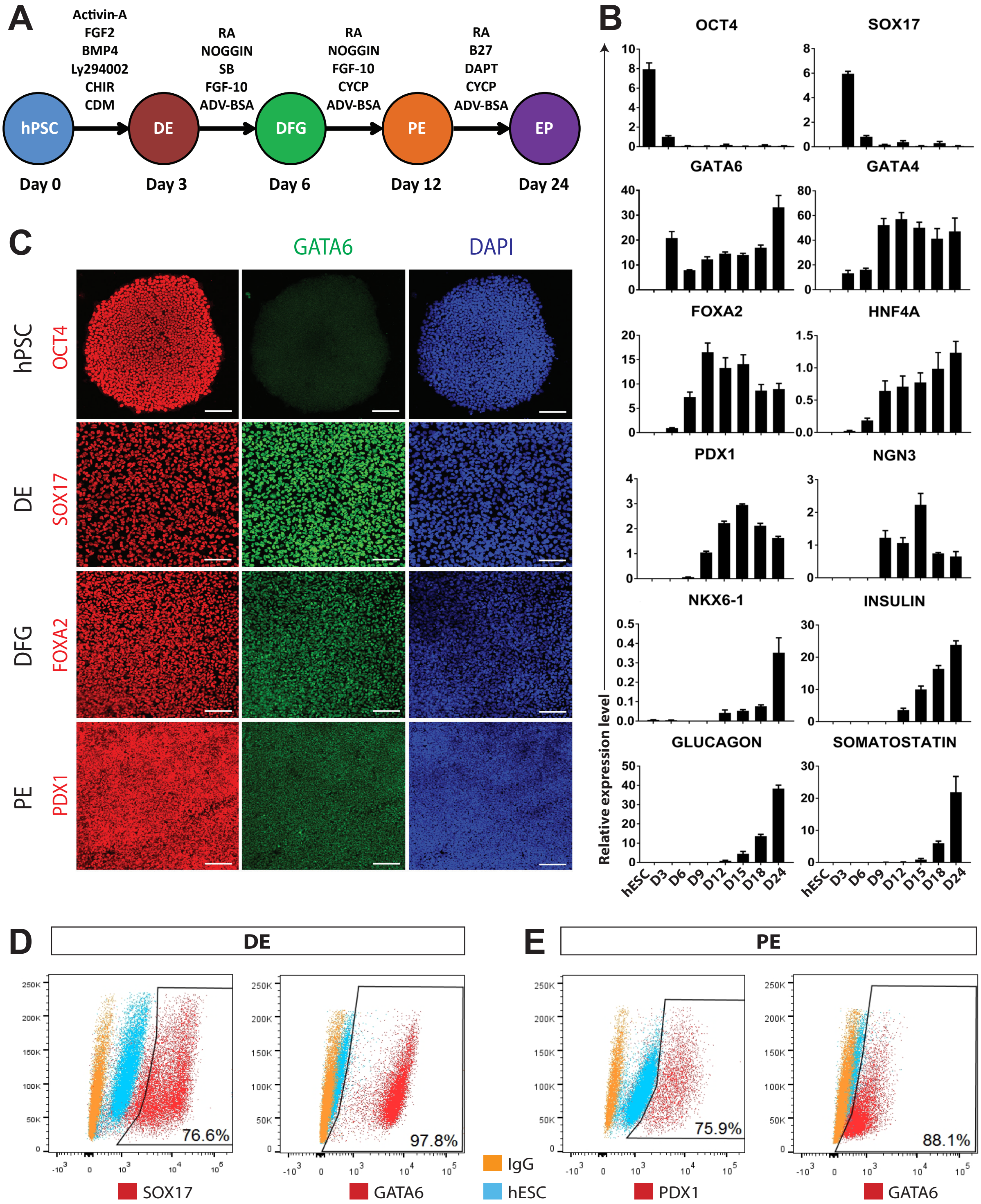
A
Activin-A
FGF2
BMP4
Ly294002
CHIR
CDM
RA
NOGGIN
SB
FGF-10
ADV-BSA
RA
NOGGIN
FGF-10
CYCP
ADV-BSA
RA
B27
DAPT
CYCP
ADV-BSA
hPSC
DE
DFG
PE
EP
Day 0
Day 3
Day 6
Day 12
Day 24
B
OCT4
SOX17
GATA6
GATA4
FOXA2
HNF4A
PDX1
NGN3
NKX6-1
INSULIN
GLUCAGON
SOMATOSTATIN
Relative expression level
hESC D3 D6 D9 D12 D15 D18 D24
C
GATA6
DAPI
hPSC OCT4
DE SOX17
DFG FOXA2
PE PDX1
D
DE
76.6%
97.8%
SOX17
GATA6
IgG
hESC
E
PE
75.9%
88.1%
PDX1
GATA6

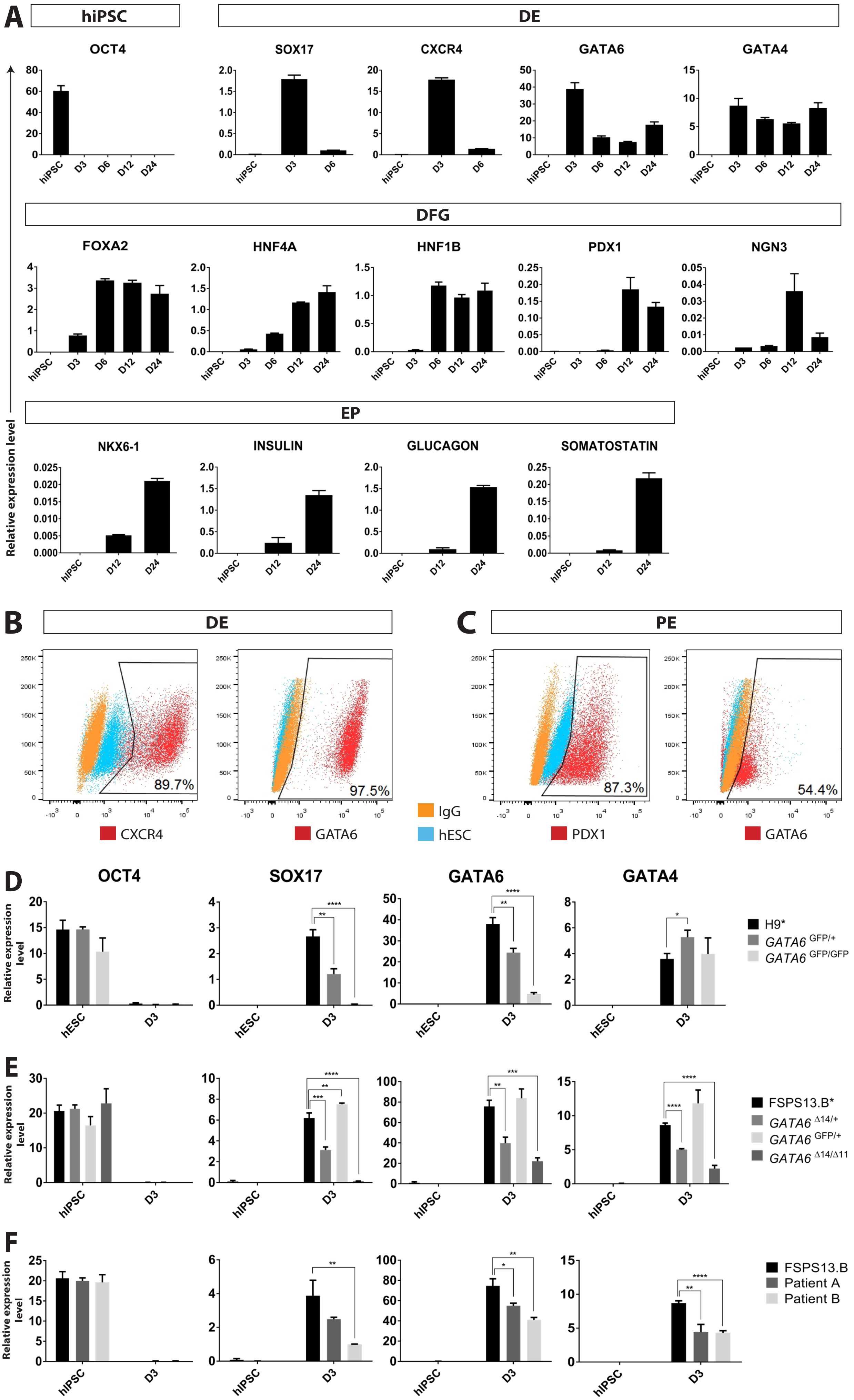

A
hiPSC
DE
OCT4
SOX17
CXCR4
GATA6
GATA4
DFG
FOXA2
HNF4A
HNF1B
PDX1
NGN3
EP
NKX6-1
INSULIN
GLUCAGON
SOMATOSTATIN
Relative expression level
hiPSC
D3
D6
D12
D24
B
DE
89.7%
97.5%
CXCR4
GATA6
IgG
hESC
C
PE
87.3%
54.4%
PDX1
GATA6
D
OCT4
SOX17
GATA6
GATA4
Relative expression level
hESC
D3
H9*
GATA6 GFP/+
GATA6 GFP/GFP
E
Relative expression level
hIPSC
D3
FSPS13.B*
GATA6 Δ14/+
GATA6 GFP/+
GATA6 Δ14/Δ11
F
Relative expression level
hIPSC
D3
FSPS13.B
Patient A
Patient B

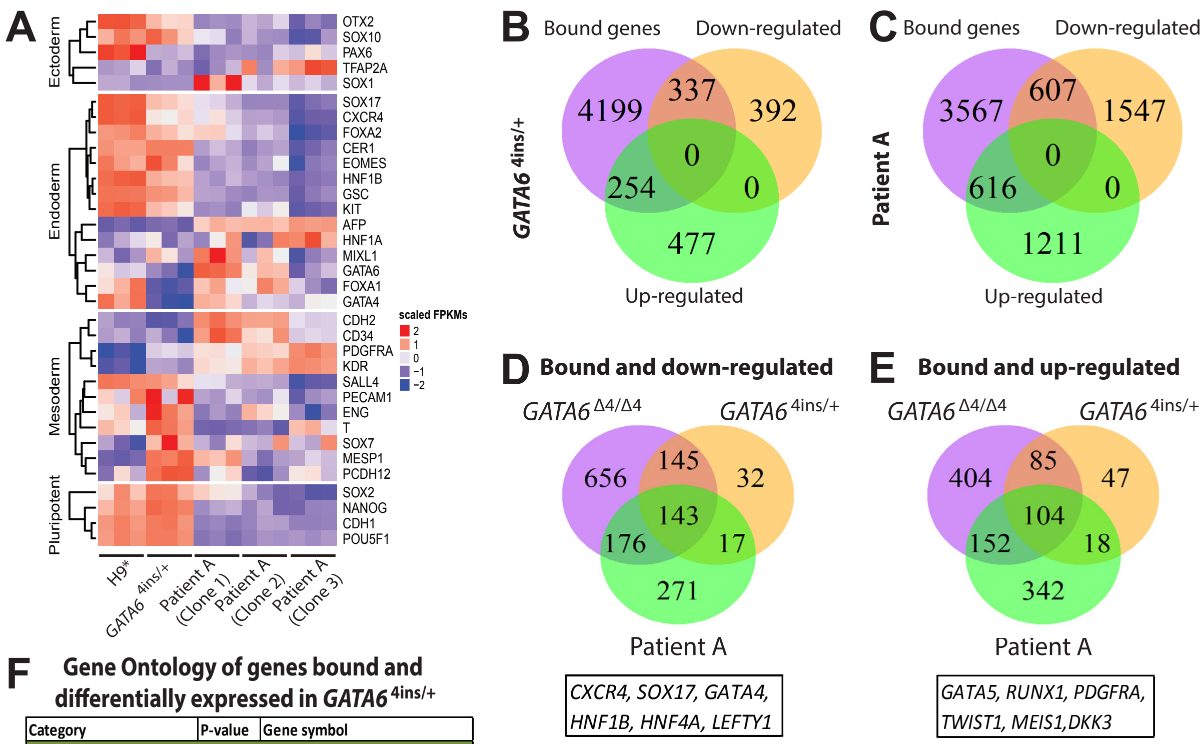

F Gene Ontology of genes bound and differentially expressed in *GATA6* $^{4ins/+}$

Category	P-value	Gene symbol
**Up-regulated in WT**		
Endoderm development	6.78E-04	GDF3, COL4A2, NOG, HNF1B, NODAL, SMAD2, MMP15, HMGA2, HSBP1, DUSP5, HHEX, DUSP1, GATA6, ITGA7, COL11A1
**Up-regulated in *GATA6* $^{4ins/+}$**		
Mesoderm formation	3.31E-04	FGFR2, SIX2, ITGA3, WLS, SMAD1, ITGB1, SNAI1, WNT3, DKK1, HAND1, SFRP2, ITGA8, FOXC1, TLX2

G Gene Ontology of genes bound and differentially expressed in Patient A

Category	P-value	Gene symbol
**Up-regulated in WT**		
Endoderm development	0.02678	GDF3, NANOG, HNF1B, ONECUT1, NODAL, EOMES, MMP15, KIF16B, HSBP1, ZFP36L1, HHEX, LHX1, ITGA7, COL6A1
**Up-regulated in Patient A**		
Mesoderm development	0.00119	FGFR2,PAX2, WNT3, OSR1, HAND1, YAP1, TBX3, SMAD4, SIX2, SMAD3, ITGA2, ITGA3, SMAD1, DKK1, ITGA8

H

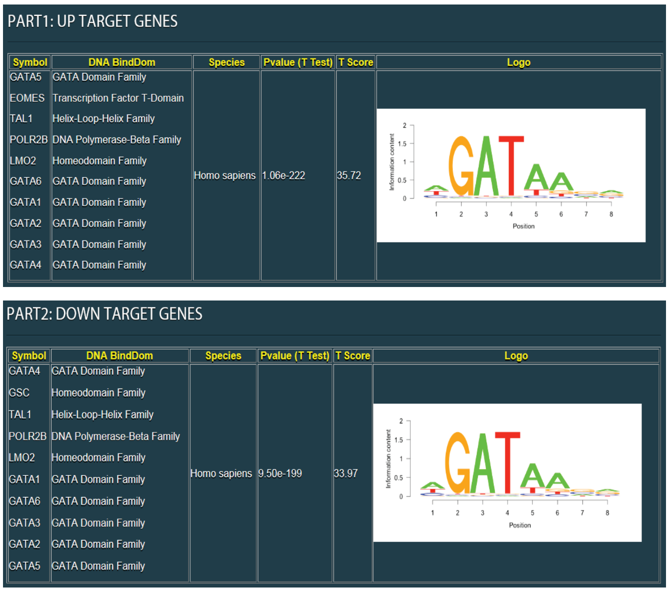
PART1: UP TARGET GENES

Symbol	DNA BindDom	Species	Pvalue (T Test)	T Score	Logo
GATA5	GATA Domain Family	Homo sapiens	1.06e-222	35.72	
EOMES	Transcription Factor T-Domain				
TAL1	Helix-Loop-Helix Family				
POLR2B	DNA Polymerase-Beta Family				
LMO2	Homeodomain Family				
GATA6	GATA Domain Family				
GATA1	GATA Domain Family				
GATA2	GATA Domain Family				
GATA3	GATA Domain Family				
GATA4	GATA Domain Family				

PART2: DOWN TARGET GENES

Symbol	DNA BindDom	Species	Pvalue (T Test)	T Score	Logo
GATA4	GATA Domain Family	Homo sapiens	9.50e-199	33.97	
GSC	Homeodomain Family				
TAL1	Helix-Loop-Helix Family				
POLR2B	DNA Polymerase-Beta Family				
LMO2	Homeodomain Family				
GATA1	GATA Domain Family				
GATA6	GATA Domain Family				
GATA3	GATA Domain Family				
GATA2	GATA Domain Family				
GATA5	GATA Domain Family				

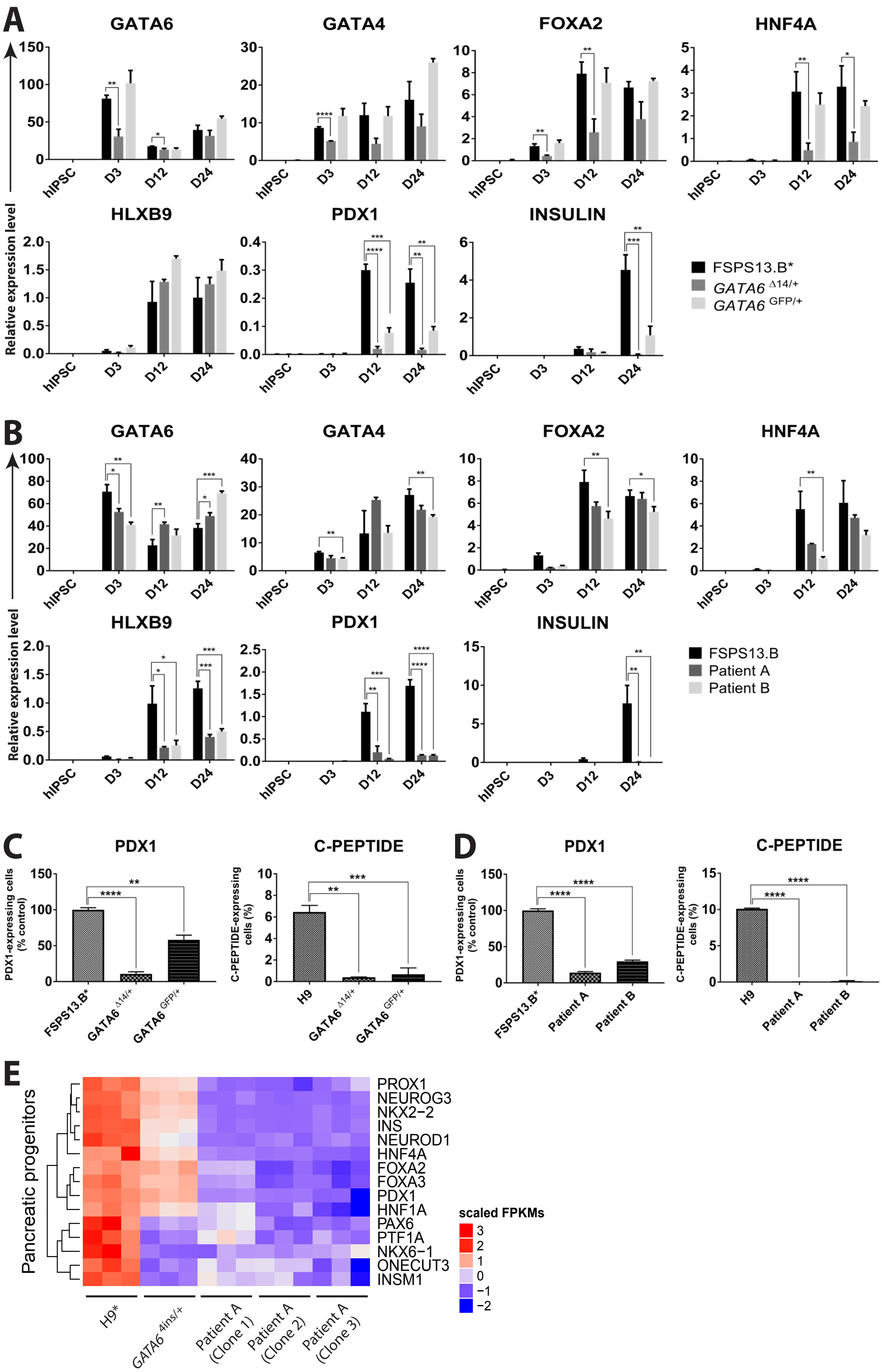
A
GATA6
GATA4
FOXA2
HNF4A
HLXB9
PDX1
INSULIN
Relative expression level
hIPSC
D3
D12
D24
FSPS13.B*
GATA6 Δ14/+
GATA6 GFP/+
B
GATA6
GATA4
FOXA2
HNF4A
HLXB9
PDX1
INSULIN
Relative expression level
FSPS13.B
Patient A
Patient B
C
PDX1
C-PEPTIDE
PDX1-expressing cells (% control)
C-PEPTIDE-expressing cells (%)
FSPS13.B*
GATA6 Δ14/+
GATA6 GFP/+
H9
D
PDX1
C-PEPTIDE
PDX1-expressing cells (% control)
C-PEPTIDE-expressing cells (%)
FSPS13.B*
Patient A
Patient B
H9
E
Pancreatic progenitors
PROX1
NEUROG3
NKX2−2
INS
NEUROD1
HNF4A
FOXA2
FOXA3
PDX1
HNF1A
PAX6
PTF1A
NKX6−1
ONECUT3
INSM1
scaled FPKMs
H9*
GATA6 4ins/+
Patient A (Clone 1)
Patient A (Clone 2)
Patient A (Clone 3)

SUPPLEMENTAL FIGURE LEGENDS

Supplemental Figure 1. Directed differentiation of H9 cells into the pancreatic lineage.

(A) Schematic of the 24-day differentiation protocol. DE, definitive endoderm; DFG, dorsal foregut; PE, pancreatic endoderm; EP, endocrine progenitors. The culture medium and supplements indicated are BMP (Bone Morphogenetic Protein 4), the PI3 kinase inhibitor Ly294002, CHIR (the GSK3 inhibitor CHIR99021), CDM (Chemically Defined Medium), Adv-BSA (Advanced Dulbecco's Modified Eagle Medium/Ham's F-12 medium supplemented with BSA and L-glutamine), RA (retinoic acid), SB (the ALK4/5/78 inhibitor SB-431542), FGF2 (Fibroblast Growth Factor 2), FGF10 (Fibroblast Growth Factor 10), CYCP (the Hedgehog inhibitor Cyclopamine-KAAD), B27 supplement, and the NOTCH inhibitor DAPT.

(B) Expression of key marker genes during pancreatic differentiation in H9 cells. n = 3 independent experiments for each stage of differentiation.

(C) Immunofluorescence analyses showing co-expression of GATA6 with SOX17, FOXA2, and PDX1. DAPI, 4', 6-diamidino-2-phenylindole dihydrochloride. Scale bars, 100 µm.

(D, E) Differentiation efficiency measured by FACS analysis of SOX17 and GATA6 at day 3 definitive endoderm, and PDX1 and GATA6 at day 12 pancreatic endoderm. Undifferentiated hESC stained with the respective primary and secondary antibodies and secondary antibody only (IgG) were both used as controls. Gates were set according to hESC control.

Supplemental Figure 2. Directed differentiation of FSPS13.B cells into the pancreatic lineage, and mutant hPSC lines and Patients A and B display impaired DE formation.

(A) Expression of key marker genes during pancreatic differentiation in FSPS13.B cells. n = 3 independent experiments for each stage of differentiation (**Supp. Fig. 1A**).

(B, C) Differentiation efficiency measured by FACS analysis of CXCR4 and GATA6 at day 3 definitive endoderm, and PDX1 and GATA6 at day 12 pancreatic endoderm. Undifferentiated hESC stained with the respective primary and secondary antibodies and secondary antibody only (IgG) were both used as controls. Gates were set according to hESC control.

(D) Expression of pluripotency (*OCT4*) and definitive endoderm (*SOX17, GATA6* and *GATA4*) markers at day 3 DE in H9* and H9-derived $GATA6^{GFP/+}$ and $GATA6^{GFP/GFP}$ mutant cells.

(E) Expression of pluripotency (*OCT4*) and definitive endoderm (*SOX17, GATA6* and *GATA4*) markers at day 3 DE in FSBS13.B* and FSBS13.B-derived *GATA6*$^{\Delta14/+}$, *GATA6*$^{GFP/+}$ and *GATA6*$^{\Delta14/\Delta11}$ mutant cells.

(F) Expression of pluripotency (*OCT4*) and definitive endoderm (*SOX17, GATA6* and *GATA4*) markers at day 3 DE in Patient A and Patient B mutant cells.

(D-F) Error bars represent the SE of three independent experiments. $^{*}p < 0.05$, $^{**}p < 0.01$, $^{***}p < 0.001$, $^{****}p < 0.0001$.

Supplemental Figure 3. *GATA6* is a key regulator of the DE transcriptional network.

(A) Heat map illustrating differential gene expression of key germ layer markers via RNA-seq between H9* cells, H9-derived *GATA6*$^{4ins/+}$ and clones 1, 2 and 3 of Patient A mutant cells at the DE stage. *n* = 3 biological replicates for each cell line.

(B, C) Venn diagram indicating the overlap of *GATA6*-bound genes from ChIP-seq at the DE stage with downregulated or upregulated genes of H9-derived *GATA6*$^{4ins/+}$ mutant cells or Patient A compared to H9* cells derived from RNA-seq.

(D, E) Venn diagram indicating the triple overlap of *GATA6*-bound genes from ChIP-seq at the DE stage with downregulated or upregulated genes of H9-derived *GATA6*$^{\Delta4/\Delta4}$ mutant cells compared to H9* cells derived from RNA-seq. Key bound genes up- or downregulated are indicated in the respective tables.

(F, G) Enriched gene ontology showing developmental pathways from direct target genes differentially expressed between H9* and H9-derived *GATA6*$^{4ins/+}$ or H9* and Patient A mutant cells derived from BETA analysis.

(H) Motif analysis of up and down target genes derived from BETA analysis.

Supplemental Figure 4. Decreased levels of *GATA6* impact downstream pancreatic differentiation.

(A) Expression of DE (*GATA6, GATA4* and *FOXA2*), pancreatic (*HNF4A, HLXB9* and *PDX1*), and endocrine (*INSULIN*) marker genes in FSPS13.B-derived *GATA6*$^{\Delta14/+}$, *GATA6*$^{GFP/+}$ mutant cells at key stages of the 24-day pancreatic differentiation protocol (**Supp. Fig. 1A**).

(B) Expression of DE (*GATA6, GATA4* and *FOXA2*), pancreatic (*HNF4A, HLXB9* and *PDX1*), and endocrine (*INSULIN*) marker genes in Patient A and Patient B mutant cells.

(C) Percentage PDX1-positive cells in FSPS13.B-derived *GATA6* $^{\Delta 14/+}$ and *GATA6*$^{GFP/+}$ lines at day 12 shown relative to FSPS13.B* (100%) as measured by FACS. Absolute percentage of C-PEPTIDE-positive cells in FSPS13.B* and FSPS13.B-derived *GATA6*$^{\Delta 14/+}$ and *GATA6*$^{GFP/+}$ lines at the EP stage (day 24).

(D) Percentage PDX1-positive cells in Patient A and B lines at day 12 shown relative to FSPS13.B (100%) as measured by FACS. Absolute percentage of C-PEPTIDE-positive cells in Patient A and B lines at the EP stage (day 24).

(E) Heat map illustrating differential gene expression of key pancreatic progenitor markers via RNA-seq between H9*cells, H9-derived *GATA6*$^{4ins/+}$ and clones 1, 2 and 3 of Patient A at the PE stage. n = 3 biological replicates for each cell line.

(A-D) Error bars represent the SE of three independent experiments. $^{*}p < 0.05$, $^{**}p < 0.01$, $^{***}p < 0.001$, $^{****}p < 0.0001$.

Supplemental Table 2

**TALEN target sites for *GATA6***		
**TALEN pair**	**Name**	**Sequence (5' → 3')**
After first ATG	Left arm	GACTGACGGCGGCTGGT
	Right arm	CCGCACCCGCGGCCCCG
After second ATG	Left arm	GCTGCCCGGCCTACCGT
	Right arm	GGCTGGCCCACTGCCC
**Primers used to screen for mutations**		
**TALEN target site**		**Primer sequence (5' → 3')**
After first ATG	F	CTTTGAGAAGTCAGATCCCATTTGA
	R	CGCCTCCGCTGCCGTATGGAGGGCT
After second ATG	F	CGCCAGCAAGCTGCTGTGGTCCAGC
	R	TCCGCGCACCCGGACGAGAAAGTCC

**Primers used to assemble TALEN repeat arrays**	
**Primer name**	**Primer sequence (5' → 3')**
TALEN-RVDs 1 Fwd	CTGACCCCAGACCAGGTAGTCGCA
TALEN-RVDs 1 Rev	CACGACTTGATCCGGTGTAAGGCCGTGGTCTTGACAAAGG
TALEN-RVDs 2 Fwd	CCTTTGTCAAGACCACGGCCTTACACCGGATCAAGTCGTG
TALEN-RVDs 2 Rev	TACAACTTGATCGGGAGTCAGCCCGTGgtCTTGACAGAGA
TALEN-RVDs 3 Fwd	TCTCTGTCAAGacCACGGGCTGACTCCCGATCAAGTTGTA
TALEN-RVDs 3 Rev	GACCACTTGgtCAGGCGTCAAACCGTGatCTTGACACAAC
TALEN-RVDs 4 Fwd	GTTGTGTCAAGatCACGGTTTGACGCCTGacCAAGTGGTC
TALEN-RVDs 4 Rev	TCCATGATCCTGGCACAGTACAGG
TALEN-RVDs 1-4 Fwd	tcagGGTCTCAGAACCTGACCCCAGACCAGGTAGTC
TALEN-RVDs 1-4 Rev	tcagGGTCTCTAGTCCATGATCCTGGCACAGT
TALEN-RVDs 5-8 Fwd	tcagGGTCTCAGACTGACCCCAGACCAGGTAGTC

TALEN-RVDs 5-8 Rev	tcagGGTCTCTGTCAGTCCATGATCCTGGCACAGT
TALEN-RVDs 9-12 Fwd	tcagGGTCTCATGACCCCAGACCAGGTAGTC
TALEN-RVDs 9-12 Rev	tcagGGTCTCTCAGTCCATGATCCTGGCACAGT
TALEN-RVDs 13-16 Fwd	tcagGGTCTCAACTGACCCCAGACCAGGTAGTC
TALEN-RVDs 13-16 Rev	tcagGGTCTCTTCAGTCCATGATCCTGGCACAGT
**Primers used to construct the donor plasmid**	
**Primer name**	**Primer sequence (5' → 3')**
5' Arm-KpnI-GATA6 Fwd	tcagGGTACCTTTGGGGTCGCCTCGGCTCTGG
5' Arm-GATA6 Rev	CTTGCTCACCATGGTGGCCACGGTCCGGCGCCGCTCCAA
5' Arm-GATA6-emGFP Fwd	CGCCGGACCGTGGCCACCATGGTGAGCAAGGGCGAGGAGC
3' Arm-XbaI-TALEN1 Fwd	tcagTCTAGAAAGCGCTTCGGGGCCGCGGGTG
3' Arm-SacI-TALEN1 Rev	tcagGAGCTCTGGCGCCCCCACGTAGGGCGAG
**Primers used for sequencing donor plasmid**	
**Primer name**	**Primer sequence (5' → 3')**
EmGFP3'-Fwd	TCACATGGTCCTGCTGGAGTTC
BGHpA-mid-Rev	TTAGGAAAGGACAGTGGGAGTG
EmGFP5'-Rev	CGCTGAACTTGTGGCCGTTTAC
EmGFP-mid-Rev	GACCTTGTGGCTGTTGTAGTTG
mPGKpA-Fwd	AAGAAGGGTGAGAACAGAGTACC
M13-Rev (-24)	GGAAACAGCTATGACCATG
M13-Fwd (-20)	GTAAAACGACGGCCAGT
pCAGGS pre-SA Fwd	CTGCTAACCATGTTCATGCCTTC

Supplemental Table 2: Primers supporting Figure 1.

Sequence of left and right TALEN arms for *GATA6* mutant generation at two different cut sites in exon 2, sequence of primers used to screen for mutations, assemble the TALEN repeat arrays, construct and sequence the donor plasmid.

Supplemental Table 3

**Gene**		**Primer sequence (5’ → 3’)**
OCT4	F	AGTGAGAGGCAACCTGGAGA
	R	ACACTCGGACCACATCCTTC
SOX2	F	TGGACAGTTACGCGCACAT
	R	CGAGTAGGACATGCTGTAGGT
BRACHURY	F	TGCTTCCCTGAGACCCAGTT
	R	GATCACTTCTTTCCTTTGCATCAAG
EOMESODERMIN	F	ATCATTACGAAACAGGGCAGGC
	R	CGGGGTTGGTATTTGTGTAAGG
GATA4	F	TCCCTCTTCCCTCCTCAAAT
	R	TCAGCGTGTAAAGGCATCTG
GATA6	F	TGTGCAATGCTTGTGGACTC
	R	AGTTGGAGTCATGGGAATGG
SOX17	F	CGCACGGAATTTGAACAGTA
	R	GGATCAGGGACCTGTCACAC
CXCR4	F	CACCGCATCTGGAGAACCA
	R	GCCCATTTCCTCGGTGTAGTT
FOXA2	F	GGGAGCGGTGAAGATGGA
	R	TCATGTTGCTCACGGAGGAGTA
GCG	F	AAGCATTTACTTTGTGGCTGGATT
	R	TGATCTGGATTTCTCCTCTGTGTCT
HLXB9	F	CACCGCGGGCATGATC
	R	ACTTCCCCAGGAGGTTCGA
HNF4A	F	CATGGCCAAGATTGACAACCT
	R	TTCCCATATGTTCCTGCATCAG
INSULIN	F	GAAGCGTGGCATTGTGGAAC
	R	GCTGCGTCTAGTTGCAGTAGT
NGN3	F	GCTCATCGCTCTCTATTCTTTTGC
	R	GGTTGAGGCGTCATCCTTTCT
NKX6.1	F	GGCCTGTACCCCTCATCAAG
	R	TCCGGAAAAAGTGGGTCTCG
PDX1	F	GATTGGCGTTGTTTGTGGCT
	R	GCCGGCTTCTCTAAACAGGT
SST	F	CCCCAGACTCCGTCAGTTTC
	R	TCCGTCTGGTTGGGTTCAG
PBGD	F	GGAGCCATGTCTGGTAACGG
	R	CCACGCGAATCACTCTCATCT

Supplemental Table 3: Table of forward and reverse primers used for RT-qPCR supporting Fig. 2 and 4, and Supp. Fig. 1, 2, and 4.

Supplemental Table 4

Primary antibody for Immunofluorescence (IF) staining	Dilution ratio	Duration
Goat anti-human Nanog (R&D, #AF1997)	1:100	Overnight
Goat anti-human Sox2 (R&D, #AF2018)	1:100	Overnight
Goat anti-human Oct4 (Santa Cruz, #sc-8628)	1:100	Overnight
Rabbit anti-human GATA6 (Cell Signaling, #5851)	1:200	Overnight
Goat anti-human Sox17 (R&D, #AF1924)	1:200	Overnight
Goat anti-human FoxA2 (R&D, #AF2400)	1:100	Overnight
Goat anti-human PDX1 (R&D, #AF2419)	1:100	Overnight
Mouse anti-human C-Peptide (Acris Antibodies, #BM270S)	1:100	Overnight
Goat anti-human Glucagon G-17 (Santa Cruz, #sc7780)	1:100	Overnight
Rabbit anti-human Somatostatin (Daka, #A0566)	1:200	Overnight
**Secondary antibody for Immunofluorescence (IF) staining**	**Dilution ratio**	**Duration**
Alexa Fluor 568 Donkey Anti-Goat IgG (H+L) (Invitrogen, #A11057)	1:1000	1 hr
Alexa Fluor 568 Donkey Anti-Mouse IgG (H+L) (Invitrogen, #A10037)	1:1000	1 hr
Alexa Fluor 568 Donkey Anti-Rabbit IgG (H+L) (Invitrogen, #A10042)	1:1000	1 hr
Alexa Fluor 488 Donkey anti-Goat IgG (H+L) (Invitrogen, #A11055)	1:1000	1 hr
Alexa Fluor 488 Donkey anti-Mouse IgG (H+L) (Invitrogen, #A21202)	1:1000	1 hr
Alexa Fluor 488 Donkey anti-Rabbit IgG (H+L) (Invitrogen, #A21206)	1:1000	1 hr
Alexa Fluor 647 Donkey anti-Goat IgG (H+L) (Invitrogen, #A21447)	1:1000	1 hr
Alexa Fluor 647 Donkey anti-Mouse IgG (H+L) (Invitrogen, #A31571)	1:1000	1 hr
Alexa Fluor 647 Donkey anti-Rabbit IgG (H+L) (Invitrogen, #A31573)	1:1000	1 hr
**Primary antibody for FACS analysis**	**Dilution ratio**	**Duration**
Goat anti-human Sox17 (R&D, #AF1924)	1:20	2 hr
Rabbit anti-human GATA6 (Cell Signaling, #5851)	1:20	2 hr
Goat anti-human PDX1 (R&D, #AF2419)	1:20	2 hr
Mouse anti-human C-Peptide (Acris Antibodies, #BM270S)	1:100	2 hr

Goat anti-human Glucagon G-17 (Santa Cruz, #sc7780)	1:20	2 hr
Rabbit anti-human Somatostatin (Daka, #A0566)	1:200	2 hr
**Secondary antibody for FACS analysis**	**Dilution ratio**	**Duration**
Alexa Fluor 568 Donkey Anti-Goat IgG (H+L) (Invitrogen, #A11057)	1:1000	30 min
Alexa Fluor 568 Donkey Anti-Mouse IgG (H+L) (Invitrogen, #A10037)	1:1000	30 min
Alexa Fluor 568 Donkey Anti-Rabbit IgG (H+L) (Invitrogen, #A10042)	1:1000	30 min
Alexa Fluor 647 Donkey anti-Mouse IgG (H+L) (Invitrogen, #A31571)	1:1000	30 min
Alexa Fluor 488 Donkey anti-Rabbit IgG (H+L) (Invitrogen, #A21206)	1:1000	30 min
**Conjugated primary and secondary antibody for FACS analysis**	**Dilution ratio**	**Duration**
Anti-Human CD184 (CXCR4) PE (eBioscience, #12-9999-41)	1:50	1 hr
**Primary antibody for western blotting**	**Dilution ratio**	**Duration**
Rabbit anti-human GATA6 (N-terminus; Cell Signaling, #5851)	1:2000	2 hr
Rabbit anti-human GATA6 (C-terminus; Cell Signalling, #4253)	1:2000	2 hr
Rabbit anti-human GATA4 (Cell Signalling, #36966)	1:2000	2 hr
Mouse anti-alpha-Tubulin (Sigma-Aldrich, #T6199)	1:5000	1 hr
**Secondary antibody for western blotting**	**Dilution ratio**	**Duration**
Anti-Rabbit IgG- Peroxidase antibody produced in goat (Sigma-Aldrich, #A6154)	1:10000	1 hr
Anti-Mouse IgG- Peroxidase antibody produced in goat (Sigma-Aldrich, #A5278)	1:10000	1 hr

Supplemental Table 4: Primary, conjugated and secondary antibodies supporting Fig. 1, 2 and 4, and Supp. Fig. 1, 2 and 4.

Tables of primary and secondary antibodies used for Immunofluorescence, FACS and western blotting.

SUPPLEMENTAL EXPERIMENTAL PROCEDURES

Modifications in pancreatic differentiation

(1) 3 µM CHIR99201 was added on day 1, (2) BMP4 and LY294002 were excluded on day 3, (3) 2 µM retinoic acid (RA) and 0.25 µg/ml KAAD-cyclopamine were included on days 10-12 and 16-18, (4) 2 µM RA and 0.1 mM 6-Bnz-cAMP sodium salt (BNZ; Sigma-Aldrich, #B4560) were included on days 13-15, and (5) the protocol was extended from 18 to 24 days where 1% B27, 2 µM RA acid and 0.25 µg/ml KAAD-cyclopamine were added on days 19-24.

Assembly of the TALEN vectors and donor vector

The TALEN vectors were assembled using the Joung Lab REAL Assembly TALEN kit (Addgene, #1000000017) (Sander et al., 2007). The pTAL scaffold was modified to a second generation GoldyTALEN scaffold, which was shown to improve genome editing efficiency (Bedell et al., 2012). In addition, NN repeat variable domains (RVDs) were modified to become NH. Suitable TALEN target sites in the *GATA6* gene were first generated using an online TALEN targeter software tool (Cermak et al., 2011; Doyle et al., 2012). TALEN targets were selected based on higher numbers of HDs (= C) and NHs (= G) for stronger binding (Streubel et al., 2012) and the presence of a restriction enzyme site in the spacer region to aid in screening. For vector construction, the selected target sequences were entered into a ZiFiT targeter software (Sander et al., 2010; Sander et al., 2007). The sequences of the first and second selected TALEN target pairs are 5' GACTGACGGCGGCTGGT 3' (left) and 5' CCGCACCCGCGGCCCCG 3' (right), and 5' GCTGCCCGGCCTACCGT 3' (left) and 5' GGCTGGCCCACTGCCC 3' (right), respectively. The TALEN vectors were then assembled using a three-step PCR approach to combine the RVDs. The success of the TALEN assembly was verified by Sanger sequencing.

Next, the assembled TALEN RVDs were cloned into vectors containing a CAG promoter and a puromycin, zeocin or blasticidin antibiotic resistant gene. Vectors used to generate mutants via the NHEJ pathway contained the puromycin and zeocin antibiotic resistant gene for the left and right TALEN arms, respectively. Vectors used to generate mutants via the HR pathway contained the blasticidin and zeocin antibiotic resistant gene for the left and right TALEN arms, respectively. The final TALEN constructs were then sequenced to confirm that the TALEN arms were cloned in the correct orientation using the following forward and reverse primers 5' AATACGACTCACTATAG 3' and 5' AACTTTTAAACCGGTCTCGAGCTGA 3' respectively.

A donor vector aimed at terminating transcription of *GATA6* prematurely by inserting a 'donor template' through HR was also constructed. Within the donor vector is a cassette which contains 5' and 3' homology arms each 1kb in length recognising the flanking regions of the TALEN 1 target

site, an EmGFP gene, a puromycin antibiotic resistant cassette and a polyA tail. Primers used to construct the donor vector are listed in **Supp. Table 2**. The final construct was sequenced to confirm that the donor vector was cloned successfully. Primers used to sequence the donor vector are listed in **Supp. Table 2**.

Electroporation and screening of drug-resistant clones

TALEN vectors were introduced into cells via electroporation (Human Stem Cell Nucleofector Kit 1, Lonza) using the Amaxa Nucleofector. Briefly, cells were harvested after treatment with StemPro Accutase Cell Dissociation Reagent (Gibco, #A1110501) and counted. 8 x 10^5 cells were used for each electroporation. Electroporation was performed according to the manufacturer's recommendations and cells were plated with ROCK inhibitor Y-27632 (Sigma-Aldrich, #Y0503). 24-hour antibiotic selection using puromycin (1 µg/ml; Sigma-Aldrich, #P8833), zeocin (2.5 µg/ml; Gibco, #R250-01) or blasticidin (3.5 µg/ml; Sigma-Aldrich, #15205) was started 24 hours after electroporation. Individual colony screening was carried out by PCR on genomic DNA with primers listed in **Supp. Table 2**. PCR products were sub-cloned when necessary to determine the precise mutation(s).

Multiplex fluorescence *in situ* hydridization (M-FISH) karyotyping

For each cell line, 10-20 randomly selected metaphases were karyotyped based on multiplex fluorescence *in situ* hydridization (M-FISH) with human 24-colour painting probe and DAPI-banding pattern analyses.

RNA isolation and RT-quantitative (q)PCR

Cells were grown in 12-well plates for total RNA isolation. Three wells were individually harvested per sample to obtain biological replicates. The RNeasy Mini Kit (Qiagen, #74106) together with the Qiacube was used for total RNA extraction. Cell were washed once with D-PBS then lysed with 350 µl of RLT Buffer. Each sample was treated with RNase-Free DNase (Qiagen, #79254). RNA was eluted in a volume of 30 µl. For first strand cDNA synthesis, 500 ng of RNA, random primer (Promega, #C1181) and dNTP (Promega, #U1511) were incubated for 5 min at 65°C then quickly chilled on ice. For reverse transcription of RNA, RNaseOUT Recombinant Ribonuclease Inhibitor (Invitrogen, # 10777019) and SuperScript II Reverse Transcriptase (Invitrogen, #18064014) were incubated with material obtained from the previous step in a PCR machine programmed at 10 min at 25°C for the primer annealing step, 50 min at 42°C for the extension step, and finally 15 min at 70°C for the inactivation of the enzyme. The resulting cDNA was diluted to a final volume of 600 µl with nuclease-free water prior to use for RT-qPCR. RT-qPCR master mix was prepared using Sensi Mix Sybr Low Rox Kit (Bioline, #QT625-20). RT-qPCR reactions were performed using Mx3005P

system (Stratagene) according to the manufacturer's instructions. Samples were run in technical triplicates and normalised to *PBGD*. Gene-specific primers are listed in **Supp. Table 3**.

Immunofluorescence (IF) staining

Cells in 12 well plates were fixed by aspirating the culture media then immediately adding 500 µl of 4% paraformaldehyde (PFA; VWR, #43368.9M) solution and incubating for 20 min at 4°C. They were then washed thrice in D-PBS. To block unspecific binding, cells were incubated in 500 µl of PBST (0.1% Triton X-100 in D-PBS) containing 10% donkey serum (AbD Serotec, #C06SB) per well for 20 min at room temperature. Cells were then incubated overnight at 4°C with 300 µl of primary antibodies diluted in PBST containing 1% donkey serum. Cells were next washed thrice with PBST to remove unbound primary antibodies and thereafter incubated with 300 µl of fluorescence-dye-conjugated secondary antibodies diluted in PBST containing 1% donkey serum in for 1 hr at room temperature. Unbound antibodies were removed by three 5 min washes in D-PBS. 4′,6-Diamidino-2-phenylindole dihydrochloride (DAPI; Sigma-Aldrich, #D-8417) at a dilution of 1:1000 was added to the first wash. Antibodies used for immunostaining are listed in **Supp. Table 4**.

Fluorescence activated cell sorting (FACS) analysis

Cells in 12 well plates were washed twice in D-PBS and incubated in 0.3 ml of Accutase per well for 5 min at 37°C. The Accutase was neutralised by adding 0.6 ml of 5% FBS diluted in D-PBS and the cells were dissociated by gentle pipetting. Cells were re-suspended in D-PBS at approximately 0.1-1 x 10^6 cells/ml and washed twice with D-PBS. They were then pelleted and fixed by re-suspending in 500 µl of 4% PFA solution diluted in D-PBS per well and incubating at for 20 min at 4°C, then washed twice in D-PBS. For all primary antibodies except CXCR4, cells were permeabilised in 500 µl of D-PBS containing 1% Saponin (Sigma-Aldrich, #47036-50G-F) for 30 min at room temperature. Cells were then incubated for 2 hr at room temperature with primary antibody diluted in 100 µl of Staining Solution (1% Saponin and 5% FBS in D-PBS). After which, they were washed three times with 1 ml of Staining Solution per wash and incubated with secondary antibodies diluted in 100 µl of Staining Solution for 30 min at room temperature. Unbound antibody was then removed by three washes in 1 ml of Staining Solution per wash and cells were re-suspended in 200 µl of 2% FBS diluted in D-PBS prior to analysis. For CXCR4 staining, cells were fixed in 4% PFA and washed as described above. Thereafter, primary antibody diluted in 100 µl of 5% FBS in D-PBS was added to the cells and incubated for 1 hr at room temperature. Unbound antibody was then removed by three washes of 1ml 2% FBS in D-PBS per wash. Cells were then re-suspended in 200 µl of 2% FBS in PBS prior to analysis. Analyses were performed using a BD LRSFortessa cell analyser (BD Biosciences). All flow cytometry experiments were gated using unstained cells. Data analyses were performed on FlowJo. On all flow cytometry plots, the undifferentiated population is shown in blue.

All gates shown on scatterplots were set according to the undifferentiated population control. Antibodies used for FACS analyses are listed in **Supp. Table 4**.

Western blotting

Cells were washed once in D-PBS and incubated in 0.5 ml of Accutase per well of a 6 well plate for 5 min at 37°C. The Accutase was neutralised by adding 1 ml of 5% FBS diluted in D-PBS per well and the cells were dissociated by gentle pipetting. The cells were washed twice with D-PBS and pelleted by centrifuging at 1,200 rpm. The pelleted cells were re-suspended in 50-200 µl of Lysis Buffer (50 mM Tris-Cl pH 7.5, 150 mM NaCl, 1% Triton X-100, 10% glycerol, 0.1% deocycholate, 25 mM β-glycerophosphate) containing freshly added inhibitors cOmplete Protease Inhibitor Cocktail (Roche, #11697498001), Sodium Fluoride (NaF; New England Biolabs, #P0759), Sodium Vanadate (Na_3VO_4; New England Biolabs, #P0758). The cell lysates were kept on ice for at least 15 min, vortexed at maximum speed for 15 s then centrifuged for 30 min at 15,000 g at 4°C. The supernatants were collected and protein concentrations were determined by Bradford assay (Protein Assay Dye Reagent Concentrate, Bio-Rad) according to the manufacturer's protocol. The protein concentrations of the cell lysates were normalised to 10 µg of protein for probing with *GATA6* and *GATA4* and 1 µg for probing with alpha-tubulin. The normalised cell lysates were heat denatured at 98°C in the presence of Laemmli Sample Buffer (Bio-Rad) and β-mercaptoethanol for 5 min, then subjected to SDS-PAGE electrophoresis on NuPAGE Novex 4-12% Bis-Tris Protein Gels using the XCell SureLock Mini-Cell (Invitrogen) system. The separated proteins were next transferred from the gel onto Immun-Blot PVDF membrane (Bio-Rad, #162-0177) using Mini Trans-Blot Cell (Bio-Rad) at 25 V overnight at 4°C. Membranes were blocked in 5% Blotting-Grade Blocker (Bio-Rad, #170-6404) diluted in 0.1% Triton X-100 in D-PBS (PBST) for 1 hr at room temperature. Primary antibodies were incubated for 2 hr at room temperature. Membranes were then washed and incubated with horseradish peroxidase (HRP)-conjugated secondary antibodies for 1 hr at room temperature. Unbound antibodies were removed by three 10 min washes in PBST. Proteins were detected via chemiluminescence using SuperSignal West Femto Maximum Sensitivity Substrate (ThermoFisher Scientific, #PI34095) and finally developed using Amersham Hyperfilm ECL (GE Healthcare). Antibodies used for western blotting are listed in **Supp. Table 4**.

Chromatin Immunoprecipitation (ChIP)

Co-binding of DNA to DNA-binding proteins was determined by ChIP against *GATA6* (Cell Signaling, #5851) on approximately 1 x 10^7 cells per antibody or control sample. Cells were cross-linked with 1% formaldehyde (ThermoFisher UK, #11586711) for 10 min at room temperature. The reaction was quenched with 0.125 M glycine (Millipore, #357002) for 5 min. Cells were washed twice with ice-cold PBS then collected in ice-cold PBS containing freshly-added protease inhibitors (10 µl/ml of 5 mg/ml phenylmethylsulfonylfluoride (PMSF; Sigma-Aldrich, #93482), 10 µl/ml of 1

M Sodium Butyrate (Sigma-Aldrich, #303410) and 1 µl/ml of 1 mg/ml Leupeptin (Roche, #11017101001)). Harvested cells were centrifuged for 5 min at 1,200 rpm at 4°C to pellet. For all subsequent steps, the samples were kept on ice. For all subsequent buffers used, the aforementioned protease inhibitors were added freshly to the buffers before use. The pelleted cells were subsequently re-suspended in 2 ml of ice-cold Cell Lysis Buffer (10 mM Tris-Cl pH 8.0, 10 mM NaCl and 0.2% NP-40), incubated on ice for 10 min, and then centrifuged for 5 min at 1,800 rpm at 4°C. The supernatant was discarded and the pellet was gently re-suspended in 1.25 ml of ice-cold Nuclear Lysis Buffer (50 mM Tris-Cl pH 8.0, 10 mM EDTA and 1% SDS) and incubated on ice for 10 min. 0.75 ml of ice-cold IP Dilution Buffer (20 mM Tris-Cl pH 8.0, 2 mM EDTA, 150 mM NaCl, 0.01% SDS, 1% Triton X-100) was then added.

The chromatin was sonicated using Diagenode Biorupter Pico in 15 ml Diagenode sonication tubes containing sonication beads (Diagenode, #C01020031) pre-washed with 10 ml D-PBS and 10 ml IP Dilution Buffer for 10 cycles of 30s on/45s off. Chromatin fragments were determined by a Bioanalyser (Agilent 2100 Bioanalyzer) and analysed using High Sensitivity DNA Kit (Agilent, #5067-4626) according to the manufacturer's protocol. The sonicated chromatin was then centrifuged at 14,000 rpm for 10 min at 4°C to pellet debris. 3.5 ml of IP Dilution Buffer was added to the supernatant and mixed gently. The cross-linked DNA was pre-cleared by incubating with rotation 10 µg of rabbit IgG (Sigma-Aldrich, #I5006) for 1 hr at 4°C, followed by incubating with rotation 100 µl of Protein G agarose beads (50% v/v; Roche, #11243233001) pre-washed twice with D-PBS for 1 hr at 4°C. The samples were then centrifuged for 3 min at 3,000 rpm at 4°C and the supernatant was transferred to a fresh 15 ml tube. An aliquot of 300 µl for Input sample was taken and stored at 4°C.

10 µg of *GATA6* antibody or rabbit IgG control was added per sample and incubated rotating overnight at 4°C. Antibody-bound chromatin was then collected using 60 µl of Protein G agarose beads (50% v/v) pre-washed twice with D-PBS by incubating with rotation for 1 hr at 4°C. Thereafter, the tubes were centrifuged for 3 min at 3,000 rpm at 4°C. The supernatant was discarded and the pellet containing the protein-DNA complexes bound onto the protein G agarose beads were kept.

Samples were washed twice with 500 µl of IP Wash Buffer 1 (20 mM Tris-Cl pH 8.0, 2 mM EDTA, 50 mM NaCl, 0.1% SDS and 1% Triton X-100), twice with 500 µl of IP Wash Buffer 2 (10 mM Tris-Cl pH 8.0, 1 mM EDTA, 0.25 M LiCl, 1% NP-40 and 1% Sodium deoxycholic acid), twice with 500 µl of TE Buffer (10mM Tris-Cl pH 8.0, 1mM EDTA) then eluted by washing twice with 150 µl of Elution Buffer (100 mM $NaHCO_3$ and 1% SDS). ChIP and Input DNA cross-links were reversed and RNA degraded by adding 1 µl of 1 mg/ml RNase A and 18 µl of 5M NaCl and incubating at 67°C in a heat block with shaking at 1,300 rpm overnight. Protein was degraded by adding 3 µl of 20 mg/ml Proteinase K and incubating for 3 hrs at 45°C in a heat block with shaking at 1,300 rpm. Pulled-down

genomic DNA was extracted using 300 µl of phenol/chloroform wash. The samples were next incubated with 30 µl of 3M NaAc pH 5.2 (Ambion, #AM9740), 30 µg glycoblue (Ambion, #AM9516) and 750 µl of 100% ethanol for at least 30 min at -80°C to precipitate the DNA. Precipitated DNA was pelleted by centrifuging at 14,000 rpm for 30 min at 4°C. The DNA pellet was then washed with ice-cold 70% ethanol then air dried. 70 µl of deionised water was added to Input samples whereas 30 µl of deionised water was added to ChIP samples.

RNA-seq data analysis

Tophat v2 (Kim et al., 2013) was used to align the reads to the reference human genome assembly (GRCh38/hg20), using Ensembl release 76 as reference transcriptome. featureCounts was used on paired-end reads to count fragments in annotated gene features, with parameters '-p -T 8 -t exon -g gene_id' (Liao et al., 2014). DESeq2 R/Bioconductor package was used in differential gene expression analysis between samples, requiring at least a twofold expression change and adjusted using the Benjamini–Hochberg procedure to p-value smaller than 0.01 (Love et al., 2014) for a gene to be declared as differentially expressed. The function 'rpkm' in the R/Bioconductor package edgeR (give reference) was used with default parameters to normalize count gene expression (Robinson et al., 2010). Raw bedGraphs were normalized per million mapped reads in the library per library size in all ChIP-seq and RNA-seq samples (Conesa et al., 2016; Genome Biol). Genome browser panels were generated using IGV (Thorvaldsdóttir et al., 2013). Gene Ontology (GO) analyses were performed using Amigo2 separately for up- and down- regulated differentially expressed genes (Carbon et al., 2009). Spearman's correlation values were calculated in R for FPKM expression values of genes expressed at more than 5 FPKM in at least one of the samples under comparison.

ChIP-seq data analysis

We followed recommended guidelines in the analysis of ChIP-seq data for read mapping, normalization, peak-calling and assessment of reproducibility among biological replicates (Bailey et al, 2013. PloS Comput Biol). Paired-end reads were aligned to the reference human genome assembly (GRCh38/hg20) using BWA v0.5.10 (Li and Durbin, 2009) with -q 15 and default for the rest of parameters. Reproducibility between replicates was first assessed using the Pearson Correlation Coefficient (PCC) for the two biological replicates, using the genome-wide normalized read (extended to 300 bp) count distribution on a single nucleotide resolution. For this, we used the UCSC tool bigwigCorrelate provided in http://hgdownload.cse.ucsc.edu/admin/exe/. PCC was equal to 0.949326.

Peak calling was performed using MACS version 2.0.10 (Zhang et al., 2008) allowing a p-value cut-off of 1e-3 and default settings for all other parameters. Relaxed thresholds are suggested in order to enable the correct computation of IDR values (Landt et al., 2012). Following the recommendations

for the analysis of self-consistency and reproducibility between replicates, the negative control samples (IgG and input DNA) were combined into one single control; code for IDR analysis was downloaded from https://sites.google.com/site/anshulkundaje/projects/idr (Li et al., 2011). This is also beneficial as control samples with substantially higher number of reads are recommended for peak calling (Bailey et al., 2013). 37,777 and 35,408 peaks were found for first and second replicate, respectively, with >26k of regions of direct overlap.

To estimate the Irreproducible Discovery Rate (IDR) between replicates, top 35k peaks for each biological replicate were submitted for IDR analysis. For IDR computation using MACS results, we used p-values rather than q-values as suggested in https://sites.google.com/site/anshulkundaje/projects/idr (Li et al., 2011). The number of peaks found passing a threshold of IDR ≤ 5% (12,107) was selected as a conservative estimated number of candidate transcription factor binding sites. After excluding autosomal and sex chromosomes, we have 12,098 peaks. We searched for the closest gene feature in ensembl_76_transcriptome using BEDTools closest with parameter '-D b' (Quinlan and Hall, 2010). To associate peak to genes in a 20kb window, we ran BEDTools window with '-w 20000' and own R scripts.

Co-localization plots of the transcription factors *GATA6*, *EOMES* and *SMAD2/3* ChIP-seq, was generated with deepTools (Ramirez et al., 2014). The input data was obtained by combining our ChIP data of H9 cells at day 3 (*GATA6*) with previously published *EOMES* (uploaded to Gene Expression Omnibus with accession number GSE26097) (Teo et al., 2011) and *SMAD2/3* ChIP data (uploaded to Gene Expression Omnibus with accession number GSE19461) (Brown et al., 2011). To make the results more comparable, we remapped the 3 data sets with STAR v2.5.1a (Dobin et al., 2013) (BWA failed on short single end SMAD reads) and processed them with MACS version 2.0.10 and IDR as described earlier. The resulting peak files (bed format) were used as input for deepTools. The mapped read files (bam format) were pre-processed with deepTools' "bamCompare" function (bin size = 50, assumed genome size = 2451960000 bp, ignoring chromosomes X and Y for normalization and extending single end reads by 250bp).

SUPPLEMENTARY REFERENCES

Bailey, T., Krajewski, P., Ladunga, I., Lefebvre, C., Li, Q., Liu, T., Madrigal, P., Taslim, C., and Zhang, J. (2013). Practical guidelines for the comprehensive analysis of ChIP-seq data. PLoS Comput Biol *9*, e1003326.

Bedell, V.M., Wang, Y., Campbell, J.M., Poshusta, T.L., Starker, C.G., Krug, R.G., 2nd, Tan, W., Penheiter, S.G., Ma, A.C., Leung, A.Y., *et al.* (2012). In vivo genome editing using a high-efficiency TALEN system. Nature *491*, 114-118.

Brown, S., Teo, A., Pauklin, S., Hannan, N., Cho, C.H., Lim, B., Vardy, L., Dunn, N.R., Trotter, M., Pedersen, R., *et al.* (2011). Activin/Nodal signaling controls divergent transcriptional networks in human embryonic stem cells and in endoderm progenitors. Stem Cells *29*, 1176-1185.

Carbon, S., Ireland, A., Mungall, C.J., Shu, S., Marshall, B., Lewis, S., the Ami, G.O.H., and the Web Presence Working, G. (2009). AmiGO: online access to ontology and annotation data. Bioinformatics (Oxford, England) *25*, 288-289.

Cermak, T., Doyle, E.L., Christian, M., Wang, L., Zhang, Y., Schmidt, C., Baller, J.A., Somia, N.V., Bogdanove, A.J., and Voytas, D.F. (2011). Efficient design and assembly of custom TALEN and other TAL effector-based constructs for DNA targeting. Nucleic Acids Research *39*, e82-e82.

Dobin, A., Davis, C.A., Schlesinger, F., Drenkow, J., Zaleski, C., Jha, S., Batut, P., Chaisson, M., and Gingeras, T.R. (2013). STAR: ultrafast universal RNA-seq aligner. Bioinformatics *29*, 15-21.

Doyle, E.L., Booher, N.J., Standage, D.S., Voytas, D.F., Brendel, V.P., VanDyk, J.K., and Bogdanove, A.J. (2012). TAL Effector-Nucleotide Targeter (TALE-NT) 2.0: tools for TAL effector design and target prediction. Nucleic Acids Research *40*, W117-W122.

Kim, D., Pertea, G., Trapnell, C., Pimentel, H., Kelley, R., and Salzberg, S.L. (2013). TopHat2: accurate alignment of transcriptomes in the presence of insertions, deletions and gene fusions. Genome Biol *14*, R36.

Landt, S.G., Marinov, G.K., Kundaje, A., Kheradpour, P., Pauli, F., Batzoglou, S., Bernstein, B.E., Bickel, P., Brown, J.B., Cayting, P.*, et al.* (2012). ChIP-seq guidelines and practices of the ENCODE and modENCODE consortia. Genome research *22*, 1813-1831.

Li, H., and Durbin, R. (2009). Fast and accurate short read alignment with Burrows-Wheeler transform. Bioinformatics *25*, 1754-1760.

Li, Q., Brown, J.B., Huang, H., and Bickel, P.J. (2011). Measuring reproducibility of high-throughput experiments. 1752-1779.

Liao, Y., Smyth, G.K., and Shi, W. (2014). featureCounts: an efficient general purpose program for assigning sequence reads to genomic features. Bioinformatics *30*, 923-930.
Love, M.I., Huber, W., and Anders, S. (2014). Moderated estimation of fold change and dispersion for RNA-seq data with DESeq2. Genome Biol *15*, 550.

Quinlan, A.R., and Hall, I.M. (2010). BEDTools: a flexible suite of utilities for comparing genomic features. Bioinformatics (Oxford, England) *26*, 841-842.

Ramirez, F., Dundar, F., Diehl, S., Gruning, B.A., and Manke, T. (2014). deepTools: a flexible platform for exploring deep-sequencing data. Nucleic Acids Res *42*, W187-191.

Robinson, M.D., McCarthy, D.J., and Smyth, G.K. (2010). edgeR: a Bioconductor package for differential expression analysis of digital gene expression data. Bioinformatics *26*, 139-140.

Sander, J.D., Maeder, M.L., Reyon, D., Voytas, D.F., Joung, J.K., and Dobbs, D. (2010). ZiFiT (Zinc Finger Targeter): an updated zinc finger engineering tool. Nucleic Acids Research.

Sander, J.D., Zaback, P., Joung, J.K., Voytas, D.F., and Dobbs, D. (2007). Zinc Finger Targeter (ZiFiT): an engineered zinc finger/target site design tool. Nucleic Acids Research *35*, W599-W605.

Streubel, J., Blucher, C., Landgraf, A., and Boch, J. (2012). TAL effector RVD specificities and efficiencies. Nat Biotech *30*, 593-595.

Teo, A.K., Arnold, S.J., Trotter, M.W., Brown, S., Ang, L.T., Chng, Z., Robertson, E.J., Dunn, N.R., and Vallier, L. (2011). Pluripotency factors regulate definitive endoderm specification through eomesodermin. Genes Dev *25*, 238-250.

Thorvaldsdóttir, H., Robinson, J.T., and Mesirov, J.P. (2013). Integrative Genomics Viewer (IGV): high-performance genomics data visualization and exploration. Briefings in Bioinformatics *14*, 178-192.

Zhang, Y., Liu, T., Meyer, C.A., Eeckhoute, J., Johnson, D.S., Bernstein, B.E., Nusbaum, C., Myers, R.M., Brown, M., Li, W.*, et al.* (2008). Model-based analysis of ChIP-Seq (MACS). Genome Biol *9*, R137.
